# Effects of different exercise interventions on depression and anxiety in cancer survivors: a systematic review and Bayesian network meta-analysis of randomized controlled trials

**DOI:** 10.3389/fonc.2026.1741755

**Published:** 2026-04-20

**Authors:** LongYu Nie, Min Liu, ZhiDuo Chen, ChuanKai Luan, DingWu Liu, BingAo Chen, JinYu Wang, ChuanPing Lei

**Affiliations:** 1 School of Physical Education, Qufu Normal University, Qufu, China; 2 School of Physical Education, Shandong Sport University, Jinan, China; 3 School of Physical Education, Shandong University, Jinan, China; 4 School of Chinese Language and Literature, Qufu Normal University, Qufu, China

**Keywords:** anxiety, Bayesian network meta-analysis, cancer, depression, exercise, mental health

## Abstract

**Background:**

We conducted a network meta-analysis to compare and rank exercise modalities for alleviating depression and anxiety in cancer survivors, estimate optimal doses, and provide prescribing guidance.

**Methods:**

We synthesized randomized controlled trials published through July 2025 using an arm-based Bayesian multilevel network meta-analysis (NMA) with Hedges’ g and the surface under the cumulative ranking curve (SUCRA). We assessed risk of bias using Risk of Bias 2.0 (RoB 2.0) and evaluated certainty of evidence using the Grading of Recommendations Assessment, Development and Evaluation (GRADE) approach.

**Results:**

Ninety-four RCTs (n = 7,668) were included. Versus non-exercise controls, exercise produced small-to-moderate improvements in depression and anxiety. For depression, Pilates (g = −0.35; 95% credible interval (CrI),−0.61 to −0.10; k = 3; SUCRA = 0.78), Tai Chi and Qi gong (g = −0.32; 95% CrI, −0.48 to −0.17; k = 11; SUCRA = 0.77), as well as aerobic exercise (g = −0.25; 95% CrI,−0.37 to −0.13; k = 24; SUCRA = 0.75) appeared to rank highly; favorable dose-response patterns were observed around approximately 2.8 metabolic equivalents (METs), 40 minutes per session, and six sessions per week. For anxiety, Pilates (g = −0.70; 95% CrI, −1.16 to −0.26; k = 1; SUCRA = 0.84) and high-intensity interval training (HIIT) (g = −0.36; 95% CrI,−0.66 to −0.05; k = 2; SUCRA = 0.79) were promising but based on few small RCTs, whereas Tai Chi and Qi gong (g = −0.35; 95% CrI,−0.51 to −0.19; k = 10; SUCRA = 0.69) and aerobic exercise (g = −0.27; 95% CrI,−0.40 to −0.15; k = 21; SUCRA = 0.62) showed stable benefits in larger samples. Dose-response suggested relatively favorable anxiety benefits with moderate intensity and duration at approximately five sessions per week. For both outcomes, effects plateaued around 20 weeks without additional gains at follow-up. Higher BMI and older age may attenuated effects; the female proportion showed no statistically significant association. Overall certainty tended to be low owing to risk of bias, small-study effects, and imprecision.

**Conclusions:**

Mind-body exercises, especially Tai Chi and Qi gong, may be considered first because they were supported by a larger and more stable evidence base. Aerobic exercise also showed consistent benefits. Although Pilates and HIIT showed favorable estimated effects in some analyses, these findings should be interpreted cautiously because the certainty of evidence was low to very low. The recommended prescription for depression is approximately 2.8 METs, 40 minutes per session, six sessions per week; however, this estimate should be interpreted cautiously and individualized rather than applied as a fixed prescription. For anxiety, the recommended prescription is moderate-intensity, moderate-duration exercise five times weekly. Both treatment courses last approximately 20 weeks.

**Systematic review registration:**

https://www.crd.york.ac.uk/PROSPERO/view/CRD420251110539, identifier CRD420251110539.

## 1 Introduction

Depression and anxiety represent a major source of global disease burden (1). In 2021, approximately 332 million people worldwide had depression (2), while about 359 million experienced anxiety disorders (3). The impact of these disorders on nonfatal health loss continues to rise, impairing quality of life and social functioning. Among patients with cancer, the psychological burden is particularly pronounced. A multicenter study using a stratified national sample in Germany reported a 4-week prevalence of “any mental disorder” of 31.8% (4). Systematic reviews and meta-analyses further indicate that depression comorbidity among cancer patients typically ranges from 8% to 24%, varying by cancer type, treatment stage, and measurement tools (5). Critically, this risk does not diminish over time. A UK electronic health record–based matched cohort study across 20 high-incidence cancers found that cancer survivors face an elevated risk of new-onset anxiety and depression even in the mid- to long-term post-diagnosis period. For instance, adjusted hazard ratios for anxiety ranged from approximately 1.18 to 2.94, and for depression from approximately 1.12 to 2.98 (6). Spouses likewise bear a substantial psychological burden, underscoring the need for long-term, scalable symptom management and follow-up services (7).

Contemporary oncology guidelines recommend routine screening for anxiety and depression in patients with cancer and prioritize evidence-based psychotherapy, with collaborative care and medication when necessary. However, real-world effectiveness and accessibility remain limited (8). Psychological interventions typically yield small-to-moderate effects (9), and collaborative care models (e.g., SMaRT and DCPC) require multidisciplinary integration, intensive follow-up, and information system support (10), limiting scalability under resource constraints. The benefits of antidepressants remain uncertain in cancer populations, with few direct head-to-head comparisons (11). In contrast, exercise interventions offer notable advantages: scalability, lower cost, minimal drug interactions, and concurrent improvements in fatigue and sleep quality (12). Recent meta-analyses of randomized controlled trials in oncology have shown small-to-moderate improvements in depression and anxiety, alongside enhanced HRQOL, among adults with cancer and breast or lung cancer survivors (13–15). Mind–body therapies such as yoga, Tai Chi, and Qi gong also show favorable effects on emotional well-being (16, 17). For cancer survivors, regular exercise activates the skeletal muscle PGC-1α1–KAT axis, converting KYN to KYNA and reducing the tumor- and treatment-induced neurotoxic KYN load, thereby alleviating depressive or anxious symptoms (18). Simultaneously, exercise reshapes the gut microbiota and its metabolite profile, influencing central plasticity and neuroimmune interactions via the vagus–immune–endocrine axis, thereby synergistically alleviating depressive and anxious symptoms (19).

Although evidence supports exercise for improving depression and anxiety in patients with cancer, most studies focus on single cancer types and single exercise prescriptions (20). Accordingly, the generalizability of findings across different cancer types and exercise prescriptions remains limited, because external validity ultimately depends on the populations represented in the primary studies (21). Even in meta-analyses comparing multiple exercise types, few studies standardize and quantify exercise dose, including intensity, duration, and frequency, to develop continuous dose–response models (22, 23). Concurrently, reporting of adherence and intervention fidelity remains insufficient, undermining the robustness of effect estimates (24, 25).

Accordingly, we conducted a Bayesian multi-arm network meta-analysis to compare exercise intervention patterns for depression and anxiety symptoms among cancer survivors. This approach explicitly modeled within-trial correlations and shared control groups in the multi-arm design, enforced consistency constraints across the network, and addressed between-study heterogeneity with a random-effects model. Concurrently, we fit Bayesian nonlinear dose–response models, treating dose as a continuous exposure and linking it to the network meta-analysis. This approach allowed us to compare intervention patterns and explore pooled dose–response trends across the available evidence, while recognizing that the external validity of these findings remains dependent on the cancer populations represented in the included primary trials.

## 2 Methods

### 2.1 Protocol and registration

This study was prospectively registered in PROSPERO (CRD420251110539). The review follows PRISMA 2020 and uses the PRISMA-NMA extension for reporting network meta-analyses (26). Methods also follow the Cochrane Handbook for Systematic Reviews of Interventions (27). Because all data were obtained from published studies, ethical approval was not required.

### 2.2 Eligibility criteria

We predefined inclusion and exclusion criteria based on PICOS principles: (1) Participants: Adults (≥18 years) with a histologically or cytologically confirmed malignant neoplasm (solid or hematologic) were included; “cancer survivor” refers to any individual from diagnosis through the remainder of life, including those receiving active treatment and those in the post-treatment phase (28). Because treatment phase was not consistently reported in sufficient detail across all included trials, we retained this broad survivorship definition for study eligibility, but considered treatment status as a potential source of clinical heterogeneity in the analysis. Participants had to be cleared for at least low- to moderate-intensity exercise without contraindications. Neither a formal psychiatric diagnosis of depression or anxiety (e.g., DSM- or ICD-based criteria) nor a predefined minimum baseline cut-off on a validated symptom scale was required for study eligibility; however, included trials had to report depression and/or anxiety outcomes using validated instruments. (2) Interventions: Eligible interventions included aerobic, resistance, aerobic + resistance, high-intensity interval training, Pilates, Tai Chi and Qi gong, yoga, and mixed exercise (Mixed exercise was defined as an intervention that combined two or more distinct exercise modalities within the same program, excluding the predefined aerobic + resistance category). (3) Comparison: Eligible trials included exercise vs exercise head-to-head comparisons (e.g., aerobic vs resistance, combined vs mind-body, or different intensities, doses, or formats) and exercise vs non-exercise comparators (e.g., usual activities, health education, waiting list, or standard care). (4) Outcomes: Primary measures included the Hospital Anxiety and Depression Scale (HADS-A and HADS-D), Generalized Anxiety Disorder Scale (GAD-7), Patient Health Questionnaire (PHQ-9), Beck Depression Inventory (BDI), State-Trait Anxiety Inventory (STAI-S and STAI-T), Center for Epidemiologic Studies Depression Scale (CES-D), and Depression Anxiety Stress Scale (DASS-21). All validated scales were harmonized to the same direction before analysis, with higher scores indicating worse depression or anxiety symptoms. Trials had to provide data enabling effect size calculation (mean, standard deviation, or equivalent statistics such as SE or 95% CI). (5) Study design: Only randomized controlled trials (RCTs) were included; conference abstracts, research protocols, and systematic reviews were excluded. Studies with insufficient data extraction or unavailable data were also excluded.

### 2.3 Search strategy and study selection

We searched Web of Science, PubMed, Embase, and the Cochrane Library. We searched from database inception through July 2025 without language restrictions. The strategy combined medical subject headings, terms, and keywords, including “Neoplasms,” “cancer,” “carcinoma,” “tumor,” “malignant,” “oncology,” “cancer survivor,” “Exercise,” “Exercise Therapy,” “Physical Activity,” “physical training,” “Pilates,” “aerobic exercise,” “Depression,” “Anxiety,” and “related synonyms” (**Supplementary Material 1**). We used EndNote X9 to remove duplicates and manually searched reference lists of prior systematic reviews and meta-analyses to ensure all potentially eligible studies were captured. After de-duplication, two reviewers independently screened titles and abstracts, followed by full-text assessment of potentially eligible studies. Any disagreements were resolved through discussion, and when necessary, a third experienced reviewer made the final decision.

### 2.4 Data extraction

Two independent authors extracted data from eligible studies. A third experienced author resolved any disagreements. Extracted data included publication year, first author, patient characteristics (gender ratio, sample size, mean age and standard deviation, mean BMI and standard deviation, cancer type, stage, treatment status), intervention details (type, duration), anxiety and depression outcomes, and risk-of-bias information. To minimize reporting bias across scales, we extracted all validated anxiety and depression outcomes from all reported scales. We also documented intervention duration and each study’s follow-up time points.

### 2.5 Data coding and management

We used exercise intensity per session, exercise duration, and exercise frequency to represent the dose–response relationship across exercise modalities. Metabolic equivalents (METs) provide a standardized measure of exercise intensity and energy expenditure (29). We coded intensity according to the 2024 Adult Compendium of Physical Activities and the American College of Sports Medicine Metabolic Calculation Manual (30, 31). In this study, MET refers to exercise intensity expressed as a multiple of the resting metabolic rate. We coded intensity (in METs), session duration (minutes), and weekly frequency to construct dose–response models. When MET values were not directly reported, we estimated intensity from the exercise prescription details whenever possible; if the available information was insufficient, the study was excluded from the corresponding dose–response analysis. For mixed-exercise interventions, we calculated a session-level MET value using a time-weighted approach based on the duration of each exercise component.

### 2.6 Risk of bias and certainty of evidence

We used the Cochrane Risk of Bias 2 (ROB 2) tool to assess risk of bias, evaluating five domains per outcome: D1 randomization process; D2 deviations from intended interventions; D3 missing outcome data; D4 measurement of the outcome; D5 selection of the reported result. We made an overall judgment for each outcome (low risk, some concerns, or high risk) (32). Two independent reviewers, LYN and CKL, assessed risk of bias in the 94 included studies. A third senior reviewer, ML, resolved any disagreements. We assessed certainty of evidence for each outcome using GRADE (33).

### 2.7 Statistical analysis

To ensure comparability, we pooled data using pre- and post-intervention means and standard deviations (SD). We used the R package “esc” to compute standardized mean differences (SMD) (34), with Hedges’ g as the effect size measure, which corrects for small-sample bias (35). Negative Hedges’ g values indicate symptom improvement, reflecting greater reductions in depression or anxiety in the exercise group relative to the control group. Studies reporting only post-intervention values were excluded from this analysis. For studies reporting change scores, effect sizes were calculated directly from the reported mean changes and corresponding standard deviations. When SD was not reported, we estimated it from the standard error (SE) or the 95% confidence interval (CI). Effect sizes were interpreted using standard thresholds: small (0.20 ≤ g< 0.50), moderate (0.50 ≤ g< 0.80), and large (g ≥ 0.80) (36).Because pre–post correlations were not consistently reported across trials, we conducted sensitivity analyses using alternative assumed correlation coefficients (r = 0.20 and r = 0.80) to evaluate the robustness of the depression and anxiety findings.

The primary analysis employed an arm-based Bayesian multilevel random-effects model that hierarchically nested multiple effect sizes (e.g., from different scales or time points) within studies to explicitly account for effect-size dependency. To enhance model stability and generalization, we used weakly informative priors (e.g., normal priors on intercepts and half-Cauchy priors on scale parameters) (37), and ran eight parallel MCMC chains using Hamiltonian Monte Carlo. Each chain ran 6,000 iterations (3,000 warm-up, adaptation and 3,000 retained), yielding 24,000 post–warm-up draws in total (38). Convergence was assessed using $\hat{\text{R}}<1.05$, trace and density plots, effective sample sizes, and MCSE-to-SD< 10% (39). For ranking, we reported the posterior mean and its 95% CrI for each intervention, along with results based on the surface under the cumulative ranking curve (SUCRA); SUCRA ranges from 0 to 1 (0–100%), with higher values indicating a greater probability of superior efficacy. A SUCRA score of 0 indicates the least effective intervention across all comparisons (40). Before conducting indirect comparisons, we assessed the plausibility of the transitivity assumption clinically. We examined whether key potential effect modifiers were reasonably comparable across treatment comparisons, including mean age, BMI, female proportion, cancer type, cancer stage, treatment status, intervention duration, and follow-up time points. These variables were extracted and reviewed across the network to assess clinical comparability; however, because treatment phase was incompletely and inconsistently reported, residual phase-related intransitivity could not be excluded. Subsequently, we evaluated global and local consistency. Global network consistency was assessed using a design-by-treatment interaction model, and local consistency was assessed with node-splitting to compare direct and indirect evidence. When necessary, we referenced consistency and inconsistency modeling approaches for multi-arm studies to identify potential bias from design-by-treatment interactions (41, 42). To examine whether concurrent radiotherapy or chemotherapy moderated the effect of exercise interventions on depression and anxiety, we conducted Bayesian random-effects subgroup analyses. All effect sizes are reported as standardized mean differences (Hedges’ g). A negative value indicates greater symptom improvement. The net effect was calculated by subtracting the pooled control group estimate from each exercise subgroup estimate.

To examine the nonlinear relationship between exercise intensity and dose, as well as the potential optimal dose or range, we modeled the dose-response function using natural cubic splines with four knots in a Bayesian multilevel framework. Knot locations were selected using a quantile-based approach according to the distribution of the observed dose values. Different intensity levels were treated as multiple “dose points” within each study and modeled hierarchically to improve the stability and accuracy of curve fitting. The use of four knots represented a practical balance between flexibility and parsimony, allowing us to capture potential nonlinear patterns without introducing excessive model complexity or overfitting. We used the curves and corresponding dose tables to identify the “optimal dose and range” at which efficacy plateaued or approached its peak, representing the optimal therapeutic effect (43).

## 3 Results

### 3.1 Study selection

**Figure 1:**
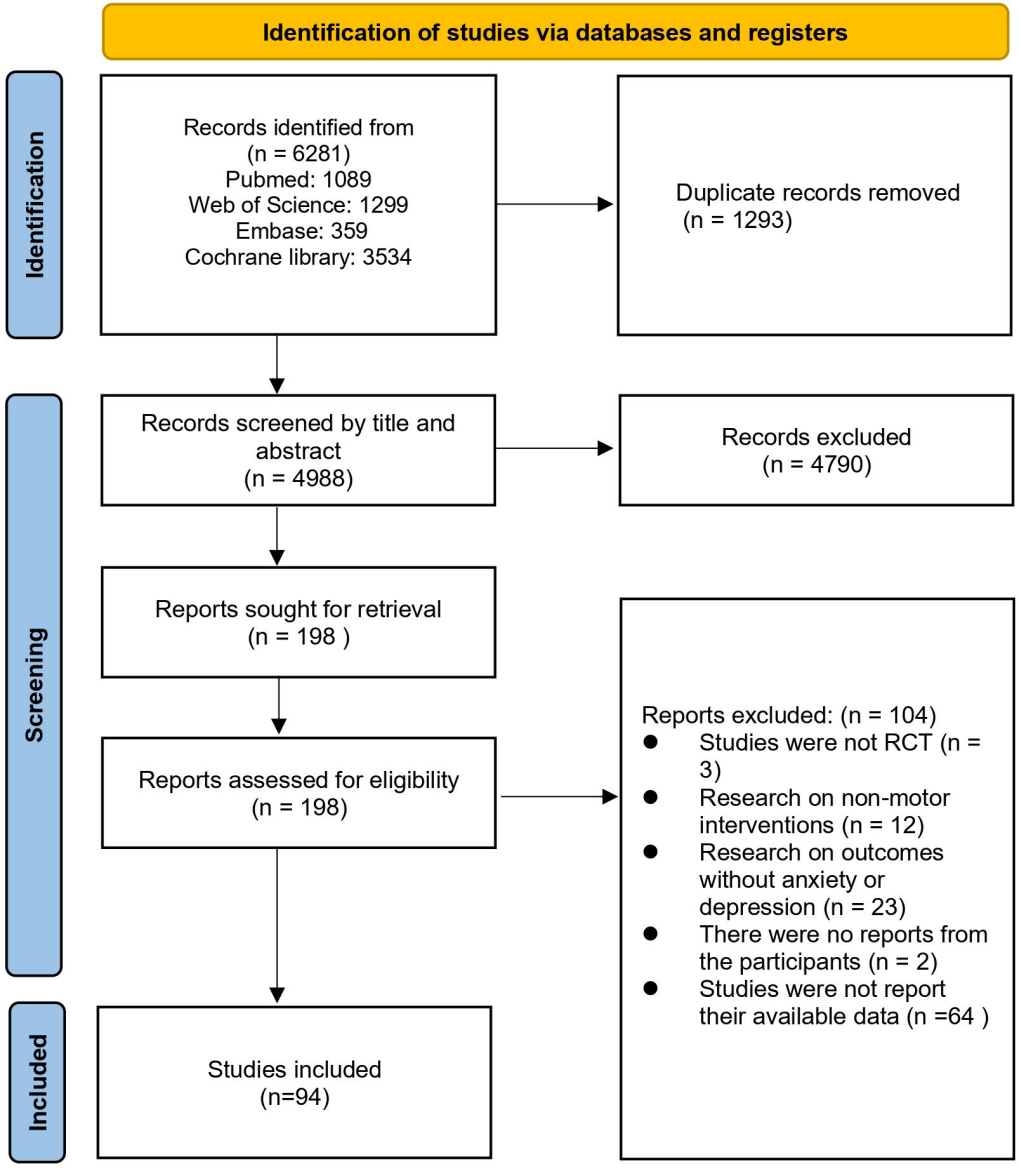
Literature review flowchart.

We retrieved 6,281 records from the four databases. After screening 4,988 titles and abstracts, 94 studies met eligibility criteria and were included in the network meta-analysis. **Figure 1** summarizes the study selection process.

### 3.2 Description of clinical trials

This analysis included 7,668 participants, with women comprising the majority (5,729; 74.7%). Mean age across studies ranged from 20.3–71.9 years. Participants were drawn from mixed clinical contexts, including active treatment and post-treatment survivorship; however, treatment phase and performance status were inconsistently reported across trials, precluding a uniform phase-based characterization of the included populations. We included 94 trials published between 2007 and 2025. Exercise modalities were: aerobic (25 studies), aerobic + resistance (24), HIIT (4), resistance (11), mixed (two or more interventions; 7), Pilates (3), Tai Chi and Qi gong (13), and yoga (22). Because some trials had multiple exercise arms, category counts exceed the number of unique studies. Each session lasted 12–120 minutes; frequencies ranged from 1–14 sessions per week, and durations spanned 1–52 weeks. Detailed participant characteristics by group, including cancer type, and effect-size forest plots are provided in **Supplementary Materials 2**, **3**, and **4**.

### 3.3 Risk of bias and quality of evidence

The risk-of-bias assessment was conducted and reported separately by outcome. For anxiety outcomes, 52 studies were at high risk and 11 had some concerns. For depression outcomes, 69 studies were at high risk and 19 had some concerns (**Supplementary Material 5**). This pattern likely reflected, at least in part, the practical difficulty of blinding participants and intervention personnel in exercise trials, together with the frequent use of self-reported psychological outcomes, particularly in the domains of deviations from intended interventions and measurement of the outcome.

### 3.4 Multilevel network meta-analysis

#### 3.4.1 Depression

Global design-by-treatment tests showed no evidence of inconsistency. The graph-theoretical between-designs Q test was non-significant (Q = 20.87, df=21, p=0.4669), and the random-effects design-by-treatment specification likewise showed no global inconsistency (Q = 13.13, df=21, p=0.9041). Node-splitting analyses for depression found no local inconsistency across 15 comparisons under a random-effects SIDE model (p>0.05). (**Supplementary Material 6**) These findings support network coherence for depression. Clinical transitivity was considered plausible based on the distribution of key effect modifiers across comparisons.

**Figure 2:**
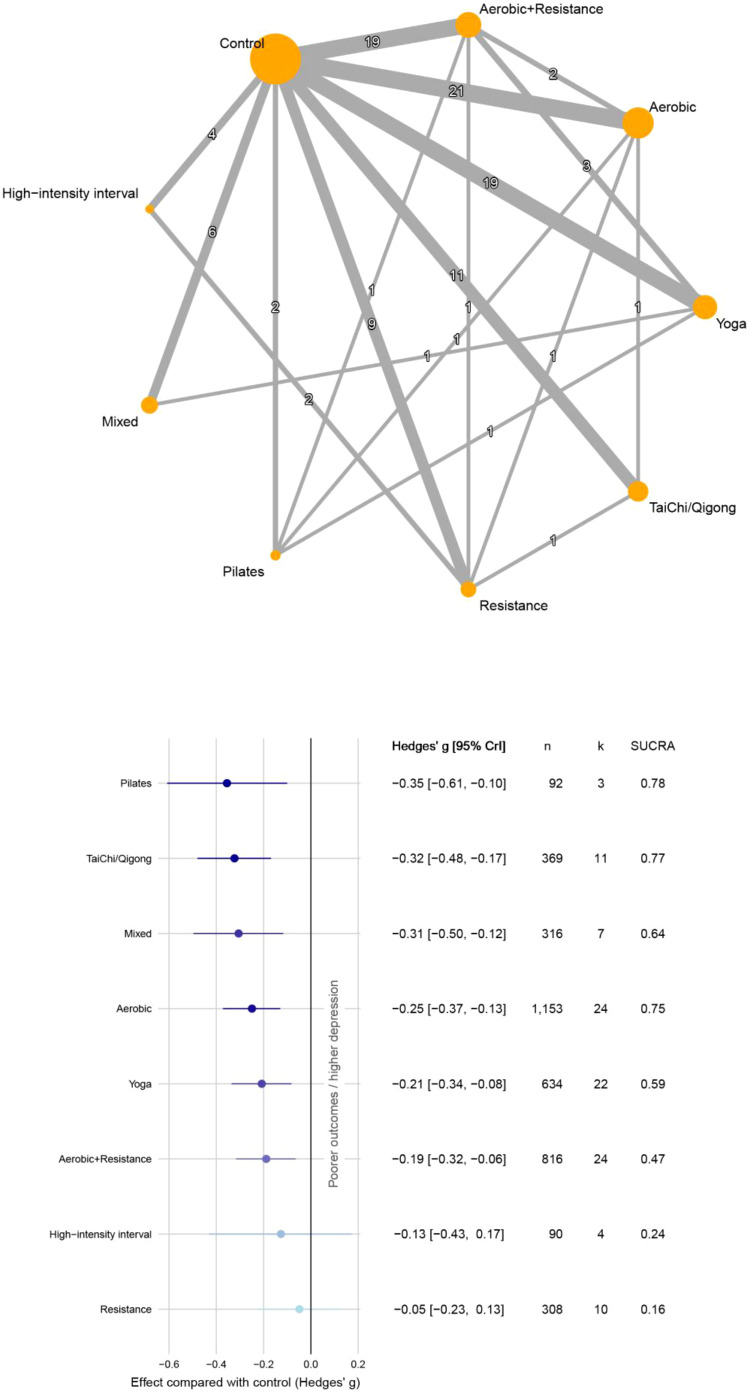
Network diagrams and forest plots (depression).

**Figure 2** shows network comparisons of exercise interventions for depressive symptoms in cancer survivors and the cumulative rankings. Pilates showed the strongest effect versus controls (g=-0.35; 95% CrI, -0.61 to -0.10; k=3; SUCRA = 0.78). This was followed by Tai Chi and Qi gong (g=-0.32; 95% CrI, -0.48 to -0.17; k=11; SUCRA = 0.77) and aerobic exercise (g=-0.25; 95% CrI, -0.37 to -0.13; k=24; SUCRA = 0.75). The funnel plot showed marked asymmetry; Egger’s regression (p<0.001) and Begg’s test (p=0.001) indicated small-study effects consistent with publication bias (**Supplementary Material 7**).

#### 3.4.2 Anxiety

Global design-by-treatment interaction tests showed no incoherence. The graph-theoretical between-designs Q test was non-significant (Q = 7.18, df=13, p=0.8926), and the random-effects design-by-treatment model likewise showed no global inconsistency (Q = 6.56, df=13, p=0.9236). Node-splitting analyses for anxiety found no local inconsistency across 12 comparisons under a random-effects SIDE model (p>0.05) (**Supplementary Material 8**). These results support network coherence for anxiety. Clinical transitivity was considered plausible based on the distribution of key effect modifiers across comparisons.

**Figure 3:**
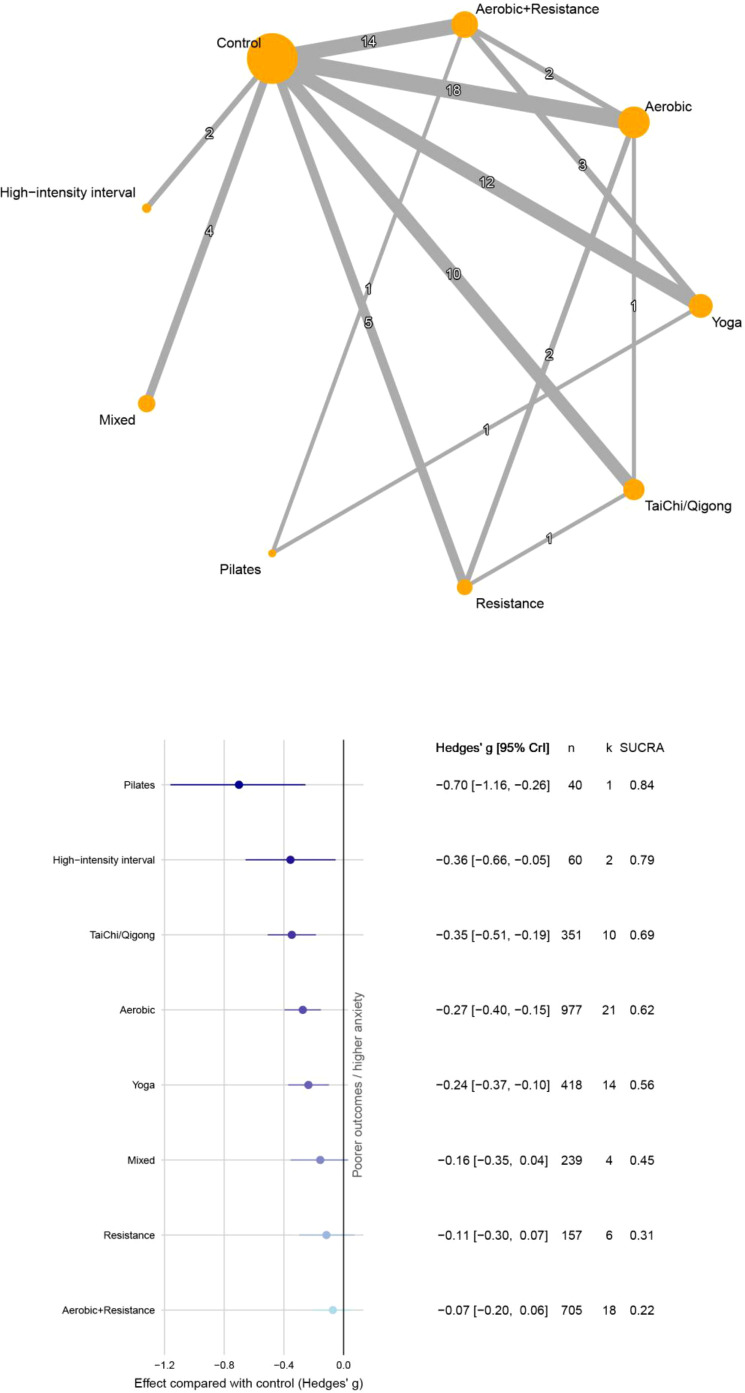
Network diagrams and forest plots (anxiety).

**Figure 3** shows network comparisons and cumulative rankings for anxiety in cancer survivors. Versus control, Pilates (g=-0.70; 95% CrI, -1.16 to -0.26; k=1; SUCRA = 0.84) and HIIT (g=-0.36; 95% CrI, -0.66 to -0.05; k=2; SUCRA = 0.79) rank near the top; however, both estimates are based on few randomized trials and small samples. Tai Chi and Qi gong (g=-0.35; 95% CrI, -0.51 to -0.19; k=10; SUCRA = 0.69) and aerobic exercise (g=-0.27; 95% CrI, -0.40 to -0.15; k=21; SUCRA = 0.62) rank next. Funnel plot asymmetry and statistically significant Egger regression results (p<0.001) suggest potential publication bias (**Supplementary Material 9**).

#### 3.4.3 Subgroup analysis by concurrent radiotherapy or chemotherapy status

Among exercise groups, patients who were concurrently receiving radiotherapy or chemotherapy showed a significant reduction in depressive symptoms compared with controls (SMD = −0.302, 95% CrI [−0.413, −0.196]), representing a small-to-moderate effect. In contrast, patients not receiving concurrent treatment showed a negligible and non-significant effect (SMD = −0.032, 95% CrI [−0.283, 0.224]). The between-subgroup difference was −0.270 (95% CrI [−0.538, −0.010]), with the credible interval excluding zero, indicating a credible moderating effect. A similar pattern was observed for anxiety, though the between-subgroup contrast was smaller. Patients receiving concurrent radiotherapy or chemotherapy demonstrated a significant reduction in anxiety (SMD = −0.289, 95% CrI [−0.399, −0.177]). Patients without concurrent treatment showed a non-significant effect (SMD = −0.219, 95% CrI [−0.507, 0.062]). The between-subgroup difference was −0.069 (95% CrI [−0.359, 0.226]), with the credible interval including zero, suggesting that concurrent treatment status did not credibly moderate the anxiolytic effect of exercise (**Supplementary Material 10**).

Taken together, the subgroup analyses suggest that exercise interventions may be effective for reducing depression in cancer patients who are concurrently receiving radiotherapy or chemotherapy, with evidence of between-subgroup heterogeneity. For anxiety, both subgroups showed a similar direction of effect, but there was no clear evidence that concurrent treatment status modified the anxiolytic effect of exercise.

#### 3.4.4 Sensitivity analyses

Sensitivity analyses using alternative assumed pre–post correlation coefficients (r = 0.20 and r = 0.80) showed findings broadly consistent with the primary analyses for both depression and anxiety. Across these alternative assumptions, the direction and magnitude of the pooled effects changed only minimally, and the overall pattern of intervention rankings remained largely unchanged. These results suggest that the main findings were not materially driven by the choice of assumed correlation coefficient (**Supplementary Material 11**).

### 3.5 Dose-response analysis

#### 3.5.1 Depression

**Figure 4:**
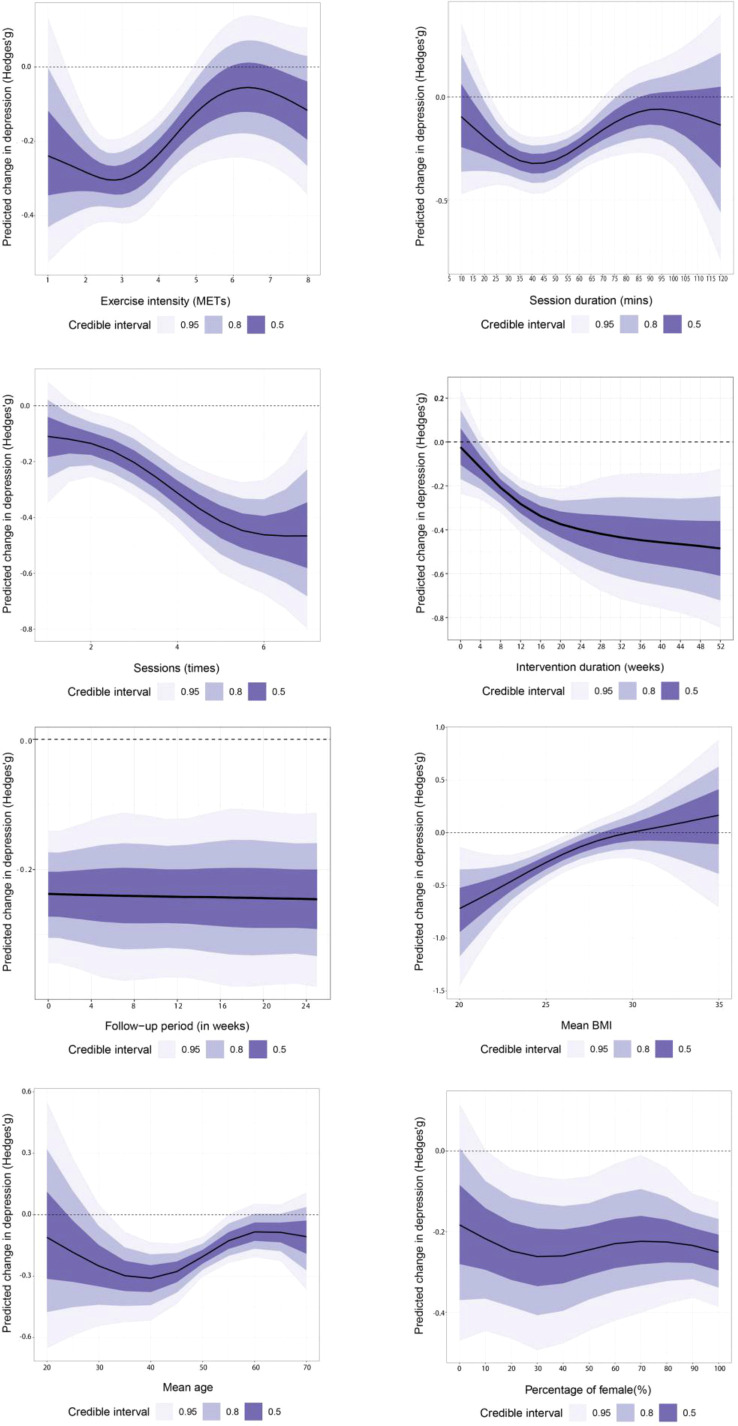
Dose-response relationship (depression).

The intensity–effect curve rises with intensity and then plateaus; the optimal intensity is approximately 2.8 METs (Hedges’ g=−0.31; 95% CrI, −0.40 to −0.21).This value should be interpreted cautiously as a pooled reference point rather than a universal physiological optimum. A single-session duration of 40 minutes (Hedges’ g=−0.32; 95% CrI, −0.44 to −0.21) and a frequency of six sessions per week (Hedges’ g=−0.47; 95% CrI, −0.64 to −0.29) yielded optimal effects (**Supplementary Material 12**). For duration, approximately 20 weeks was optimal; beyond 20 weeks, wide prediction intervals precluded a clear dose–response relationship. Modeling follow-up time as a continuous moderator produced flat curves with wide credible intervals, indicating minimal impact of assessment timing. Higher BMI and older age were associated with smaller benefits, whereas female proportion showed no evidence of moderation (**Figure 4**). R^2^ density plots showed only marginal gains from adding interaction terms, indicating that no moderators substantially improved model fit (**Supplementary Material 13**).

#### 3.5.2 Anxiety

**Figure 5:**
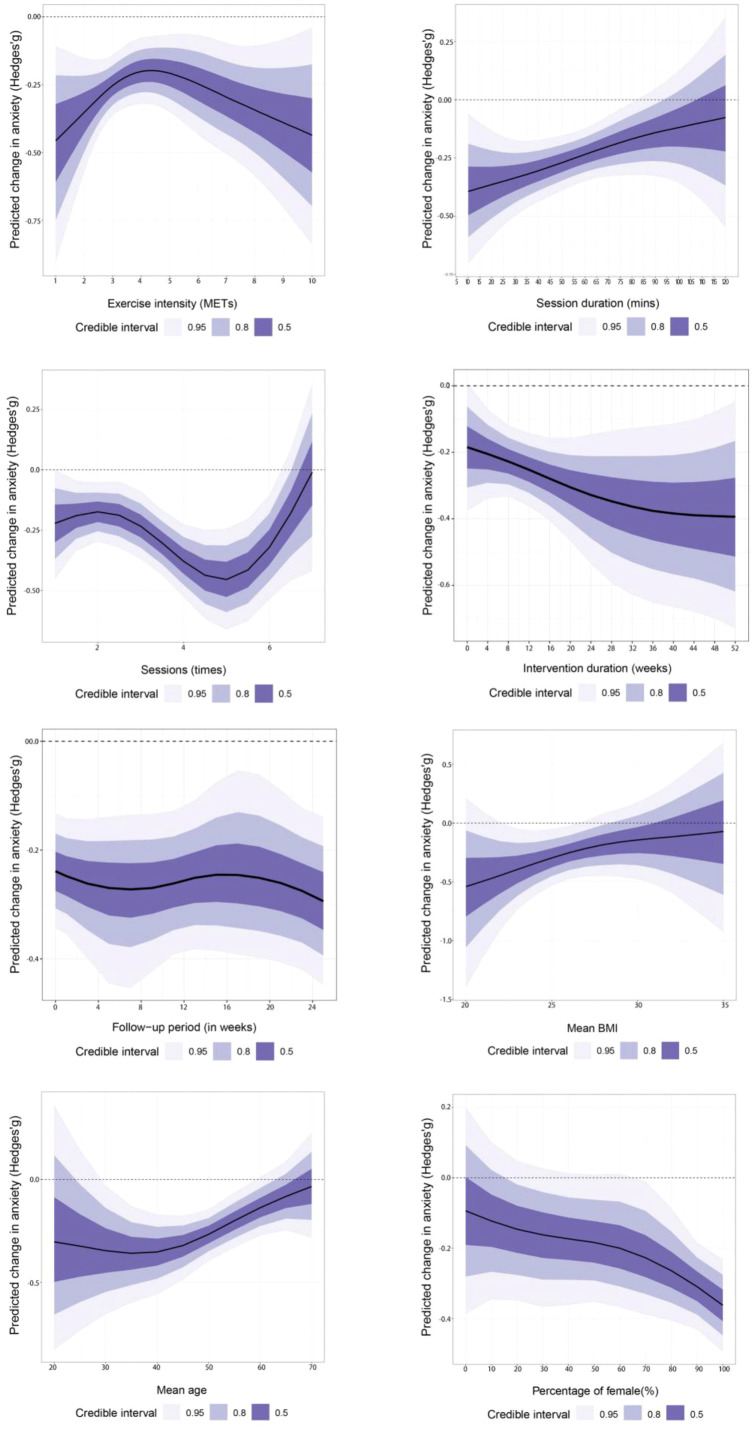
Dose-response relationship (anxiety).

Although wide credible intervals preclude precise optima, the dose–response curve shows an overall decline in anxiety. For session duration, a moderate single-session length was most effective, with shorter durations yielding inconsistent results and longer sessions showing diminishing benefits and greater uncertainty; training five times per week yielded the best results (Hedges’ g=−0.45; 95% CrI, −0.63 to −0.28) (**Supplementary Material 14**). An intervention of approximately 20 weeks maximized benefits; longer durations offered negligible additional gains while increasing uncertainty, with follow-up time curves flat and credible intervals wide, indicating minimal influence of assessment timing. Higher baseline BMI and older age were associated with smaller benefits (**Figure 5**). The intervention’s effect on anxiety was largely unchanged across female proportions from 0% to 100%, indicating no evidence of moderation by female proportion. After adding interaction terms, R^2^ density plots overlapped with the baseline model, indicating that no moderators substantially improved model fit (**Supplementary Material 15**).

## 4 Discussion

### 4.1 Principal findings

In this meta-analysis of randomized controlled trials in cancer survivors, mind-body exercise and aerobic exercise appeared relatively favorable for improving depression and anxiety symptoms. For depression, Pilates, Tai Chi and Qi gong, and aerobic exercise ranked highly in the network estimates, although the dose–response peak around 2.8 METs should be interpreted as a pooled reference point rather than a fixed optimal prescription. For anxiety, Tai Chi and Qi gong and aerobic exercise appeared to provide more stable benefits based on a larger evidence base, whereas Pilates and HIIT showed favorable estimated effects but were supported by fewer trials. Benefits for both outcomes tended to plateau at approximately 20 weeks, and higher BMI and older age were associated with smaller improvements. Certainty of evidence was generally low to very low across many comparisons (**Supplementary Material 16**), which should be considered when interpreting the network rankings and dose–response findings.

### 4.2 Compared to other studies and future directions

This study found that exercise interventions can improve anxiety and depression among cancer survivors. For depression, Pilates, Tai Chi and Qi gong, followed by aerobic exercise, ranked highly in the network estimates. For anxiety, Tai Chi and Qi gong and aerobic exercise appeared to provide more stable benefits based on a larger evidence base, whereas Pilates and HIIT showed favorable rankings but were supported by fewer trials. These findings suggest that exercise types offer distinct advantages for psychological symptoms (44), align with current oncology health-management guidelines, and support the non-pharmacological strategies advocated in clinical practice guidelines (45).

Our network meta-analysis showed that Tai Chi and Qi gong alleviated anxiety and depression in cancer survivors and improved quality of life, consistent with recent reviews and meta-analyses (46). However, individual trials often reported small-to-moderate between-group effects or no statistically significant differences, with greater antidepressant effects when combined with functional training. These variations may reflect differences in trial quality, baseline symptom severity, and adherence (47, 48). Mechanistically, low- to moderate-intensity continuous movements combined with mindful breathing may regulate autonomic balance and influence inflammatory–neuroendocrine pathways (49). Supporting this view, a recent systematic review and meta-analysis reported beneficial effects of Tai Chi on several HRV-related indices, particularly in interventions that explicitly emphasized breathing (50). Taken together, these findings provide a plausible physiological basis for the favorable psychological effects of mind-body exercise observed in our study.

In this Bayesian network meta-analysis, Pilates and HIIT showed high rankings for some outcomes. However, these rankings may overstate the certainty of benefit because the underlying evidence came from few small trials, with wide credible intervals and possible small-study effects. Therefore, these findings should be viewed as preliminary rather than practice-defining. Multiple randomized trials in breast cancer have shown that Pilates improves mood in intervention groups (51, 52), whereas one colorectal cancer quasi-experimental study found no statistically significant difference in depression improvement versus standard care (53). This inconsistency may reflect limited statistical power, variation in assessment scales, and differences in intervention dose. A possible explanation is that breathing-focused Pilates may contribute to psychological improvement through autonomic regulation and respiratory-related pathways, including enhanced vagal activity and heart rate variability, improved baroreflex sensitivity and cardiorespiratory coupling, and better thoracic compliance and respiratory muscle function (54–57), However, these physiological mechanisms should be considered supportive rather than confirmatory, and they do not resolve the statistical uncertainty associated with the small and heterogeneous evidence base.

We observed more robust evidence supporting moderate-intensity continuous aerobic exercise for alleviating depression and anxiety (58). A 2024 JAMA Network meta-analysis (25 RCTs) found that aerobic activity reduced depression and anxiety both at intervention completion and at 6–12-month follow-up (59). Although evidence for HIIT in cancer survivors remains uncertain, the ERASE randomized trial in prostate cancer “active surveillance” populations found that 12 weeks of HIIT reduced cancer-specific anxiety and fear of recurrence, with good safety and adherence (60). Related reviews also suggest that HIIT improves quality of life and multiple symptoms while maintaining acceptable safety (61). A potential mechanism is “intensity-driven neuroplasticity”: high-intensity aerobic exercise more readily upregulates the BDNF and PGC-1α–FNDC5 (irisin) axis (62), accelerating beneficial remodeling of emotional circuits via the “lactate–hippocampus–BDNF” pathway (63).

Participants with higher BMI and older age showed smaller improvements in anxiety and depression; gender was not a consistent moderator. Higher BMI often co-occurs with chronic low-grade inflammation, insulin resistance, and reduced vagal tone (lower HRV), which may blunt exercise-induced anti-inflammatory responses and neurotrophic adaptations (64, 65). As age advances, declines in neuroplasticity, greater comorbidity burden, and increased fatigue can limit achievable training doses and benefits, yielding overall smaller effects (66). Consistent with this, Kenkhuis et al. (67) reported that demographic variables, including age and gender, exerted only limited or negligible modulation of the emotional effects of exercise interventions. Therefore, for high-BMI and older-adult subgroups, more refined intensity–volume prescriptions, phased progression strategies, and long-term behavioral support (e.g., supervision, follow-up, and adherence promotion) should be employed to maximize benefits.

Most meta-analyses affirm exercise benefits for anxiety and depression in cancer survivors, and prior research provides evidence at the weekly exercise-dose level (68), but the present dose–response findings should be interpreted cautiously. Although the spline models identified apparent peaks, these should not be interpreted as precise optima. For depression, the greatest estimated benefit was observed around 2.8 METs, together with 40 minutes per session and six sessions per week; however, this value is better interpreted as a pooled reference point within a lower-intensity to low-moderate range than as a fixed physiological optimum, especially because it was derived from a heterogeneous network and may still represent a meaningful relative stimulus in cancer survivors with low fitness, older age, treatment-related fatigue, or deconditioning. In addition, the dose variables were partly estimated retrospectively from intervention descriptions, data were sparse at some dose levels, and residual measurement heterogeneity across psychological scales may also have influenced the pooled estimates. Accordingly, the identified dose peaks are better viewed as overall reference points for hypothesis generation rather than definitive prescription targets.

### 4.3 Clinical implications

Structured exercise may be considered an adjunctive non-pharmacological strategy for ambulatory cancer survivors who are medically cleared for exercise. Given the comparatively more stable evidence base in the present network, Tai Chi and Qigong may be potentially useful mind-body options, while aerobic exercise may be implemented through accessible activities such as walking or cycling. For aerobic exercise, a reasonable starting format may involve moderate-intensity training performed three times per week for at least 12 weeks, with sessions of approximately 30 to 60 minutes and progression according to symptoms and tolerance. In the present pooled dose-response analysis, benefits for depression appeared to be relatively favorable at around 40 minutes per session, six sessions per week, and an intervention period approaching 20 weeks, whereas for anxiety, moderate-duration exercise performed about five times per week over a similar period may be associated with relatively stable benefits. These exercise modalities and dose ranges should therefore be interpreted as overall reference ranges for cancer survivors who are able to exercise, rather than as universal recommendations for all clinical stages or conditions.

### 4.4 Strengths and limitations

This study has several strengths: First, it employs an arm-level Bayesian multi-arm network meta-analysis that integrates multi-arm trials, diverse follow-up time points, and multiscale effects within a unified framework. Partial pooling explicitly addresses intra-study dependencies and inter-study heterogeneity, which may improve robustness relative to traditional meta-synthesis. Second, we harmonized heterogeneous scales by converting continuous outcomes to a common standardized metric (Hedges’ g), enhancing comparability across trials and streamlining data extraction. Third, it models dose using intensity, frequency, and duration, provides relative rankings across modalities, and identifies outcome-specific optimal dose ranges for depression and anxiety, thereby improving prescription feasibility and clinical applicability.

However, this study has several limitations. Some trials had small sample sizes, and the asymmetric funnel plots suggest possible publication bias. Limited reporting of adherence and intervention fidelity may also have affected the estimated effects. In addition, some dose variables were estimated retrospectively from intervention descriptions rather than directly measured. Although exercise intensity, session duration, and weekly frequency were coded continuously for the dose–response analyses, aerobic + resistance training remained a broad category in the network comparisons; therefore, intensity-specific differences within this modality could not be interpreted directly from the categorical network estimates. Different validated psychological scales were harmonized using standardized mean differences and modeled within a multilevel framework, but residual scale-related heterogeneity may still have influenced the pooled estimates. Sensitivity analyses using alternative assumed correlation coefficients nevertheless yielded broadly consistent results, suggesting that the main conclusions were not materially dependent on this assumption. Although Pilates ranked highly for some outcomes, this finding remains preliminary because the available trials were few and small. Moreover, although interaction terms were explored in the dose–response models, we did not prespecify or separately report a specific interaction between intervention duration and exercise intensity; therefore, the relative comparability of long-duration low-intensity versus short-duration high-intensity protocols remains uncertain. The wider credible intervals in the subgroup without concurrent radiotherapy or chemotherapy likely reflect the smaller sample size and should be interpreted cautiously. Finally, because the dose–response curves were estimated across the overall network rather than separately by treatment phase, these dose parameters should be viewed as overall reference ranges rather than phase-specific prescriptions. Concurrent radiotherapy or chemotherapy status is only an imperfect proxy for clinical phase, and the included evidence did not represent all cancer types evenly; therefore, the findings may not generalize to palliative or end-of-life populations, patients with poor performance status or marked functional decline, or underrepresented cancer populations.

## 5 Conclusion

This study suggests that structured exercise may help alleviate depression and anxiety symptoms in cancer survivors. Mind-body exercise and aerobic exercise appeared relatively favorable in the current network, although certainty was low to very low for several comparisons. For depressive symptoms, the pooled dose-response model suggested a relatively favorable reference point around 2.8 METs, 40 minutes per session, and six sessions per week, but this estimate should be interpreted cautiously and individualized rather than applied as a fixed prescription. For anxiety, moderate session duration, about five sessions per week, and an intervention period of approximately 20 weeks appeared to be associated with relatively stable benefits. These findings should be interpreted as pooled estimates derived from the cancer populations represented in the included trials and should not be extrapolated uncritically to underrepresented or unrepresented cancer types.

## Data Availability

The original contributions presented in the study are included in the article/**Supplementary Material**. Further inquiries can be directed to the corresponding author.

## References

[1] Santomauro DF Mantilla Herrera AM Shadid J Zheng P Ashbaugh C Pigott DM . Global prevalence and burden of depressive and anxiety disorders in 204 countries and territories in 2020 due to the COVID-19 pandemic. Lancet. (2021) 398:1700–12. doi: 10.1016/s0140-6736(21)02143-7. PMID: 34634250 PMC8500697

[2] Rong J Wang X Cheng P Li D Zhao D . Global, regional and national burden of depressive disorders and attributable risk factors, from 1990 to 2021: results from the 2021 Global Burden of Disease study. Br J Psychiatry. (2025) 227(4):688–97. doi: 10.1192/bjp.2024.266. PMID: 39809717

[3] Zhou J Li S Song Y Ying J Luo Z Shan S . Global, regional, and national trends in the burden of anxiety disorders from 1992 to 2021: An age–period–cohort analysis based on the Global Burden of Disease Study 2021. Depression Anxiety. (2025) 2025:4178541. doi: 10.1155/da/4178541. PMID: 40688542 PMC12276053

[4] Mehnert A Brähler E Faller H Härter M Keller M Schulz H . Four-week prevalence of mental disorders in patients with cancer across major tumor entities. J Clin Oncol. (2014) 32:3540–6. doi: 10.1200/jco.2014.56.0086. PMID: 25287821

[5] Krebber AMH Buffart LM Kleijn G Riepma IC de Bree R Leemans CR . Prevalence of depression in cancer patients: a meta‐analysis of diagnostic interviews and self‐report instruments. Psycho‐oncology. (2014) 23:121–30. doi: 10.1002/pon.3409. PMID: 24105788 PMC4282549

[6] Forbes H Carreira H Funston G Andresen K Bhatia U Strongman H . Early, medium and long-term mental health in cancer survivors compared with cancer-free comparators: matched cohort study using linked UK electronic health records. EClinicalMedicine. (2024) 76:102826. doi: 10.2139/ssrn.4788505. PMID: 39318789 PMC11421364

[7] Mitchell AJ Ferguson DW Gill J Paul J Symonds P . Depression and anxiety in long-term cancer survivors compared with spouses and healthy controls: a systematic review and meta-analysis. Lancet Oncol. (2013) 14:721–32. doi: 10.1016/s1470-2045(13)70244-4. PMID: 23759376

[8] Andersen BL Lacchetti C Ashing K Berek JS Berman BS Bolte S . Management of anxiety and depression in adult survivors of cancer: ASCO guideline update. J Clin Oncol. (2023) 41:3426–53. doi: 10.1200/jco.23.00293. PMID: 37075262

[9] Faller H Schuler M Richard M Heckl U Weis J Küffner R . Effects of psycho-oncologic interventions on emotional distress and quality of life in adult patients with cancer: systematic review and meta-analysis. J Clin Oncol. (2013) 31:782–93. doi: 10.1200/jco.2011.40.8922. PMID: 23319686

[10] Sharpe M Walker J Holm Hansen C Martin P Symeonides S Gourley C . Integrated collaborative care for comorbid major depression in patients with cancer (SMaRT Oncology-2): a multicentre randomised controlled effectiveness trial. Lancet. (2014) 384:1099–108. doi: 10.1016/s0140-6736(14)61231-9. PMID: 25175478

[11] Ostuzzi G Matcham F Dauchy S Barbui C Hotopf M . Antidepressants for the treatment of depression in people with cancer. Cochrane Database Syst Rev. (2018) 4(4):CD011006. doi: 10.1002/14651858.cd011006.pub3. PMID: 29683474 PMC6494588

[12] Australia COSo . COSA position statement on exercise in cancer care. Sydney: Clinical Oncology Society of Australia (2018).

[13] Tavares VDDO Cuthbert C Teychenne M Schuch FB Cabral D Menezes de Sousa G . The effects of exercise on anxiety and depression in adults with cancer: A meta-review of meta-analyses. J Psychosocial Oncol. (2025) 43:753–76. doi: 10.1080/07347332.2024.2441693. PMID: 39704272

[14] Ramírez-Vélez R Zambom-Ferraresi F García-Hermoso A Kievisiene J . Evidence-based exercise recommendations to improve mental wellbeing in women with breast cancer during active treatment: a systematic review and meta-analysis. Cancers. (2021) 13:264. doi: 10.3390/cancers13020264, PMID: 33445739 PMC7828164

[15] Zhao C Tao X Lei B Zhang Y Li G Lv Y . Effects of exercise on depression and anxiety in lung cancer survivors: A systematic review and meta-analysis of randomized controlled trials. Curr Oncol. (2025) 32:304. doi: 10.3390/curroncol32060304. PMID: 40558247 PMC12191834

[16] Cramer H Lauche R Klose P Lange S Langhorst J Dobos GJ . Yoga for improving health‐related quality of life, mental health and cancer‐related symptoms in women diagnosed with breast cancer. Cochrane Database Syst Rev. (2017) 1(1):CD010802. doi: 10.1002/14651858.cd010802. PMID: 28045199 PMC6465041

[17] Wayne PM Lee MS Novakowski J Osypiuk K Ligibel J Carlson LE . Tai Chi and Qigong for cancer-related symptoms and quality of life: a systematic review and meta-analysis. J Cancer Survivorship. (2018) 12:256–67. doi: 10.1007/s11764-017-0665-5. PMID: 29222705 PMC5958892

[18] Agudelo LZ Femenía T Orhan F Porsmyr-Palmertz M Goiny M . Skeletal muscle PGC-1α1 modulates kynurenine metabolism and mediates resilience to stress-induced depression. Cell. (2014) 159:33–45. doi: 10.1016/j.cell.2014.07.051. PMID: 25259918

[19] Yao M Qu Y Zheng Y Guo H . The effect of exercise on depression and gut microbiota: Possible mechanisms. Brain Res Bull. (2025) 220:111130. doi: 10.1016/j.brainresbull.2024.111130. PMID: 39557221

[20] Zhang Y Li G Zhang S Zhou Y Lv Y Feng L . Effects of exercise on depression and anxiety in breast cancer survivors: A systematic review and meta‐analysis of randomized controlled trials. Cancer Med. (2025) 14:e70671. doi: 10.1002/cam4.70671. PMID: 40052614 PMC11886893

[21] Yang L Courneya KS Friedenreich CM . The Physical Activity and Cancer Control (PACC) framework: update on the evidence, guidelines, and future research priorities. Br J Cancer. (2024) 131:957–69. doi: 10.1038/s41416-024-02748-x. PMID: 38926526 PMC11405831

[22] Li L Chen X Yang C Hui Z Fan T . Comparison of the efficacy of exercise interventions on depressive and anxiety symptoms in cancer patients: a systematic review and network meta-analysis. Neurosci Biobehav Rev. (2025) 176:106304. doi: 10.1016/j.neubiorev.2025.106304. PMID: 40721199

[23] Zhang H Hu Z Tong J Hu J Wang X Wang B . Comparative impact of exercise variants on depressive symptoms in cancer patients: A systematic review and network meta-analysis. Eur J Oncol Nurs. (2025) 74:102773. doi: 10.1016/j.ejon.2024.102773. PMID: 39793191

[24] Fairman CM Nilsen TS Newton RU Taaffe DR Spry N Joseph D . Reporting of resistance training dose, adherence, and tolerance in exercise oncology. Med Sci Sports Exerc. (2020) 52(2):315–22. doi: 10.1249/MSS.0000000000002127, PMID: 31436734 PMC12376820

[25] Hoffmann TC Glasziou PP Boutron I Milne R Perera R Moher D . Better reporting of interventions: template for intervention description and replication (TIDieR) checklist and guide. BMJ. (2014) 348:g1687. doi: 10.1136/bmj.g1687. PMID: 24609605

[26] Page MJ McKenzie JE Bossuyt PM . The PRISMA 2020 statement: an updated guideline for reporting systematic reviews. BMJ. (2021) 372:n71. doi: 10.31222/osf.io/v7gm2. PMID: 33782057 PMC8005924

[27] Abraha I Montedori A . Modified intention to treat reporting in randomised controlled trials: systematic review. BMJ. (2010) 340:c2697. doi: 10.1136/bmj.c2697. PMID: 20547685 PMC2885592

[28] Denlinger CS Carlson RW Are M Baker KS Davis E Edge SB . Survivorship: introduction and definition. J Natl Compr Cancer Netw. (2014) 12:34–45. doi: 10.6004/jnccn.2014.0005. PMID: 24453291 PMC4465253

[29] Ashok P Kharche JS Raju R Godbole G . Metabolic equivalent task assessment for physical activity in medical students. Natl J Physiol Pharm Pharmacol. (2017) 7:236. doi: 10.5455/njppp.2017.7.0825604092016. PMID: 36448968

[30] Herrmann SD Willis EA Ainsworth BE Barreira TV Hastert M Kracht CL . 2024 Adult Compendium of Physical Activities: A third update of the energy costs of human activities. J Sport Health Sci. (2024) 13:6–12. doi: 10.1016/j.jshs.2023.10.010. PMID: 38242596 PMC10818145

[31] Glass S Dwyer GB Medicine ACoS . ACSM's metabolic calculations handbook. Baltimore, MD, USA: Lippincott Williams & Wilkins (2007).

[32] Sterne JAC Savović J Page MJ Elbers RG Blencowe NS Boutron I . RoB 2: a revised tool for assessing risk of bias in randomised trials. BMJ. (2019) 366:l4898. doi: 10.1136/bmj.l4898. PMID: 31462531

[33] Guyatt G Oxman AD Akl EA Kunz R Vist G Brozek J . GRADE guidelines: 1. Introduction—GRADE evidence profiles and summary of findings tables. J Clin Epidemiol. (2011) 64:383–94. doi: 10.1016/j.jclinepi.2010.04.026. PMID: 21195583

[34] Lüdecke D . esc: Effect size computation for meta-analysis (Version 0.5. 1) [R package]. Vienna, Austria: R Foundation for Statistical Computing / CRAN (2019).

[35] Hedges LV . Distribution theory for Glass's estimator of effect size and related estimators. J Educ Stat. (1981) 6:107–35. doi: 10.3102/10769986006002107. PMID: 38293548

[36] Hedges LV Olkin I . Statistical methods for meta-analysis. Orlando, FL, USA: Academic Press (2014).

[37] Williams DR Rast P Bürkner P-C . Bayesian meta-analysis with weakly informative prior distributions. Charlottesville, VA, USA: PsyArXiv / Center for Open Science (2018). doi: 10.31234/osf.io/7tbrm.

[38] Betancourt M Girolami M . Hamiltonian Monte Carlo for hierarchical models. Curr Trends Bayesian Method Appl. (2015) 79:2–4. doi: 10.1201/b18502-5

[39] Brooks SP Gelman A . General methods for monitoring convergence of iterative simulations. J Comput Graphical Stat. (1998) 7:434–9. doi: 10.1080/10618600.1998.10474787. PMID: 41909888

[40] Salanti G Ades A Ioannidis JP . Graphical methods and numerical summaries for presenting results from multiple-treatment meta-analysis: an overview and tutorial. J Clin Epidemiol. (2011) 64:163–71. doi: 10.1016/j.jclinepi.2010.03.016. PMID: 20688472

[41] Dias S Welton NJ Caldwell DM Ades AE . Checking consistency in mixed treatment comparison meta‐analysis. Stat Med. (2010) 29:932–44. doi: 10.1002/sim.3767. PMID: 20213715

[42] Higgins JPT Jackson D Barrett JK Lu G Ades AE White IR . Consistency and inconsistency in network meta‐analysis: concepts and models for multi‐arm studies. Res Synth Methods. (2012) 3:98–110. doi: 10.1002/jrsm.1044. PMID: 26062084 PMC4433772

[43] Gasparrini A Armstrong B Kenward MG . Multivariate meta‐analysis for non‐linear and other multi‐parameter associations. Stat Med. (2012) 31:3821–39. doi: 10.1002/sim.5471. PMID: 22807043 PMC3546395

[44] Carlson LE Ismaila N Addington EL Asher GN Atreya C Balneaves LG . Integrative oncology care of symptoms of anxiety and depression in adults with cancer: Society for Integrative Oncology–ASCO Guideline. J Clin Oncol. (2023) 41:4562–91. doi: 10.1200/jco.23.00857. PMID: 37582238

[45] Campbell KL Winters-Stone KM Wiskemann J May AM Schwartz AL . Exercise guidelines for cancer survivors: consensus statement from international multidisciplinary roundtable. Med Sci Sports Exercise. (2019) 51:2375. doi: 10.1249/mss.0000000000002116. PMID: 31626055 PMC8576825

[46] Xu J Li H Sze D . Effectiveness of qigong and Tai Chi for quality of life in patients with cancer: an umbrella review and meta-analysis. BMC Complementary Med Ther. (2025) 25:141. doi: 10.1186/s12906-025-04875-1. PMID: 40240905 PMC12004731

[47] Sun F Li L Wen X Xue Y Yin J . The effect of Tai Chi/Qigong on depression and anxiety symptoms in adults with cancer: A systematic review and meta-regression. Complementary Ther Clin Pract. (2024) 56:101850. doi: 10.1016/j.ctcp.2024.101850. PMID: 38626582

[48] Larkey LK Roe DJ Weihs KL Jahnke R Lopez AM Rogers CE . Randomized controlled trial of Qigong/Tai Chi Easy on cancer-related fatigue in breast cancer survivors. Ann Behav Med. (2015) 49:165–76. doi: 10.1007/s12160-014-9645-4. PMID: 25124456 PMC4329282

[49] Bower JE Irwin MR . Mind–body therapies and control of inflammatory biology: A descriptive review. Brain Behav Immun. (2016) 51:1–11. doi: 10.1016/j.bbi.2015.06.012. PMID: 26116436 PMC4679419

[50] Zhou Y Wang Q Larkey L James D Cui H . Tai Chi effects on heart rate variability: a systematic review and meta-analysis. J Integr Complementary Med. (2024) 30:121–32. doi: 10.1089/jicm.2022.0682. PMID: 37695835

[51] Eyigor S Karapolat H Yesil H Uslu R Durmaz B . Effects of pilates exercises on functional capacity, flexibility, fatigue, depression and quality of life in female breast cancer patients: a randomized controlled study. Eur J Phys Rehabil Med. (2010) 46:481–7. doi: 10.4172/1948-5956.10000s13. PMID: 21224783

[52] Espíndula RC Nadas GB da Rosa MI Foster C de Araújo FC Grande AJ . Pilates for breast cancer: a systematic review and meta-analysis. Rev da Associação Médica Bras. (2017) 63:1006–12. doi: 10.1590/1806-9282.63.11.1006, PMID: 29451666

[53] Razak NA Azhar ZI Ismail Z Mohd Azman ZA Abdul Manap SA Ramli N . Impact of pilates exercise on quality of life, functional capacity, cancer-related fatigue, depression and salivary cortisol of colorectal cancer survivors: a quasi-experimental study. Asian Pac J Cancer Prev: APJCP. (2024) 25:2895. doi: 10.31557/apjcp.2024.25.8.2895. PMID: 39205588 PMC11495429

[54] Zaccaro A Piarulli A Laurino M Garbella E Menicucci D Neri B . How breath-control can change your life: a systematic review on psycho-physiological correlates of slow breathing. Front Hum Neurosci. (2018) 12:353. doi: 10.3389/fnhum.2018.00353. PMID: 30245619 PMC6137615

[55] Laborde S Allen MS Borges U Dosseville F Hosang T Iskra M . Effects of voluntary slow breathing on heart rate and heart rate variability: a systematic review and a meta-analysis. Neurosci Biobehav Rev. (2022) 138:104711. doi: 10.1016/j.neubiorev.2022.104711. PMID: 35623448

[56] Russo MA Santarelli DM O’Rourke D . The physiological effects of slow breathing in the healthy human. Breathe. (2017) 13:298–309. doi: 10.1183/20734735.009817. PMID: 29209423 PMC5709795

[57] Giacomini MB da Silva AMV Weber LM Monteiro MB . The Pilates Method increases respiratory muscle strength and performance as well as abdominal muscle thickness. J Bodywork Mov Ther. (2016) 20:258–64. doi: 10.1016/j.jbmt.2015.11.003. PMID: 27210841

[58] Brown JC Huedo-Medina TB Pescatello LS Ryan SM Pescatello SM Moker E . The efficacy of exercise in reducing depressive symptoms among cancer survivors: a meta-analysis. PloS One. (2012) 7:e30955. doi: 10.1371/journal.pone.0030955. PMID: 22303474 PMC3267760

[59] Kulchycki M Halder HR Askin N Rabbani R Schulte F Jeyaraman MM . Aerobic physical activity and depression among patients with cancer: a systematic review and meta-analysis. JAMA Netw Open. (2024) 7:e2437964-e2437964. doi: 10.1001/jamanetworkopen.2024.37964. PMID: 39378035 PMC11581595

[60] Kang D-W Fairey AS Boulé NG Field CJ Wharton SA Courneya KS . A randomized trial of the effects of exercise on anxiety, fear of cancer progression and quality of life in prostate cancer patients on active surveillance. J Urol. (2022) 207:814–22. doi: 10.1097/ju.0000000000002334. PMID: 35179044

[61] Herranz-Gómez A Cuenca-Martínez F Suso-Martí L Varangot-Reille C . Effectiveness of HIIT in patients with cancer or cancer survivors: an umbrella and mapping review with meta‐meta‐analysis. Scand J Med Sci Sports. (2022) 32:1522–49. doi: 10.1111/sms.14223, PMID: 35925829 PMC9804206

[62] Wrann CD White JP Salogiannnis J Laznik-Bogoslavski D Wu J Ma D . Exercise induces hippocampal BDNF through a PGC-1α/FNDC5 pathway. Cell Metab. (2013) 18:649–59. doi: 10.1016/j.cmet.2013.09.008. PMID: 24120943 PMC3980968

[63] El Hayek L Khalifeh M Zibara V Abi Assaad R Emmanuel N Karnib N . Lactate mediates the effects of exercise on learning and memory through SIRT1-dependent activation of hippocampal brain-derived neurotrophic factor (BDNF). J Neurosci. (2019) 39:2369–82. doi: 10.1523/jneurosci.1661-18.2019. PMID: 30692222 PMC6435829

[64] Kiecolt-Glaser JK Derry HM Fagundes CP . Inflammation: depression fans the flames and feasts on the heat. Am J Psychiatry. (2015) 172:1075–91. doi: 10.1176/appi.ajp.2015.15020152. PMID: 26357876 PMC6511978

[65] Sinha MK Vaishali K Maiya AG Shivashankar KN Shashikiran U Ravi Shankar N . Association of physical activity and heart rate variability in people with overweight and obesity: a systematic review. F1000Research. (2023) 12:156. doi: 10.12688/f1000research.124707.1. PMID: 36875496 PMC9982191

[66] Soong RY Low CE Ong V Sim I Lee C Lee F . Exercise interventions for depression, anxiety, and quality of life in older adults with cancer: a systematic review and meta-analysis. JAMA Netw Open. (2025) 8:e2457859-e2457859. doi: 10.1001/jamanetworkopen.2024.57859. PMID: 39903465 PMC11795328

[67] Kenkhuis M-F Doorenbos M Mast IH Aaronson NK van Beurden M Bohus M . Exercise effects on symptoms of depression and anxiety vary by patient, clinical, and intervention characteristics in cancer survivors: results from pooled analyses of individual participant data of 26 RCTs. Supportive Care Cancer. (2025) 33:1–17. doi: 10.1007/s00520-025-09646-9. PMID: 40591016 PMC12213934

[68] Carayol M Bernard P Boiché J Riou F Mercier B Cousson-Gélie F . Psychological effect of exercise in women with breast cancer receiving adjuvant therapy: what is the optimal dose needed? Ann Oncol. (2013) 24:291–300. doi: 10.1093/annonc/mds342. PMID: 23041586

