# Supplementary material for: Effects of different exercise interventions on depression and anxiety in cancer survivors: a systematic review and Bayesian network meta-analysis of randomized controlled trials
Source: Front Oncol. 2026 Apr 20;16:1741755. doi: 10.3389/fonc.2026.1741755 (PMC13135953; doi:10.3389/fonc.2026.1741755)
Supplement: Supplementary file 1 [file DataSheet1.docx]

**Supplementary Material**

Effects of Different Exercise Interventions on Depression and Anxiety in Cancer Survivors: A Systematic Review and Bayesian Network Meta-Analysis of Randomized Controlled Trials

| **Supplementary 1: Search Strategy** |
| --- |
| **Supplementary 2: Characteristics of Included Studies** |
| **Supplementary 3: Sample of arm-based forest plot** (**Depression)** |
| **Supplementary 4: Sample of arm-based forest plot (Anxiety)** |
| **Supplementary 5: Risk of Bias in Individual Studies** |
| **Supplementary 6: Global and Local Inconsistency Evaluated (Depression)** |
| **Supplementary 7: Funnel plot (Depression)** |
| **Supplementary 8: Global and Local Inconsistency Evaluated (Anxiety)** |
| **Supplementary 9: Funnel plot (Anxiety)** |
| **Supplementary 10:Subgroup analysis** |
| **Supplementary 11:Sensitivity analyses** |
| **Supplementary 12: Dose-response relationship (Depression)** |
| **Supplementary 13: Model fitting effect (Depression)** |
| **Supplementary 14: Dose-response relationship (Anxiety)** |
| **Supplementary 15: Model fitting effect (Anxiety)** |
| **Supplementary 16: GRADE Summary** |

**Supplementary 1: Search Strategy**

**1.1 pubmed**

| **Search** | **Query** |
| --- | --- |
| #1 | ("Neoplasms"[Mesh] OR neoplasm*[Tiab] OR cancer*[Tiab] OR carcinoma*[Tiab] OR tumor*[Tiab] OR tumour*[Tiab] OR malignan*[Tiab] OR oncology[Tiab] OR "cancer survivor*"[Tiab]) |
| #2 | ("Exercise"[Mesh] OR "Exercise Therapy"[Mesh] OR "Physical Activity"[Mesh] OR exercis*[Tiab] OR "physical activit*"[Tiab] OR "physical training"[Tiab] OR "aerobic exercise"[Tiab] OR "resistance training"[Tiab] OR "strength training"[Tiab] OR "endurance training"[Tiab] OR "high intensity interval training"[Tiab] OR HIIT[Tiab] OR yoga[Tiab] OR "tai chi"[Tiab] OR pilates[Tiab] OR "mind-body exercise"[Tiab]) |
| #3 | ("Depression"[Mesh] OR "Depressive Disorder"[Mesh] OR "Anxiety"[Mesh] OR "Anxiety Disorders"[Mesh] OR depress*[Tiab] OR anxi*[Tiab] OR anxious[Tiab]) |
| #4 | ("Randomized Controlled Trial"[Publication Type] OR "Controlled Clinical Trial"[Publication Type] OR randomized[Tiab] OR randomised[Tiab] OR randomly[Tiab] OR trial[Tiab]) |
| #5 | #1 AND #2 AND #3 AND #4 |

**1.2 Web Of Science**

| **Search** | **Query** |
| --- | --- |
| #1 | (TS=(neoplasm* OR cancer* OR carcinoma* OR tumor* OR tumour* OR malignan* OR oncolog* OR "cancer survivor*")) AND (TS=( exercise* OR "physical activit*" OR "physical training" OR "exercise therap*" OR "aerobic exercise*" OR "cardiorespiratory training" OR "resistance training" OR "strength training" OR "endurance training" OR "high intensity interval training" OR HIIT OR yoga OR "tai chi" OR pilates OR "mind-body exercise*")) AND (TS=(depress* OR "major depress*" OR "depressive symptom*" OR anxi* OR anxious OR "anxiety disorder*")) AND (TS=(("randomized controlled" NEAR/2 trial*) OR ("controlled clinical" NEAR/2 trial*) OR (random* NEAR/2 (trial* OR study OR controlled)) OR "RCT")) |

**1.3 Cochrane library**

| **Search** | **Query** |
| --- | --- |
| #1 | MeSH descriptor: [Neoplasms] explode all trees |
| #2 | (‘neoplasm’ OR ‘cancer’ OR ‘carcinoma’ OR ‘tumor’ OR ‘tumour’ OR ‘malignan’ OR ‘oncolog’ OR ‘cancer survivor’):ti,ab,kw |
| #3 | MeSH descriptor: [Exercise Therapy] explode all trees |
| #4 | MeSH descriptor: [Exercise] explode all trees |
| #5 | ('exercise' OR 'physical activit' OR 'physical training' OR 'aerobic exercise*' OR 'resistance training' OR 'strength training' OR 'endurance training' OR 'high intensity interval training' OR 'HIIT' OR 'yoga' OR 'tai chi' OR 'pilates' OR 'mind-body exercise'):ti,ab,kw |
| #6 | MeSH descriptor: [Depression] explode all trees |
| #7 | MeSH descriptor: [Anxiety] explode all trees |
| #8 | (‘depress’ OR ‘major depress’ OR ‘depressive symptom’ OR ‘anxi’ OR ‘anxious’ OR ‘anxiety disorder’):ti,ab,kw |
| #9 | (‘randomi’ OR ‘randomly’ OR ‘trial’):ti,ab,kw |
| #10 | #1 OR #2 |
| #11 | #3 OR #4 OR #5 |
| #12 | #6 OR #7 OR #8 |
| #13 | #9 AND #10 AND #11 AND #12 |

**1.4 Embase**

| **Search** | **Query** |
| --- | --- |
| #1 | 'neoplasm'/exp |
| #2 | neoplasm*:ti,ab,kw OR cancer*:ti,ab,kw OR carcinoma*:ti,ab,kw OR tumor*:ti,ab,kw OR tumour*:ti,ab,kw OR malignan*:ti,ab,kw OR oncolog*:ti,ab,kw OR 'cancer survivor*':ti,ab,kw |
| #3 | 'exercise'/exp OR 'exercise therapy'/exp OR 'physical activity'/exp OR 'resistance training'/exp OR 'aerobic exercise'/exp OR 'high intensity interval training'/exp OR 'yoga'/exp OR 'tai ji'/exp OR 'pilates-based exercise' |
| #4 | exercise*:ti,ab,kw OR 'physical activit*':ti,ab,kw OR 'physical training':ti,ab,kw OR 'exercise therap*':ti,ab,kw OR 'aerobic exercise*':ti,ab,kw OR  'cardiorespiratory training':ti,ab,kw OR 'resistance training':ti,ab,kw OR 'strength training':ti,ab,kw OR 'endurance training':ti,ab,kw OR 'high intensity interval training':ti,ab,kw OR hiit:ti,ab,kw OR yoga:ti,ab,kw OR 'tai chi':ti,ab,kw OR pilates:ti,ab,kw OR 'mind-body exercise*':ti,ab,kw |
| #5 | 'depression'/exp OR 'depressive disorder'/exp OR 'anxiety'/exp OR 'anxiety disorder'/exp |
| #6 | depress*:ti,ab,kw OR 'major depress*':ti,ab,kw OR 'depressive symptom*':ti,ab,kw OR anxi*:ti,ab,kw OR anxious:ti,ab,kw OR 'anxiety disorder*':ti,ab,kw |
| #7 | 'randomized controlled trial'/exp OR 'controlled clinical trial'/exp |
| #8 | randomi*:ti,ab,kw OR randomly:ti,ab,kw OR trial:ti,ab,kw OR rct:ti,ab,kw OR (('randomized controlled' NEAR/2 trial*):ti,ab,kw) OR (('controlled clinical' NEAR/2 trial*):ti,ab,kw) |
| #9 | #1 OR #2 |
| #10 | #3 OR #4 |
| #11 | #5 OR #6 |
| #12 | #7 OR #8 |
| #13 | #9 AND #10 AND #11 AND #12 |

**Supplementary** **2****: Characteristics of Included Studies**

| Country | | Cance type | Stage | n | % Female | Mean Age (SD/Range) | Radiotherapy/Chemotherapy | sessions | Supervise | Treatment | weeks | Session duration | Intensity prescribed (METs/min) | Mean BMI(SD/Range) | Timepoints available | Outcomes measured |
| --- | --- | --- | --- | --- | --- | --- | --- | --- | --- | --- | --- | --- | --- | --- | --- | --- |
| (Díaz-Balboa, et al. 2024) | | |  |  |  |  |  |  |  |  |  |  |  |  |  |  |
| Spain | Breast Cancer | | I-III | 60 | 1.00 | 48.82 (8.02) | Y | 2 | Y | Aerobic+Resistance | 24 | 60 | 4.5 | 26.36 (5.77) | 2weeks | HADS-A, HADS-D |
| Spain | Breast Cancer | | I-III | 62 | 1.00 | 48.92 (8.51) | Y | 0 | NA | Control | 0 | 0 | 0 | 26.86 (5.31) | 2weeks | HADS-A, HADS-D |
| (Molassiotis, et al. 2021) | | |  |  |  |  |  |  |  |  |  |  |  |  |  |  |
| Vietnam | Lung Cancer | | I-IV | 78 | 0.24 | 57.62 (9.63) | Y | 4 | Y | TaiChi/Qigong | 6 | 60 | 3 | NA | Post,6weeks | DASS21-A |
| Vietnam | Mixed | | I-IV | 78 | 0.27 | 56.06 (9.25) | Y | 0 | NA | Control | 0 | 0 | 0 | NA | Post,6weeks | DASS21-A |
| (Lu, et al. 2024) | | |  |  |  |  |  |  |  |  |  |  |  |  |  |  |
| China | Lung Cancer | | 0-III | 28 | 0.47 | 56.67 (8.21) | N | 14 | Y | Yoga | 2 | 20 | 2.22 | 22.16 (1.87) | Post | HADS-D |
| China | Lung Cancer | | 0-III | 28 | 0.62 | 58.38 (7.40) | N | 0 | NA | Control | 0 | 0 | 0 | 22.84 (2.04) | Post | HADS-D |
| (Monga, et al. 2007) | | |  |  |  |  |  |  |  |  |  |  |  |  |  |  |
| United States | Prostate Cancer | | NA | 11 | 0 | 68 (4.20) | Y | 3 | Y | Aerobic | 8 | 45 | 5 | NA | Post | BDI |
| United States | Prostate Cancer | | NA | 10 | 0 | 70.6 (5.30) | Y | 0 | NA | Control | 0 | 0 | 0 | NA | Post | BDI |
| (Mariano, et al. 2015) | | |  |  |  |  |  |  |  |  |  |  |  |  |  |  |
| Brazil | Breast Cancer | | I-II | 6 | 1.00 | 56.16 (3.53) | Y | 7 | Y | Resistance | 3 | 30 | 3 | NA | Post | BDI |
| Brazil | Breast Cancer | | I-II | 7 | 1.00 | 54.50 (4.24) | Y | 0 | NA | Control | 0 | 0 | 0 | NA | Post | BDI |
| (Egegaard, et al. 2019) | | |  |  |  |  |  |  |  |  |  |  |  |  |  |  |
| Denmark | Lung Cancer | | III-IV | 8 | 0.63 | 64 (5.8) | Y | 5 | Y | Resistance | 7 | 20 | 2.6 | 24.1 (4.4) | Post | HADS-A, HADS-D |
| Denmark | Lung Cancer | | III-IV | 7 | 0.71 | 65 (4.7) | Y | 0 | NA | Control | 0 | 0 | 0 | 24.2 (1.9) | Post | HADS-A, HADS-D |
| (Pieczyńska, et al. 2023) | | |  |  |  |  |  |  |  |  |  |  |  |  |  |  |
| Poland | Malignant Brain Tumor | | III-IV | 19 | 0.18 | 45.59 (11.15) | Y | 5 | Y | Mixed | 4 | 60 | 3.83 | NA | Post,12weks | HADS-A, HADS-D |
| Poland | Malignant Brain Tumor | | III-IV | 14 | 0.44 | 60 (13.55) | Y | 0 | NA | Control | 0 | 0 | 0 | NA | Post,12weks | HADS-A, HADS-D |
| (Odynets, et al. 2019) | | |  |  |  |  |  |  |  |  |  |  |  |  |  |  |
| Ukraine | Breast Cancer | | I-II | 45 | 1.00 | 58.84 (1.36) | Y | 3 | Y | Aerobic+Resistance | 52 | 60 | 4.6 | 24.12 (0.24) | Post | HADS-A, HADS-D |
| Ukraine | Breast Cancer | | I-II | 40 | 1.00 | 59.40 (1.24) | Y | 3 | Y | Pilates | 52 | 50 | 3.15 | 24.20 (0.44) | Post | HADS-A, HADS-D |
| Ukraine | Breast Cancer | | I-II | 30 | 1.00 | 59.10 (1.37) | Y | 3 | Y | Yoga | 52 | 60 | 2.5 | 24.11 (0.38) | Post | HADS-A, HADS-D |
| (Donnelly, et al. 2011a) | | |  |  |  |  |  |  |  |  |  |  |  |  |  |  |
| Britain | Mixed | | I-III | 16 | 1.00 | 53.5 (8.7) | Y | 5 | Y | Aerobic+Resistance | 12 | 30 | 3.25 | 29.6 (8.3) | Post, 12 weeks | BDI |
| Britain | Mixed | | I-III | 17 | 1.00 | 52.1 (11.8) | Y | 0 | NA | Control | 0 | 0 | 0 | 30 (7.8) | Post, 12 weeks | BDI |
| (Milbury, et al. 2019) | | |  |  |  |  |  |  |  |  |  |  |  |  |  |  |
| United States | Malignant Brain Tumor | | II-IV | 10 | 0.50 | 47.91 (14.66) | Y | 3 | Y | Yoga | 6 | 45 | 1.78 | NA | Post | CES-D |
| United States | Malignant Brain Tumor | | II-IV | 10 | 0.50 | 44.73 (12.23) | Y | 0 | NA | Control | 0 | 0 | 0 | NA | Post | CES-D |
| (Bower, et al. 2012) | | |  |  |  |  |  |  |  |  |  |  |  |  |  |  |
| United States | Breast Cancer | | 0-II | 16 | 1.00 | NA | Y | 2 | Y | Yoga | 12 | 90 | 2.5 | NA | Post,12weeks | BDI |
| United States | Breast Cancer | | 0-II | 15 | 1.00 | NA | Y | 0 | NA | Control | 0 | 0 | 0 | NA | Post,12weeks | BDI |
| (Chang, et al. 2008) | | |  |  |  |  |  |  |  |  |  |  |  |  |  |  |
| China Taiwan | Blood Cancer | | NA | 11 | 0.27 | 49.42 (15.3) | Y | 5 | Y | Aerobic | 3 | 12 | 3 | NA | Post | POMS-A, POMS-D |
| China Taiwan | Blood Cancer | | NA | 11 | 0.64 | 53.3 (13.6) | Y | 0 | NA | Control | 0 | 0 | 0 | NA | Post | POMS-A, POMS-D |
| (Midtgaard, et al. 2011) | | |  |  |  |  |  |  |  |  |  |  |  |  |  |  |
| Denmark | Mixed | | NA | 106 | 0.79 | 47 (10.8) | Y | 5 | Y | Mixed | 6 | 90 | 3.5 | NA | Post | HADS-A, HADS-D |
| Denmark | Mixed | | NA | 103 | 0.71 | 48 (10.1) | Y | 0 | NA | Control | 0 | 0 | 0 | NA | Post | HADS-A, HADS-D |
| (Kamen, et al. 2016) |  |  |  |  |  |  |  |  |  |  |  |  |  |  |  |  |
| United States | Mixed | | I-IV | 10 | NA | NA | Y | 7 | N | Aerobic+Resistance | 6 | 48 | 3.4 | NA | Post | STAI, CES-D |
| United States | Mixed | | I-IV | 12 | NA | NA | Y | 0 | NA | Control | 0 | 0 | 0 | NA | Post | STAI, CES-D |
| (Cantarero-Villanueva, et al. 2013) |  |  |  |  |  |  |  |  |  |  |  |  |  |  |  |  |
| Spain | Breast Cancer | | I-III | 32 | 1.00 | 49 (7) | Y | 3 | Y | Aerobic+Resistance | 8 | 60 | 5 | NA | Post,24weeks | POMS-A, POMS-D |
| Spain | Breast Cancer | | I-III | 29 | 1.00 | 47 (8) | Y | 0 | NA | Control | 0 | 0 | 0 | NA | Post,24weeks | POMS-A, POMS-D |
| (Wong, et al. 2024) |  |  |  |  |  |  |  |  |  |  |  |  |  |  |  |  |
| Hong Kong | Breast Cancer | | I-III | 16 | 1.00 | 48.63 (8.77) | Y | 1 | Y | Yoga | 8 | 60 | 3.50 | NA | Post,4weeks | HADS-A, HADS-D |
| Hong Kong | Breast Cancer | | I-III | 18 | 1.00 | 45.78 (9.25) | Y | 0 | NA | Control | 0 | 0 | 0 | NA | Post,4weeks | HADS-A, HADS-D |
| (Donnelly, et al. 2011b) |  |  |  |  |  |  |  |  |  |  |  |  |  |  |  |  |
| Britain | Gynecologic Cancers | | I-III | 16 | 1.00 | 53.5 (8.7) | Y | 5 | N | Aerobic+Resistance | 12 | 30 | 3.55 | 29.6 (8.3) | Post,12weeks | BDI |
| Britain | Gynecologic Cancers | | I-III | 17 | 1.00 | 52.1 (11.8) | Y | 0 | NA | Control | 0 | 0 | 0 | 30 (7.8) | Post,12weeks | BDI |
| (Courneya, et al. 2014) |  |  |  |  |  |  |  |  |  |  |  |  |  |  |  |  |
| Canada | Breast Cancer | | I-III | 95 | 1.00 | NA | Y | 3 | Y | Aerobic | 17 | 28 | 8 | NA | 4weeks | STAI, CES-D |
| Canada | Breast Cancer | | I-III | 99 | 1.00 | NA | Y | 3 | Y | Aerobic | 17 | 48 | 8 | NA | 4weeks | STAI, CES-D |
| Canada | Breast Cancer | | I-III | 102 | 1.00 | NA | Y | 3 | Y | Aerobic+Resistance | 17 | 55 | 6 | NA | 4weeks | STAI, CES-D |
| (Golsteijn, et al. 2018) |  |  |  |  |  |  |  |  |  |  |  |  |  |  |  |  |
| Netherlands | Mixed | | NA | 248 | 0.15 | 66.55 (7.07) | Y | 3 | N | Aerobic | 12 | 48 | 4.3 | 26.39 (3.38) | 12weeks,36weeks | HADS-A, HADS-D |
| Netherlands | Mixed | | NA | 227 | 0.11 | 66.38 (8.21) | Y |  | NA | Control | 0 | 0 | 0 | 26.74 (4.41) | 12weeks,36weeks | HADS-A, HADS-D |
| (Nicole Culos‐Reed, et al. 2006) |  |  |  |  |  |  |  |  |  |  |  |  |  |  |  |  |
| Canada | Breast Cancer | | NA | 20 | 1.00 | NA | NA | 3 | Y | Yoga | 7 | 75 | 2.57 | NA | post | POMS-A, POMS-D |
| Canada | Breast Cancer | | NA | 18 | 1.00 | NA | NA | 0 | NA | Control | 0 | 0 | 0 | NA | post | POMS-A, POMS-D |
| (Piraux, et al. 2022) |  |  |  |  |  |  |  |  |  |  |  |  |  |  |  |  |
| Belgium | Rectal Cancer | | II-III | 6 | 0.17 | 60.27  (8) | Y | 3 | Y | High-intensity interval | 5 | 33 | 5.79 | 27.8 (4.15) | post | CES-D |
| Belgium | Rectal Cancer | | II-III | 6 | 0.33 | 62.6 (15.33) | Y | 3 | Y | Resistance | 5 | 35 | 3.5 | 27.17 (4.22) | post | CES-D |
| Belgium | Rectal Cancer | | II-III | 6 | 0.33 | 66.5 (7.41) | Y | 0 | NA | Control | 0 | 0 | 0 | 25.63 (3,7) | post | CES-D |
| (Culos-Reed, et al. 2010) |  |  |  |  |  |  |  |  |  |  |  |  |  |  |  |  |
| Canada | Prostate Cancer | | NA | 37 | 0 | 67.2 (8.8) | N | 5 | Y | Mixed | 16 | 90 | 3.15 | 28.86 (2.15) | post | CES-D |
| Canada | Prostate Cancer | | NA | 24 | 0 | 68 (8.4) | N | 0 | NA | Control | 0 | 0 | 0 | 28.29 (4.48) | post | CES-D |
| (Rogers, et al. 2023) |  |  |  |  |  |  |  |  |  |  |  |  |  |  |  |  |
| United States | Breast Cancer | | I-III | 110 | 1.00 | NA | Y | 2 | Y | Aerobic | 12 | 48 | 3.1 | 30.8 (6.9) | Post,12weeks,36weeks | HADS-A, HADS-D |
| United States | Breast Cancer | | I-III | 112 | 1.00 | NA | Y | 0 | NA | Control | 0 | 0 | 0 | 30.6 (6.8) | Post,12weeks,36weeks | HADS-A, HADS-D |
| (Lanctôt, et al. 2016) |  |  |  |  |  |  |  |  |  |  |  |  |  |  |  |  |
| Canada | Breast Cancer | | I-III | 58 | 1.00 | 51.23 (9.29) | Y | 1 | Y | Yoga | 8 | 90 | 2.5 | NA | post | STAI,BDI |
| Canada | Breast Cancer | | I-III | 43 | 1.00 | 50.29 (9.54) | Y | 0 | NA | Control | 0 | 0 | 0 | NA | post | STAI,BDI |
| (Courneya, et al. 2007) |  |  |  |  |  |  |  |  |  |  |  |  |  |  |  |  |
| Canada | Breast Cancer | | I-III | 78 | 1.00 | 49 | Y | 3 | Y | Aerobic | 18 | 40 | 7.1 | 26.7 (5.6) | Post,24weeks | STAI,CES-D |
| Canada | Breast Cancer | | I-III | 82 | 1.00 | 49.5 | Y | 3 | Y | Resistance | 18 | 48 | 5 | 26.1 (5.5) | Post,24weeks | STAI,CES-D |
| Canada | Breast Cancer | | I-III | 82 | 1.00 | 49 | Y | 0 | NA | Control | 0 | 0 | 0 | 27.1 (5.4) | Post,24weeks | STAI,CES-D |
| (Choi and Kang 2012) |  |  |  |  |  |  |  |  |  |  |  |  |  |  |  |  |
| Korea | Gastric Cancer | | I-III | 11 | 0.55 | NA | Y | 3 | N | Aerobic | 8 | 60 | 3.86 | NA | post | STAI |
| Korea | Gastric Cancer | | I-III | 13 | 0.31 | NA | Y | 0 | NA | Control | 0 | 0 | 0 | NA | post | STAI |
| (Eyigor, et al. 2018) |  |  |  |  |  |  |  |  |  |  |  |  |  |  |  |  |
| Turkey | Breast Cancer | | NA | 22 | 1.00 | 52.3 (9.5) | Y | 2 | Y | Yoga | 10 | 60 | 2.5 | 26.4 (4.9) | post | BDI |
| Turkey | Breast Cancer | | NA | 20 | 1.00 | 51.5 (7.3) | Y | 0 | NA | Control | 0 | 0 | 0 | 25.5 (3.5) | post | BDI |
| (Cheng, et al. 2021) |  |  |  |  |  |  |  |  |  |  |  |  |  |  |  |  |
| china | Mixed | | NA | 27 | 0.52 | 67.8 (7.3) | Y | 3 | Y | TaiChi/Qigong | 12 | 40 | 2.85 | NA | post | GAD-7,PHQ-9 |
| china | Mixed | | NA | 25 | 0.36 | 65.2 (9.1) | Y | 3 | Y | Resistance | 12 | 40 | 5.08 | NA | post | GAD-7,PHQ-9 |
| china | Mixed | | NA | 27 | 0.44 | 66.3 (3.8) | Y | 3 | Y | Resistance | 12 | 40 | 3.2 | NA | post | GAD-7,PHQ-9 |
| china | Mixed | | NA | 26 | 0.38 | 64.6 (7.6) | Y | 0 | NA | Control | 0 | 0 | 0 | NA | post | GAD-7,PHQ-9 |
| (Ma, et al. 2025) |  | | | | | | | | | | | | | | | |
| china | Mixed | | I-IV | 12 | 1.00 | 43 (10.23) | Y | 2 | Y | Yoga | 6 | 70 | 2 | NA | post | HADS-A, HADS-D |
| china | Mixed | | I-IV | 12 | 1.00 | 44.25 (8.97) | Y | 0 | NA | Control | 0 | 0 | 0 | NA | post | HADS-A, HADS-D |
| (Yagli and Ulger 2015) |  | | | | | | | | | | | | | | | |
| Turkey | Breast Cancer | | I-II | 10 | 1.00 | 68.58 (6.17) | Y | 2 | Y | Yoga | 4 | 60 | 2 | 22.51 (2.11) | post | BDI |
| Turkey | Breast Cancer | | I-II | 10 | 1.00 | 68.88 (2.93) | Y | 2 | Y | Mixed | 4 | 60 | 3 | 22.09 (2.71) | post | BDI |
| (Galvão, et al. 2021) |  | | | | | | | | | | | | | | | |
| Australia | Prostate Cancer | | NA | 49 | 0 | 68.7 (9.3) | N | 4 | Y | Aerobic+Resistance | 48 | 48 | 7.5 | NA | post | BSI-A,BSI-D |
| Australia | Prostate Cancer | | NA | 50 | 0 | 69.1 (9.6) | N | 2 | Y | Aerobic+Resistance | 48 | 48 | 6.5 | NA | post | BSI-A,BSI-D |
| Australia | Prostate Cancer | | NA | 36 | 0 | 69.7 (8.4) | N | 0 | NA | Control | 0 | 0 | 0 | NA | post | BSI-A,BSI-D |
| (Piraux, et al. 2021) |  | | | | | | | | | | | | | | | |
| Belgium | Prostate Cancer | | NA | 24 | 0 | 67.4 (8.9) | Y | 3 | Y | High-intensity interval | 8 | 40 | 7.03 | 26.5 (3.9) | post | CES-D |
| Belgium | Prostate Cancer | | NA | 24 | 0 | 67.9 (7.1) | Y | 3 | Y | Resistance | 8 | 30 | 5 | 26.1 (2.9) | post | CES-D |
| Belgium | Prostate Cancer | | NA | 24 | 0 | 71.9 (8.1) | Y | 0 | NA | Control | 0 | 0 | 0 | 25.8 (4.4) | post | CES-D |
| (Cordier, et al. 2019) |  | | | | | | | | | | | | | | | |
| Switzerland | Malignant Brain Tumor | | III-IV | 10 | NA | 49.1 (13.14) | Y | 2 | Y | Aerobic | 6 | 45 | 5.08 | NA | post | STAI |
| Switzerland | Malignant Brain Tumor | | III-IV | 11 | NA | 54.6 (13.45) | Y | 2 | Y | Resistance | 6 | 45 | 3.40 | NA | post | STAI |
| Switzerland | Malignant Brain Tumor | | III-IV | 8 | NA | 53 (10.78) | Y | 0 | NA | Control | 0 | 0 | 0 | NA | post | STAI |
| (Dülger, et al. 2022) |  | | | | | | | | | | | | | | | |
| Turkey | Pituitary Neuroendocrine Tumor | | NA | 5 | 1.00 | 52 (13.5) | N | 3 | Y | Aerobic+Resistance | 6 | 60 | 3.9 | 26.1 (8.15) | post | HADS-A, HADS-D |
| Turkey | Pituitary Neuroendocrine Tumor | | NA | 5 | 1.00 | 41.8 (14) | N | 3 | NA | Yoga | 6 | 60 | 2.27 | 20.55 (2.93) | post | HADS-A, HADS-D |
| (Kim, et al. 2019) |  | | | | | | | | | | | | | | | |
| Korea | Mixed | | II-III | 37 | 0.51 | 55.7 (8.7) | N | 7 | Y | Aerobic+Resistance | 12 | 30 | 4.5 | 23.7 (2.9) | post | PHQ-9 |
| Korea | Mixed | | II-III | 34 | 0.50 | 56.8 (10.2) | N | 0 | NA | Control | 0 | 0 | 0 | 23.3 (3.6) | post | PHQ-9 |
| (Knoerl, et al. 2022b) |  | | | | | | | | | | | | | | | |
| United States | Breast Cancer | | I-III | 26 | 1.00 | 52.3 (9.6) | N | 2 | Y | Aerobic+Resistance | 4 | 75 | 5.05 | 30.7 (6.1) | Post,4weeks | HADS-A, HADS-D |
| United States | Breast Cancer | | I-III | 21 | 1.00 | 53.4 (8.0) | N | 0 | N | Control | 0 | 0 | 0 | 29.6 (7.1) | Post,4week | HADS-A, HADS-D |
| (Adams, et al. 2018) |  | | | | | | | | | | | | | | | |
| Canada | Testicular Cancer | | I-IV | 35 | 0 | 44 (11.6) | Y | 3 | Y | High-intensity interval | 12 | 35 | 7.05 | NA | Post,12week | STAI, CES-D |
| Canada | Testicular Cancer | | I-IV | 27 | 0 | 43.3 (9.9) | Y | 0 | NA | Control | 0 | 0 | 0 | NA | Post,12week | STAI, CES-D |
| (Ratcliff, et al. 2016) |  | | | | | | | | | | | | | | | |
| United States | Breast Cancer | | 0-III | 53 | 1.00 | 52.38 (9.83) | Y | 3 | Y | Yoga | 6 | 60 | 1.92 | NA | Post,4weeks,12week,24weeks | CES-D |
| United States | Breast Cancer | | 0-III | 53 | 1.00 | 52.11 (9.85) | Y | 0 | NA | Control | 0 | 0 | 0 | NA | Post,4weeks,12week,24weeks | CES-D |
| (Hardoerfer and Jentschke 2018) |  | | | | | | | | | | | | | | | |
| Germany | Mixed | | NA | 37 | 0.87 | 59 (12) | Y | 1 | Y | Yoga | 8 | 60 | 3.5 | NA | post | GAD-7, PHQ-2 |
| Germany | Mixed | | NA | 33 | 0.91 | 56 (14) | Y | 0 | NA | Control | 0 | 0 | 0 | NA | post | GAD-7, PHQ-2 |
| (Moraes, et al. 2021) |  | | | | | | | | | | | | | | | |
| Brazil | Breast Cancer | | NA | 12 | 1.00 | 55.0 (5.8) | Y | 1 | Y | Resistance | 8 | 48 | 4.5 | 28 (7.1) | post | STAI |
| Brazil | Breast Cancer | | NA | 13 | 1.00 | 54.3 (5.2) | Y | 0 | NA | Control | 0 | 0 | 0 | 26.8 (2.2) | post | STAI |
| (Aydin, et al. 2021) |  | | | | | | | | | | | | | | | |
| Turkey | Breast Cancer | | NA | 24 | 1.00 | 45 (2.2) | Y | 5 | Y | Aerobic+Resistance | 12 | 55 | 3.37 | NA | post | BDI |
| Turkey | Breast Cancer | | NA | 24 | 1.00 | 45 (2.2) | Y | 0 | NA | Control | 0 | 0 | 0 | NA | post | BDI |
| (He, et al. 2022) |  | | | | | | | | | | | | | | | |
| China | Breast Cancer | | I-III | 88 | 1.00 | 47.99 (8.62) | Y | 5 | Y | Aerobic | 8 | 30 | 4.8 | NA | 1weeks | PHQ-9 |
| China | Breast Cancer | | I-III | 88 | 1.00 | 48.32 (10) | Y | 0 | NA | Control | 0 | 0 | 0 | NA | 1weeks | PHQ-9 |
| (Rao, et al. 2017) |  | | | | | | | | | | | | | | | |
| India | Breast Cancer | | II-III | 33 | 1.00 | NA | Y | 6 | Y | Yoga | 12 | 60 | 1.66 | NA | post | STAI,BDI |
| India | Breast Cancer | | II-III | 36 | 1.00 | NA | Y | 0 | NA | Control | 0 | 0 | 0 | NA | post | STAI,BDI |
| (Danhauer, et al. 2009) |  | | | | | | | | | | | | | | | |
| United States | Breast Cancer | | I-IV | 22 | 1.00 | 54.3 (9.6) | Y | 1 | Y | Yoga | 10 | 75 | 2.8 | NA | post | CES-D |
| United States | Breast Cancer | | I-IV | 22 | 1.00 | 57.2 (10.2) | Y | 0 | NA | Control | 0 | 0 | 0 | NA | post | CES-D |
| (Rehman, et al. 2023) |  | | | | | | | | | | | | | | | |
| Pakistan | Mixed | | I-II | 20 | 0.45 | 48.1 (4.0) | Y | 5 | Y | Aerobic | 4 | 30 | 2.46 | 23.6 (1.6) | post | HADS-D |
| Pakistan | Mixed | | I-II | 20 | 0.35 | 48.3 (3.8) | Y | 0 | NA | Control | 0 | 0 | 0 | 22.5 (2.2) | post | HADS-D |
| (Zhang, et al. 2023) |  | | | | | | | | | | | | | | | |
| China | Breast Cancer | | I-IV | 90 | 1.00 | 48.58 (14.77) | Y | 7 | Y | Mixed | 5 | 60 | 2.76 | NA | post | HADS-A,HADS-D |
| China | Breast Cancer | | I-IV | 90 | 1.00 | 49.27 (10.33) | Y | 0 | NA | Control | 0 | 0 | 0 | NA | post | HADS-A,HADS-D |
| (Mehnert, et al. 2011) |  | | | | | | | | | | | | | | | |
| Germany | Breast Cancer | | I-III | 30 | 1.00 | 53.03 (7.4) | Y | 2 | Y | Mixed | 10 | 90 | 4.38 | NA | post | HADS-A,HADS-D |
| Germany | Breast Cancer | | I-III | 28 | 1.00 | 50.64 (9.44) | Y | 0 | NA | Control | 0 | 0 | 0 | NA | post | HADS-A,HADS-D |
| (Vargas-Román, et al. 2022) |  | | | | | | | | | | | | | | | |
| Spain | Blood Cancer | | I-IV | 20 | 0.50 | 45.20 (10.88) | Y | 2 | Y | TaiChi/Qigong | 8 | 60 | 2.22 | 23.10 (3.55) | post | HADS-A,HADS-D |
| Spain | Blood Cancer | | I-IV | 19 | 0.47 | 43.74 (10.53) | Y | 0 | NA | Control | 0 | 0 | 0 | 26.48 (4.23) | post | HADS-A,HADS-D |
| (Zetzl, et al. 2021) |  | | | | | | | | | | | | | | | |
| Germany | Mixed | | NA | 81 | 0.68 | 59.9 (11.7) | Y | 1 | Y | Yoga | 8 | 60 | 3.5 | NA | 2weeks | PHQ-9 |
| Germany | Mixed | | NA | 75 | 0.72 | 60.9 (10.9) | Y | 0 | NA | Control | 0 | 0 | 0 | NA | 2weeks | PHQ-9 |
| (Raghavendra, et al. 2009) |  | | | | | | | | | | | | | | | |
| India | Breast Cancer | | II-III | 42 | 1.00 | 46 (9.13) | y | 3 | y | Yoga | 6 | 60 | 1.58 | NA | post | HADS-A,HADS-D |
| India | Breast Cancer | | II-III | 33 | 1.00 | 48.45 (10.21) | y | 0 | NA | Control | 0 | 0 | 0 | NA | post | HADS-A,HADS-D |
| (Eyigor, et al. 2010) |  | | | | | | | | | | | | | | | |
| Turkey | Breast Cancer | | NA | 27 | 1.00 | 48.52 (7.62) | Y | 3 | Y | pilates | 8 | 60 | 2.9 | NA | post | BDI |
| Turkey | Breast Cancer | | NA | 15 | 1.00 | 49.73 (8.71) | Y | 0 | NA | Control | 0 | 0 | 0 | NA | post | BDI |
| (Zhang, et al. 2022) |  | | | | | | | | | | | | | | | |
| China | Breast Cancer | | I-III | 29 | 1.00 | 47.79 (5.14) | Y | 2 | Y | TaiChi/Qigong | 8 | 60 | 2.6 | NA | Post,52weeks | SAS |
| China | Breast Cancer | | I-III | 29 | 1.00 | 47.20 (7.65) | Y | 0 | NA | control | 0 | 0 | 0 | NA | Post,52weeks | SAS |
| (Larkey, et al. 2025) |  | | | | | | | | | | | | | | | |
| United States | Breast Cancer | | 0-III | 57 | 1.00 | 59.6 (7.7) | Y | 1 | Y | TaiChi/Qigong | 8 | 60 | 3.5 | NA | Post,16weeks | POMS-A, POMS-D |
| United States | Breast Cancer | | 0-III | 53 | 1.00 | 59.2 (6.8) | Y | 1 | Y | TaiChi/Qigong | 8 | 60 | 3.5 | NA | Post,16weeks | POMS-A, POMS-D |
| United States | Breast Cancer | | 0-III | 57 | 1.00 | 59.6 (8.4) | Y | 0 | NA | control | 0 | 0 | 0 | NA | Post,16weeks | POMS-A, POMS-D |
| (Knoerl, et al. 2022a) |  | | | | | | | | | | | | | | | |
| United States | Mixed | | I-IV | 23 | 0.96 | 56.8 (10.2) | Y | 2 | Y | Yoga | 8 | 45 | 2.33 | NA | Post | PROMIS-A, PROMIS-D |
| United States | Mixed | | I-IV | 14 | 0.94 | 58 (11) | Y | 0 | NA | control | 0 | 0 | 0 | NA | Post | PROMIS-A, PROMIS-D |
| (Liu, et al. 2022) |  | | | | | | | | | | | | | | | |
| China | Breast Cancer | | I-II | 68 | 1.00 | NA | Y | 1 | Y | yoga | 8 | 90 | 2.5 | 24.71 (3.69) | Post,12weeks | HADS-A,HADS-D |
| China | Breast Cancer | | I-II | 68 | 1.00 | NA | Y | 0 | NA | control | 0 | 0 | 0 | 23.69 (3.16) | Post,12weeks | HADS-A,HADS-D |
| (Kang, et al. 2022) |  | | | | | | | | | | | | | | | |
| Canada | Prostate Cancer | | NA | 25 | 0 | NA | N | 3 | Y | High-intensity interval | 12 | 40 | 4.69 | NA | Post | MAX-PC, STAI, CES-D |
| Canada | Prostate Cancer | | NA | 25 | 0 | NA | N | 0 | NA | control | 0 | 0 | 0 | NA | Post | MAX-PC, STAI, CES-D |
| (Loh, et al. 2019) |  | | | | | | | | | | | | | | | |
| United States | Mixed | | NA | 93 | 0.92 | 68 (5.7) | Y | 7 | NA | Aerobic+Resistance | 6 | 48 | 3 | NA | Post | STAI |
| United States | Mixed | | NA | 96 | 0.91 | 65.5 (4.8) | Y | 0 | NA | control | 0 | 0 | 0 | NA | Post | STAI |
| (Mostafaei, et al. 2021) |  | | | | | | | | | | | | | | | |
| Iran | Breast Cancer | | 0-III | 30 | 1.00 | 48.46 (5.72) | Y | 3 | Y | Mixed | 6 | 30 | 3.58 | NA | Post,4weeks | BDI |
| Iran | Breast Cancer | | 0-III | 30 | 1.00 | 49.6 (7.48) | Y | 0 | NA | control | 0 | 0 | 0 | NA | Post,4weeks | BDI |
| (Cramer, et al. 2016) |  | | | | | | | | | | | | | | | |
| Germany | Colorectal Cancer | | I-III | 27 | 0.37 | 68.70 (9.13) | Y | 1 | Y | yoga | 10 | 90 | 2.4 | NA | Post,12weeks | HADS-A,HADS-D |
| Germany | Colorectal Cancer | | I-III | 27 | 0.41 | 67.81 (10.37) | Y | 0 | NA | control | 0 | 0 | 0 | NA | Post,12weeks | HADS-A,HADS-D |
| (Yang, et al. 2021) |  | | | | | | | | | | | | | | | |
| China | Gastric Cancer | | I-IV | 40 | 0.40 | NA | Y | 5 | Y | TaiChi/Qigong | 4 | 30 | 3.5 | NA | Post | SDS |
| China | Gastric Cancer | | I-IV | 40 | 0.43 | NA | Y | 0 | NA | control | 0 | 0 | 0 | NA | post | SDS |
| (Wen, et al. 2023) |  | | | | | | | | | | | | | | | |
| China | Nasal Cancer | | I-IV | 44 | 0.23 | 45.55 (8.99) | Y | 5 | Y | TaiChi/Qigong | 12 | 40 | 3 | NA | post | GAD-7, PHQ-9 |
| China | Nasal Cancer | | I-IV | 44 | 0.25 | 47.07 (9.43) | Y | 0 | NA | control | 0 | 0 | 0 | NA | post | GAD-7, PHQ-9 |
| (Cheung, et al. 2021) |  | | | | | | | | | | | | | | | |
| China | Lung Cancer | | III-IV | 10 | 0.50 | 61 (12.12) | N | 2 | Y | Aerobic | 12 | 60 | 3.75 | NA | Post,12weeks,39weeks | HADS-A,HADS-D |
| China | Lung Cancer | | III-IV | 9 | 0.33 | 61.11 (7.01) | N | 2 | Y | TaiChi/Qigong | 12 | 60 | 2.88 | NA | Post,12weeks,39weeks | HADS-A,HADS-D |
| China | Lung Cancer | | III-IV | 11 | 0.55 | 58.36 (9.32) | N | 0 | NA | control | 0 | 0 | 0 | NA | Post,12weeks,39weeks | HADS-A,HADS-D |
| (Salchow, et al. 2021) |  | | | | | | | | | | | | | | | |
| Germany | Breast Cancer | | NA | 30 | 1.00 | 54.23 (7.85) | Y | 2 | Y | TaiChi/Qigong | 24 | 90 | 3.57 | NA | Post | HADS-A,HADS-D |
| Germany | Breast Cancer | | NA | 21 | 1.00 | 51.52 (8.41) | Y | 0 | NA | control | 0 | 0 | 0 | NA | Post | HADS-A,HADS-D |
| (Eckert, et al. 2022) |  | | | | | | | | | | | | | | | |
| United States | Blood Cancer | | NA | 30 | 0.64 | NA | Y | 4 | Y | Yoga | 12 | 48 | 2.79 | 29.1 (6.9) | Post,8weeks | PROMIS-A, PROMIS-D |
| United States | Blood Cancer | | NA | 27 | 0.49 | NA | Y | 0 | NA | control | 0 | 0 | 0 | 27.0 (6.7) | Post,8weeks | PROMIS-A, PROMIS-D |
| (Chen, et al. 2015) |  | | | | | | | | | | | | | | | |
| ChinaTaiwan | Lung Cancer | | I-IV | 58 | 0.55 | 64.76 (11.28) | Y | 3 | N | Aerobic | 12 | 40 | 3.8 | NA | Post,12weeks | HADS-A,HADS-D |
| ChinaTaiwan | Lung Cancer | | I-IV | 58 | 0.52 | 63.57 (10.54) | Y | 0 | NA | control | 0 | 0 | 0 | NA | Post,12weeks | HADS-A,HADS-D |
| (Jong, et al. 2018) |  | | | | | | | | | | | | | | | |
| Netherlands | Breast Cancer | | I-III | 45 | 1.00 | 51 (8.0) | Y | 1 | Y | Yoga | 12 | 75 | 2.8 | NA | Post,12weeks | HADS-A,HADS-D |
| Netherlands | Breast Cancer | | I-III | 31 | 1.00 | 51 (7.3) | Y | 0 | NA | control | 0 | 0 | 0 | NA | Post,12weeks | HADS-A,HADS-D |
| (Livingston, et al. 2015) |  | | | | | | | | | | | | | | | |
| Australia | Prostate Cancer | | I-III | 53 | 0 | 66.9 (8.2) | Y | 3 | Y | Aerobic+Resistance | 12 | 50 | 3.75 | 28 (3.5) | Post,12weeks,36weeks | MAX-PC, CES-D |
| Australia | Prostate Cancer | | I-III | 91 | 0 | 64.7 (8.7) | Y | 0 | NA | control | 0 | 0 | 0 | 28 (3.8) | Post,12weeks,36weeks | MAX-PC, CES-D |
| (Ying, et al. 2019) |  | | | | | | | | | | | | | | | |
| China | Breast Cancer | | I-III | 46 | 1.00 | NA | Y | 7 | Y | TaiChi/Qigong | 24 | 60 | 2.84 | NA | Post | GAD-7, PHQ-9 |
| China | Breast Cancer | | I-III | 40 | 1.00 | NA | Y | 0 | NA | control | 0 | 0 | 0 | NA | Post | GAD-7, PHQ-9 |
| (Niels, et al. 2025) |  | | | | | | | | | | | | | | | |
| Germany | Mixed | | II-IV | 11 | 0.64 | 57.2 (12.3) | Y | 2 | Y | Aerobic+Resistance | 24 | 60 | 6.33 | 23.9 (5.0) | Post | HADS-A,HADS-D |
| Germany | Mixed | | II-IV | 7 | 0.43 | 64 (9.3) | Y | 0 | NA | control | 0 | 0 | 0 | 21.7 (2.6) | Post | HADS-A,HADS-D |
| (Wei, et al. 2022) |  | | | | | | | | | | | | | | | |
| China | Breast Cancer | | I-III | 35 | 1.00 | NA | Y | 5 | Y | TaiChi/Qigong | 12 | 30 | 2.77 | 22.86 (2.55) | Post | HADS-A,HADS-D |
| China | Breast Cancer | | I-III | 35 | 1.00 | NA | Y | 0 | NA | control | 0 | 0 | 0 | 23.26 (2.56) | Post | HADS-A,HADS-D |
| (Boing, et al. 2023) |  | | | | | | | | | | | | | | | |
| Brazil | Breast Cancer | | I-III | 25 | 1.00 | 54.3 (10.4) | N | 3 | Y | Pilates | 16 | 60 | 3.5 | NA | Post,24weeks,52weeks | BDI |
| Brazil | Breast Cancer | | I-III | 25 | 1.00 | 55 (9.9) | N | 3 | Y | Aerobic | 16 | 60 | 4.8 | NA | Post,24weeks,52weeks | BDI |
| Brazil | Breast Cancer | | I-III | 24 | 1.00 | 56.8 (11.2) | N | 0 | NA | control | 0 | 0 | 0 | NA | Post,24weeks,52weeks | BDI |
| (Cadmus, et al. 2009) |  | | | | | | | | | | | | | | | |
| United States | Breast Cance | | 0-III | 25 | 1.00 | 50.4 (8.5) | Y | 5 | N | Aerobic | 26 | 30 | 3.5 | 27.9 (5.3) | Post | STAI, CES-D |
| United States | Breast Cance | | 0-III | 25 | 1.00 | 50.5 (11.6) | Y | 0 | NA | control | 0 | 0 | 0 | 28.4 (5.4) | Post | STAI, CES-D |
| United States | Breast Cance | | 0-III | 37 | 1.00 | 59.1 (8.6) | Y | 5 | Y | Aerobic | 26 | 30 | 4 | 30.4 (6) | Post | STAI, CES-D |
| United States | Breast Cance | | 0-III | 37 | 1.00 | 58.6 (8.4) | Y | 0 | NA | control | 0 | 0 | 0 | 30.6 (5.8) | Post | STAI, CES-D |
| (Mutrie, et al. 2007) |  | | | | | | | | | | | | | | | |
| Britain | Breast Cancer | | 0-III | 99 | 1.00 | 51.3 (10.3) | Y | 2 | Y | Aerobic+Resistance | 12 | 45 | 2.97 | 27.3 (5.2) | Post,24weeks | BDI |
| Britain | Breast Cancer | | 0-III | 102 | 1.00 | 51.8 (8.7) | Y | 0 | NA | control | 0 | 0 | 0 | 27.5 (6.0) | Post,24weeks | BDI |
| (Quist, et al. 2020) |  | | | | | | | | | | | | | | | |
| Denmark | Lung Cancer | | III-IV | 110 | 0.50 | 65.2 (8.2) | Y | 2 | Y | Aerobic+Resistance | 12 | 90 | 4.39 | 24.1 (4.4) | post | HADS-A,HADS-D |
| Denmark | Lung Cancer | | III-IV | 108 | 0.52 | 63.5 (8.7) | Y | 0 | NA | control | 0 | 0 | 0 | 24.2 (4.3) | post | HADS-A,HADS-D |
| (Han, et al. 2023) |  | | | | | | | | | | | | | | | |
| SouthKorea | Breast Cancer | | I-III | 23 | 1.00 | 49.91 (7.62) | Y | 2 | Y | Aerobic+Resistance | 12 | 129 | 3.5 | NA | Post,4weeks,24weeks | HADS-A,HADS-D |
| SouthKorea | Breast Cancer | | I-III | 23 | 1.00 | 47.91 (6.24) | Y | 0 | NA | control | 0 | 0 | 0 | NA | Post,4weeks,24weeks | HADS-A,HADS-D |
| (Li, et al. 2024) |  | | | | | | | | | | | | | | | |
| China | Breast Cancer | | I-III | 21 | 1.00 | 51.56 (8.25) | Y | 3 | Y | Aerobic | 12 | 60 | 4.14 | 24.36 (2.98) | Post | SAS |
| China | Breast Cancer | | I-III | 19 | 1.00 | 48.47 (9.28) | Y | 0 | NA | control | 0 | 0 | 0 | 23.48 (3.27) | Post | SAS |
| (Dreyling, et al. 2025) |  | | | | | | | | | | | | | | | |
| Germany | Blood Cancer | | I-III | 16 | 0.31 | 67.3 (7.5) | Y | 2 | Y | Aerobic+Resistance | 12 | 48 | 3.64 | 25.9 (3.8) | Post | HDRS |
| Germany | Blood Cancer | | I-III | 16 | 0.31 | 61.1 (11) | Y | 0 | NA | control | 0 | 0 | 0 | 26.8 (4.3) | Post | HDRS |
| (Tock, et al. 2024) |  | | | | | | | | | | | | | | | |
| Canada | Mixed | | NA | 13 | 0.85 | 30.69 (5.78) | Y | 3 | Y | Aerobic | 12 | 30 | 3.9 | 25.11 (4.56) | Post | PROMIS-A, PROMIS-D |
| Canada | Mixed | | NA | 13 | 0.85 | 34 (5.58) | Y | 0 | NA | control | 0 | 0 | 0 | 24.76 (4.32) | Post | PROMIS-A, PROMIS-D |
| (Munsie, et al. 2022) |  | | | | | | | | | | | | | | | |
| Australia | Mixed | | NA | 20 | 0.32 | 21.9 (3) | Y | 2 | Y | Aerobic+Resistance | 10 | 60 | 3.6 | NA | Post | HADS-A,HADS-D |
| Australia | Mixed | | NA | 18 | 0.43 | 20.3 (2.7) | Y | 0 | NA | Control | 0 | 0 | 0 | NA | Post | HADS-A,HADS-D |
| (Levin, et al. 2018) |  | | | | | | | | | | | | | | | |
| Australia | Mixed | | NA | 9 | 0.67 | 52.7 (10.8) | NA | 4 | Y | Aerobic+Resistance | 12 | 60 | 4.13 | 27.3 (5) | Post | HADS-A,HADS-D |
| Australia | Mixed | | NA | 5 | 0.30 | 63.6 (10.1) | NA | 5 | N | Aerobic | 12 | 48 | 4.3 | 32 (6.5) | Post | HADS-A,HADS-D |
| Australia | Mixed | | NA | 9 | 0.33 | 61.3 (9.6) | NA | 0 | NA | Control | 0 | 0 | 0 | 26.5 (2.9) | Post | HADS-A,HADS-D |
| (da Silveira, et al. 2025) |  | | | | | | | | | | | | | | | |
| Brazil | Breast Cancer | | NA | 11 | 1.00 | 60.9 (8.1) | Y | 2 | Y | Aerobic | 12 | 60 | 3.98 | NA | Post | BAI, BDI |
| Brazil | Breast Cancer | | NA | 11 | 1.00 | 54.5 (11.2) | Y | 0 | NA | Control | 0 | 0 | 0 | NA | Post | BAI, BDI |
| (Cartmel, et al. 2021) |  | | | | | | | | | | | | | | | |
| United States | Ovarian Cancer | | I-IV | 74 | 1.00 | 57.3 (8.8) | Y | 3 | Y | Aerobic | 24 | 48 | 4 | 29 (7.2) | Post | CES-D |
| United States | Ovarian Cancer | | I-IV | 70 | 1.00 | 57.4 (8.5) | Y | 0 | NA | Control | 0 | 0 | 0 | 29.1(6.8) | Post | CES-D |
| (Cavalheri, et al. 2017) |  | | | | | | | | | | | | | | | |
| Australia | Lung Cancer | | I-III | 9 | 0.70 | 66 (10) | Y | 3 | Y | Aerobic+Resistance | 8 | 60 | 3.97 | 25 (5) | Post | HADS-A,HADS-D |
| Australia | Lung Cancer | | I-III | 8 | 0.75 | 68 (9) | Y | 0 | NA | Control | 0 | 0 | 0 | 27 (6) | Post | HADS-A,HADS-D |
| (de Souza, et al. 2025) |  | | | | | | | | | | | | | | | |
| Brazil | Breast Cancer | | I-III | 11 | 1.00 | NA | N | 2 | Y | Aerobic | 12 | 60 | 3.98 | NA | Post | BAI, BDI |
| Brazil | Breast Cancer | | I-III | 10 | 1.00 | NA | N | 2 | Y | Aerobic | 12 | 60 | 3.98 | NA | Post | BAI, BDI |
| Brazil | Breast Cancer | | I-III | 11 | 1.00 | NA | N | 0 | NA | Control | 0 | 0 | 0 | NA | Post | BAI, BDI |
| (Chen, et al. 2013) |  | | | | | | | | | | | | | | | |
| China | Breast Cancer | | 0-III | 49 | 1.00 | 45.3 (6.3) | Y | 5 | Y | TaiChi/Qigong | 6 | 40 | 2.39 | NA | Post,4weeks,12weeks | CES-D |
| China | Breast Cancer | | 0-III | 47 | 1.00 | 44.7 (9.7) | Y | 0 | NA | Control | 0 | 0 | 0 | NA | Post,4weeks,12weeks | CES-D |
| (Ho, et al. 2016) |  | | | | | | | | | | | | | | | |
| China | Breast Cancer | | 0-III | 69 | 1.00 | 48.6 (7.7) | Y | 2 | Y | Aerobic | 3 | 90 | 3.1 | NA | Post | HADS-A,HADS-D |
| China | Breast Cancer | | 0-III | 70 | 1.00 | 49.1 (8.7) | Y | 0 | NA | Control | 0 | 0 | 0 | NA | Post | HADS-A,HADS-D |
| (Özkan, et al. 2022) |  | | | | | | | | | | | | | | | |
| Turkey | Breast Cancer | | I-III | 10 | 1.00 | 42.1 (9.06) | Y | 3 | Y | Aerobic+Resistance | 6 | 40 | 4.03 | 27.83 (2.45) | 1weeks | BAI, BDI |
| Turkey | Breast Cancer | | I-III | 10 | 1.00 | 48.9 (8.29) | Y | 0 | NA | Control | 0 | 0 | 0 | 30.55 (5.22) | 1weeks | BAI, BDI |
| (Schmidt, et al. 2015) |  | | | | | | | | | | | | | | | |
| Germany | Breast Cancer | | I-IV | 49 | 1.00 | 52.2 (9.9) | Y | 2 | Y | Resistance | 12 | 60 | 6 | 25.7 (4.6) | Post | CES-D |
| Germany | Breast Cancer | | I-IV | 44 | 1.00 | 53.3 (10.2) | Y | 0 | NA | Control | 0 | 0 | 0 | 26.3 (4.9) | Post | CES-D |
| (Ergun, et al. 2013) |  | | | | | | | | | | | | | | | |
| Turkey | Breast Cancer | | NA | 20 | 1.00 | 49.65 (8.25) | Y | 6 | Y | Aerobic+Resistance | 12 | 40 | 4.03 | 26.55 (4.40) | Post | BDI |
| Turkey | Breast Cancer | | NA | 20 | 1.00 | 55.05 (6.85) | Y | 3 | N | Resistance | 12 | 30 | 4.8 | 28.64 (5.15) | Post | BDI |
| Turkey | Breast Cancer | | NA | 20 | 1.00 | 50.3 (10.37) | Y | 0 | NA | Control | 0 | 0 | 0 | 28.60 (5.10) | Post | BDI |
| (Gokal, et al. 2016) |  | | | | | | | | | | | | | | | |
| Britain | Breast Cancer | | I-III | 25 | 1.00 | 52.08 (11.7) | Y | 5 | N | Aerobic | 12 | 30 | 4.3 | 27.2 (4.82) | Post | HADS-A,HADS-D |
| Britain | Breast Cancer | | I-III | 25 | 1.00 | 52.36 (8.9) | Y | 0 | NA | Control | 0 | 0 | 0 | 28.25 (5.83) | Post | HADS-A,HADS-D |
| (Steindorf, et al. 2014) |  | | | | | | | | | | | | | | | |
| Germany | Breast Cancer | | 0-III | 75 | 1.00 | 55.2 (11.7) | Y | 2 | Y | Resistance | 12 | 60 | 6 | 27.3 (5.7) | Post | CES-D |
| Germany | Breast Cancer | | 0-III | 76 | 1.00 | 56.4 (10.3) | Y | 0 | NA | Control | 0 | 0 | 0 | 27.6 (5.7) | Post | CES-D |
| (Li, et al. 2023) |  | | | | | | | | | | | | | | | |
| China | Mixed | | I-III | 47 | 0.60 | 28.4 (6.28) | Y | 4 | Y | Aerobic | 8 | 35 | 4.3 | NA | 1weeks,12weeks | HADS-A,HADS-D |
| China | Mixed | | I-III | 48 | 0.63 | 31.21 (5.54) | Y | 0 | NA | Control | 0 | 0 | 0 | NA | 1weeks,12weeks | HADS-A,HADS-D |
| (Charati, et al. 2022) |  | | | | | | | | | | | | | | | |
| Iran | Breast Cancer | | NA | 35 | 1.00 | 38.14 (10.70) | Y | 5 | Y | Aerobic | 5 | 30 | 2.67 | NA | Post | HADS-A,HADS-D |
| Iran | Breast Cancer | | NA | 35 | 1.00 | 42.63 (8.11) | Y | 0 | NA | Control | 0 | 0 | 0 | NA | Post | HADS-A,HADS-D |
| (Yao, et al. 2022) |  | | | | | | | | | | | | | | | |
| China | Breast Cancer | | I-III | 36 | 1.00 | 45.3 (8.5) | Y | 2 | Y | TaiChi/Qigong | 8 | 60 | 2.61 | NA | Post,4weeks | HADS-D |
| China | Breast Cancer | | I-III | 36 | 1.00 | 48.6 (7.8) | Y | 0 | NA | Control | 0 | 0 | 0 | NA | Post,4weeks | HADS-D |

**Supplementary 3: Arm-based forest plot** (**Depression)**

**
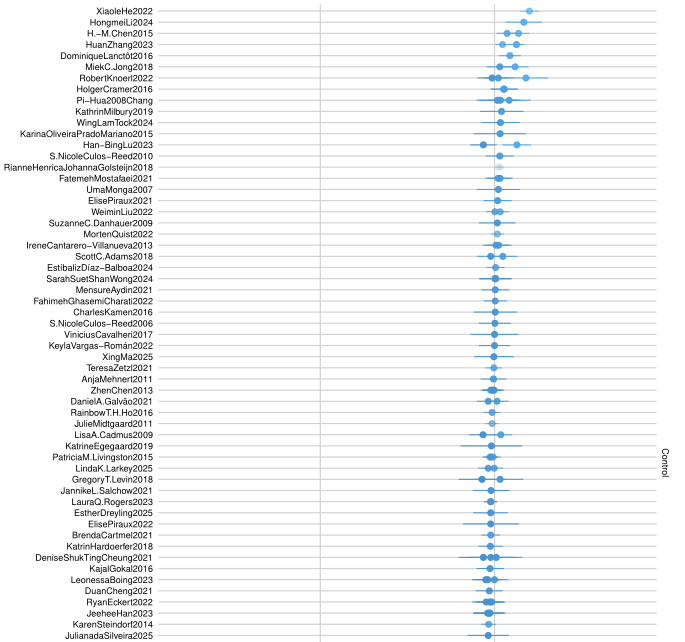

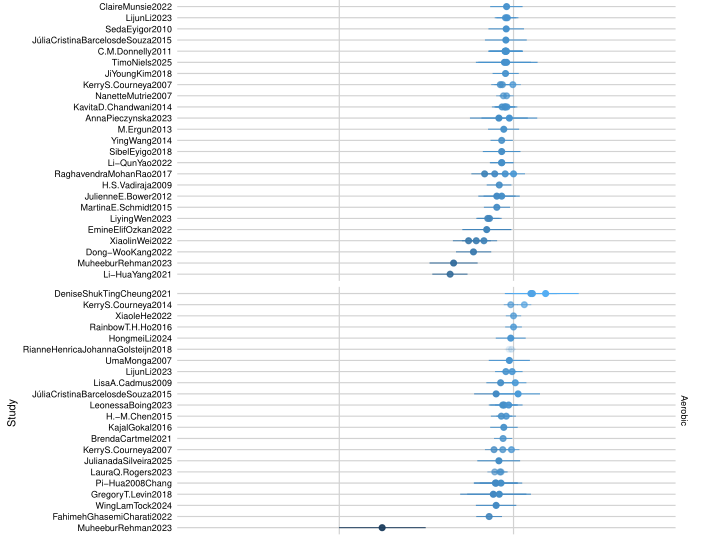
**

**
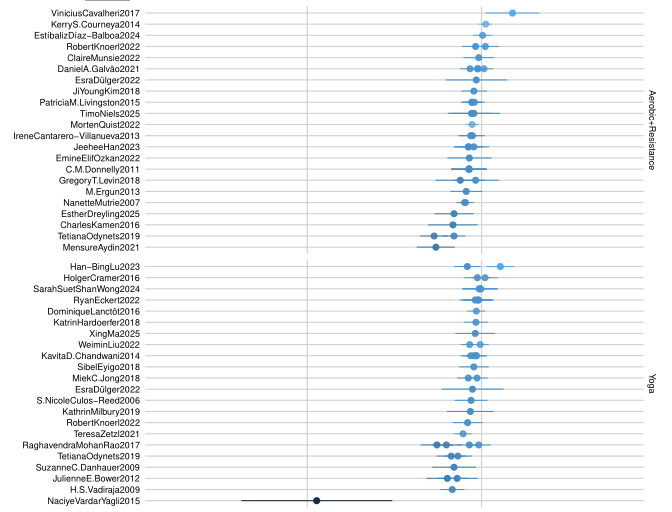

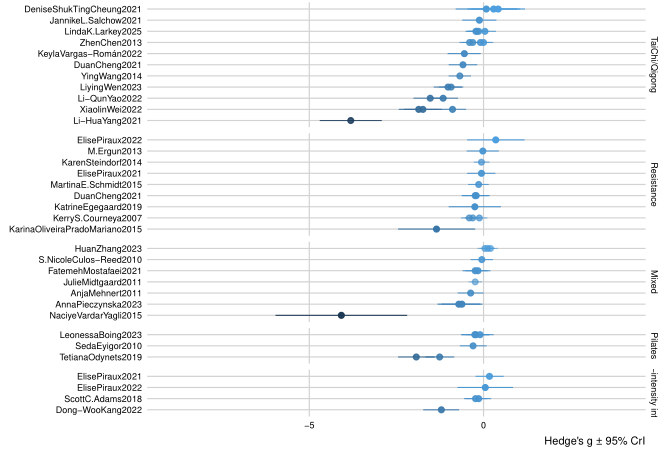
**

**Supplementary 4: Arm-based forest plot** (**Anxiety)**

**
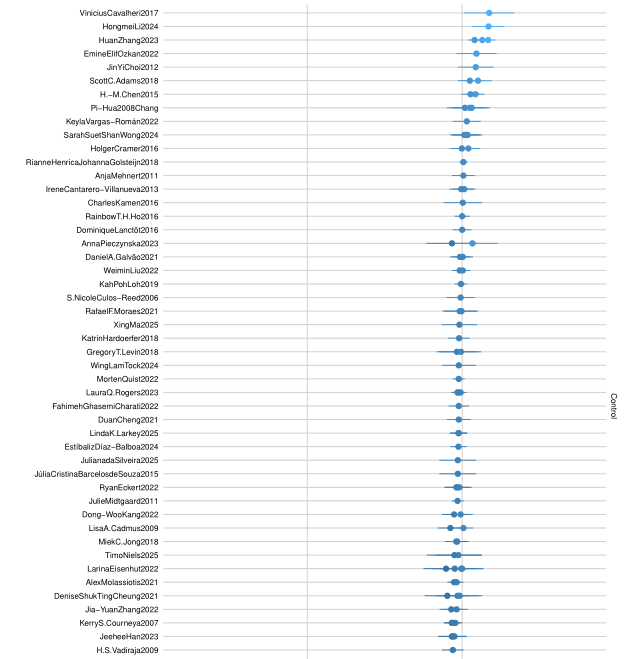

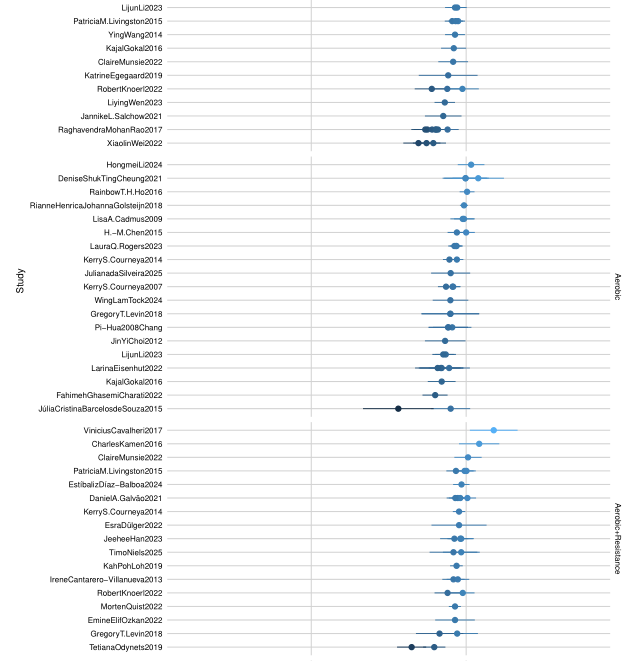
**

**
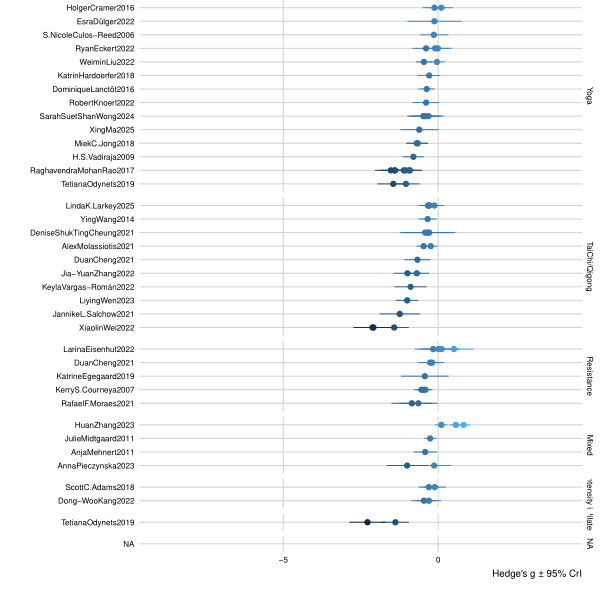
**

**Supplementary 5: Risk of Bias in Individual Studies**

**Risk of Bias for Depressive Outcomes**

| **Author, Year** | **Randomization process** | **Deviation from intervention** | **Missing outcome data** | **Outcome measurement** | **Reporting selection** | **Overall bias** |
| --- | --- | --- | --- | --- | --- | --- |
| EstíbalizDíaz-Balboa2024 | Some concerns | Some concerns | Low risk | High risk | Low risk | High risk |
| Han-BingLu2023 | Low risk | Some concerns | Some concerns | Some concerns | Low risk | Some concerns |
| UmaMonga2007 | Some concerns | Some concerns | Low risk | High risk | Low risk | High risk |
| KarinaOliveiraPradoMariano2015 | Some concerns | Some concerns | High risk | High risk | Some concerns | High risk |
| KatrineEgegaard2019 | Low risk | Some concerns | Some concerns | High risk | Low risk | High risk |
| AnnaPieczyńska2023 | Some concerns | Some concerns | Some concerns | Low risk | Some concerns | Some concerns |
| TetianaOdynets2019 | Some concerns | Some concerns | Some concerns | High risk | Some concerns | High risk |
| C.M.Donnelly2011 | Some concerns | Some concerns | Some concerns | High risk | Some concerns | Some concerns |
| KathrinMilbury2019 | Some concerns | Some concerns | Some concerns | High risk | Some concerns | High risk |
| JulienneE.Bower2012 | Some concerns | Some concerns | Some concerns | High risk | Some concerns | Some concerns |
| Pi-Hua2008Chang | Some concerns | Some concerns | Some concerns | High risk | Some concerns | Some concerns |
| JulieMidtgaard2011 | Some concerns | Some concerns | Some concerns | High risk | Some concerns | High risk |
| CharlesKamen2016 | Some concerns | Some concerns | Some concerns | High risk | Some concerns | High risk |
| IreneCantarero-Villanueva2013 | Low risk | Some concerns | Some concerns | High risk | Some concerns | High risk |
| SarahSuetShanWong2024 | Some concerns | Some concerns | Some concerns | High risk | Some concerns | High risk |
| C.M.Donnelly2011 | Low risk | Some concerns | Low risk | High risk | Low risk | High risk |
| KerryS.Courneya2014 | Low risk | Some concerns | Low risk | High risk | Some concerns | High risk |
| RianneHenricaJohannaGolsteijn2018 | Low risk | Some concerns | Some concerns | High risk | Low risk | High risk |
| S.NicoleCulos-Reed2006 | Some concerns | Some concerns | Some concerns | High risk | Some concerns | High risk |
| ElisePiraux2022 | Some concerns | Low risk | Some concerns | High risk | High risk | High risk |
| S.NicoleCulos-Reed2010 | Some concerns | Some concerns | High risk | High risk | Some concerns | High risk |
| LauraQ.Rogers2023 | Low risk | Low risk | Low risk | High risk | Some concerns | High risk |
| DominiqueLanctôt2016 | High risk | High risk | Some concerns | High risk | Some concerns | High risk |
| KerryS.Courneya2007 | Low risk | Low risk | Low risk | High risk | Some concerns | High risk |
| SibelEyigo2018 | Some concerns | Some concerns | High risk | High risk | Some concerns | High risk |
| DuanCheng2021 | Some concerns | Some concerns | Some concerns | High risk | Some concerns | High risk |
| XingMa2025 | Low risk | Some concerns | Low risk | High risk | Some concerns | High risk |
| NaciyeVardarYagli2015 | High risk | Some concerns | Low risk | High risk | Some concerns | High risk |
| DanielA.Galvão2021 | Some concerns | Low risk | Some concerns | High risk | Some concerns | High risk |
| ElisePiraux2021 | Some concerns | Low risk | Some concerns | High risk | Some concerns | High risk |
| EsraDülger2022 | Some concerns | Some concerns | Low risk | High risk | Some concerns | High risk |
| JiYoungKim2018 | Some concerns | Low risk | Some concerns | High risk | Some concerns | High risk |
| RobertKnoerl2022 | Some concerns | Some concerns | Some concerns | High risk | Some concerns | High risk |
| ScottC.Adams2018 | Some concerns | Low risk | Low risk | High risk | Some concerns | High risk |
| KavitaD.Chandwani2014 | Some concerns | Some concerns | Some concerns | High risk | Some concerns | High risk |
| KatrinHardoerfer2018 | Some concerns | Some concerns | Some concerns | High risk | Some concerns | High risk |
| MensureAydin2021 | Some concerns | Some concerns | Low risk | High risk | Some concerns | High risk |
| XiaoleHe2022 | Low risk | Low risk | Low risk | High risk | Some concerns | High risk |
| RaghavendraMohanRao2017 | Low risk | Some concerns | Some concerns | High risk | Some concerns | High risk |
| SuzanneC.Danhauer2009 | Some concerns | Some concerns | High risk | High risk | Some concerns | High risk |
| MuheeburRehman2023 | Some concerns | Some concerns | Low risk | High risk | Some concerns | High risk |
| HuanZhang2023 | Some concerns | Some concerns | Low risk | High risk | Some concerns | High risk |
| AnjaMehnert2011 | Some concerns | Some concerns | Some concerns | High risk | Some concerns | High risk |
| KeylaVargas-Román2022 | Low risk | Some concerns | Some concerns | High risk | Some concerns | High risk |
| TeresaZetzl2021 | Some concerns | Some concerns | Some concerns | High risk | Some concerns | High risk |
| H.S.Vadiraja2009 | Low risk | Some concerns | High risk | High risk | Some concerns | High risk |
| SedaEyigor2010 | Some concerns | High risk | High risk | High risk | Some concerns | High risk |
| LindaK.Larkey2025 | Low risk | Some concerns | Some concerns | Some concerns | Low risk | Some concerns |
| RobertKnoerl2022 | Some concerns | Some concerns | High risk | High risk | Some concerns | High risk |
| WeiminLiu2022 | Low risk | Some concerns | Low risk | High risk | Some concerns | High risk |
| Dong-WooKang2022 | Low risk | Some concerns | Low risk | High risk | Some concerns | High risk |
| FatemehMostafaei2021 | Some concerns | Low risk | Low risk | Some concerns | Some concerns | Some concerns |
| HolgerCramer2016 | Low risk | Low risk | Some concerns | High risk | Low risk | High risk |
| Li-HuaYang2021 | Low risk | Some concerns | Low risk | High risk | Some concerns | High risk |
| LiyingWen2023 | Low risk | Some concerns | Some concerns | Some concerns | Some concerns | Some concerns |
| DeniseShukTingCheung2021 | Low risk | Some concerns | High risk | High risk | Some concerns | High risk |
| JannikeL.Salchow2021 | Some concerns | Some concerns | High risk | High risk | Some concerns | High risk |
| RyanEckert2022 | Some concerns | Low risk | Some concerns | Some concerns | Some concerns | Some concerns |
| H.-M.Chen2015 | Low risk | Low risk | High risk | High risk | Some concerns | High risk |
| MiekC.Jong2018 | Some concerns | High risk | Some concerns | Some concerns | Some concerns | High risk |
| PatriciaM.Livingston2015 | Some concerns | Low risk | Low risk | Some concerns | Low risk | Some concerns |
| YingWang2014 | Low risk | Some concerns | Some concerns | Some concerns | Some concerns | Some concerns |
| TimoNiels2025 | Some concerns | Low risk | Some concerns | High risk | Some concerns | High risk |
| XiaolinWei2022 | Some concerns | Low risk | Some concerns | High risk | Low risk | High risk |
| LeonessaBoing2023 | Some concerns | Some concerns | Some concerns | Some concerns | Low risk | Some concerns |
| LisaA.Cadmus2009 | Low risk | Low risk | Low risk | High risk | Some concerns | High risk |
| NanetteMutrie2007 | Low risk | Low risk | Some concerns | Some concerns | Low risk | Some concerns |
| MortenQuist2022 | Low risk | Some concerns | Some concerns | Some concerns | Low risk | Some concerns |
| JeeheeHan2023 | Low risk | Some concerns | Some concerns | High risk | Some concerns | High risk |
| HongmeiLi2024 | Some concerns | Some concerns | Low risk | High risk | Low risk | High risk |
| EstherDreyling2025 | Low risk | High risk | Low risk | High risk | Some concerns | High risk |
| WingLamTock2024 | Low risk | Low risk | Low risk | Some concerns | Some concerns | Some concerns |
| ClaireMunsie2022 | Low risk | Some concerns | Some concerns | High risk | Some concerns | High risk |
| GregoryT.Levin2018 | Some concerns | High risk | High risk | Some concerns | Some concerns | High risk |
| JulianadaSilveira2025 | Some concerns | Some concerns | Low risk | High risk | Low risk | High risk |
| BrendaCartmel2021 | Some concerns | Low risk | Some concerns | High risk | Some concerns | High risk |
| ViniciusCavalheri2017 | Low risk | Some concerns | Low risk | Some concerns | Some concerns | Some concerns |
| JúliaCristinaBarcelosdeSouza2015 | Some concerns | High risk | Some concerns | Some concerns | Low risk | High risk |
| ZhenChen2013 | Some concerns | Low risk | Low risk | High risk | Some concerns | High risk |
| RainbowT.H.Ho2016 | Low risk | Low risk | Low risk | Some concerns | Some concerns | Some concerns |
| EmineElifOzkan2022 | Some concerns | Some concerns | Low risk | High risk | Some concerns | High risk |
| MartinaE.Schmidt2015 | Low risk | Low risk | Low risk | Some concerns | Some concerns | Some concerns |
| M.Ergun2013 | Some concerns | High risk | Low risk | High risk | Some concerns | High risk |
| KajalGokal2016 | Some concerns | Low risk | Some concerns | High risk | Some concerns | High risk |
| KarenSteindorf2014 | Low risk | Low risk | Low risk | Some concerns | Some concerns | Some concerns |
| LijunLi2023 | Low risk | Some concerns | Some concerns | High risk | Some concerns | High risk |
| FahimehGhasemiCharati2022 | Some concerns | High risk | High risk | Some concerns | Some concerns | High risk |
| Li-QunYao2022 | Low risk | Some concerns | Low risk | High risk | Low risk | High risk |

**Risk of Bias in Anxiety Outcomes**

| **Author, Year** | **Randomization process** | **Deviation from intervention** | **Missing outcome data** | **Outcome measurement** | **Reporting selection** | **Overall bias** |
| --- | --- | --- | --- | --- | --- | --- |
| EstíbalizDíaz-Balboa2024 | Some concerns | Some concerns | Low risk | High risk | Low risk | High risk |
| AlexMolassiotis2021 | Low risk | Some concerns | Some concerns | High risk | Low risk | High risk |
| KatrineEgegaard2019 | Low risk | Some concerns | Some concerns | High risk | Low risk | High risk |
| AnnaPieczyńska2023 | Some concerns | Some concerns | Some concerns | Low risk | Some concerns | Some concerns |
| TetianaOdynets2019 | Some concerns | Some concerns | Some concerns | High risk | Some concerns | High risk |
| Pi-Hua2008Chang | Some concerns | Some concerns | Some concerns | High risk | Some concerns | Some concerns |
| JulieMidtgaard2011 | Some concerns | Some concerns | Some concerns | High risk | Some concerns | High risk |
| CharlesKamen2016 | Some concerns | Some concerns | Some concerns | High risk | Some concerns | High risk |
| IreneCantarero-Villanueva2013 | Low risk | Some concerns | Some concerns | High risk | Some concerns | High risk |
| SarahSuetShanWong2024 | Some concerns | Some concerns | Some concerns | High risk | Some concerns | High risk |
| KerryS.Courneya2014 | Low risk | Some concerns | Low risk | High risk | Some concerns | High risk |
| RianneHenricaJohannaGolsteijn2018 | Low risk | Some concerns | Some concerns | High risk | Low risk | High risk |
| S.NicoleCulos-Reed2006 | Some concerns | Some concerns | Some concerns | High risk | Some concerns | High risk |
| LauraQ.Rogers2023 | Low risk | Low risk | Low risk | High risk | Some concerns | High risk |
| DominiqueLanctôt2016 | High risk | Some concerns | Some concerns | High risk | Some concerns | High risk |
| KerryS.Courneya2007 | Low risk | Low risk | Low risk | High risk | Some concerns | High risk |
| JinYiChoi2012 | Some concerns | Some concerns | Some concerns | High risk | Some concerns | High risk |
| DuanCheng2021 | Some concerns | Some concerns | Some concerns | High risk | Some concerns | High risk |
| XingMa2025 | Low risk | Some concerns | Low risk | High risk | Some concerns | High risk |
| DanielA.Galvão2021 | Some concerns | Low risk | Some concerns | High risk | Some concerns | High risk |
| LarinaEisenhut2022 | Low risk | Low risk | Low risk | High risk | Some concerns | High risk |
| EsraDülger2022 | Some concerns | Some concerns | Low risk | High risk | Some concerns | High risk |
| RobertKnoerl2022 | Some concerns | Some concerns | Some concerns | High risk | Some concerns | High risk |
| ScottC.Adams2018 | Some concerns | Low risk | Low risk | High risk | Some concerns | High risk |
| KatrinHardoerfer2018 | Some concerns | Some concerns | Some concerns | High risk | Some concerns | High risk |
| RafaelF.Moraes2021 | Some concerns | Some concerns | Low risk | High risk | Some concerns | High risk |
| RaghavendraMohanRao2017 | Low risk | Some concerns | Some concerns | High risk | Some concerns | High risk |
| HuanZhang2023 | Some concerns | Some concerns | Low risk | High risk | Some concerns | High risk |
| AnjaMehnert2011 | Some concerns | Some concerns | Some concerns | High risk | Some concerns | High risk |
| KeylaVargas-Román2022 | Low risk | Some concerns | Some concerns | High risk | Some concerns | High risk |
| H.S.Vadiraja2009 | Low risk | Some concerns | High risk | High risk | Some concerns | High risk |
| Jia-YuanZhang2022 | Low risk | Low risk | Some concerns | Some concerns | Low risk | Some concerns |
| LindaK.Larkey2025 | Low risk | Some concerns | Some concerns | Some concerns | Low risk | Some concerns |
| RobertKnoerl2022 | Some concerns | Some concerns | High risk | High risk | Some concerns | High risk |
| WeiminLiu2022 | Low risk | Some concerns | Low risk | High risk | Some concerns | High risk |
| Dong-WooKang2022 | Low risk | Some concerns | Low risk | High risk | Some concerns | High risk |
| KahPohLoh2019 | Some concerns | Some concerns | Some concerns | High risk | High risk | High risk |
| HolgerCramer2016 | Low risk | Low risk | Some concerns | High risk | Low risk | High risk |
| LiyingWen2023 | Low risk | Some concerns | Some concerns | Some concerns | Some concerns | Some concerns |
| DeniseShukTingCheung2021 | Low risk | Some concerns | High risk | High risk | Some concerns | High risk |
| JannikeL.Salchow2021 | Some concerns | Some concerns | High risk | High risk | Some concerns | High risk |
| RyanEckert2022 | Some concerns | Some concerns | High risk | Some concerns | Some concerns | High risk |
| H.-M.Chen2015 | Low risk | Low risk | Some concerns | High risk | Some concerns | High risk |
| MiekC.Jong2018 | Some concerns | High risk | Some concerns | Some concerns | Some concerns | High risk |
| PatriciaM.Livingston2015 | Some concerns | Low risk | Low risk | Some concerns | Low risk | Some concerns |
| YingWang2014 | Low risk | Some concerns | Some concerns | Some concerns | Some concerns | Some concerns |
| TimoNiels2025 | Some concerns | Low risk | High risk | High risk | Some concerns | High risk |
| XiaolinWei2022 | Some concerns | Low risk | Some concerns | High risk | Low risk | High risk |
| LisaA.Cadmus2009 | Low risk | Low risk | Low risk | High risk | Some concerns | High risk |
| MortenQuist2022 | Low risk | Some concerns | Some concerns | Some concerns | Low risk | Some concerns |
| JeeheeHan2023 | Low risk | Some concerns | Some concerns | High risk | Some concerns | High risk |
| HongmeiLi2024 | Some concerns | Some concerns | Low risk | High risk | Low risk | High risk |
| WingLamTock2024 | Low risk | Low risk | Low risk | Some concerns | Some concerns | Some concerns |
| ClaireMunsie2022 | Low risk | Some concerns | Some concerns | High risk | Some concerns | High risk |
| GregoryT.Levin2018 | Some concerns | High risk | High risk | Some concerns | Some concerns | High risk |
| JulianadaSilveira2025 | Some concerns | Some concerns | Low risk | High risk | Low risk | High risk |
| ViniciusCavalheri2017 | Low risk | Some concerns | Low risk | Some concerns | Some concerns | Some concerns |
| JúliaCristinaBarcelosdeSouza2015 | Some concerns | High risk | Some concerns | Some concerns | Low risk | High risk |
| RainbowT.H.Ho2016 | Low risk | Low risk | Low risk | Some concerns | Some concerns | Some concerns |
| EmineElifOzkan2022 | Some concerns | Some concerns | Low risk | High risk | Some concerns | High risk |
| KajalGokal2016 | Some concerns | Low risk | Some concerns | High risk | Some concerns | High risk |
| LijunLi2023 | Low risk | Some concerns | Some concerns | High risk | Some concerns | High risk |
| FahimehGhasemiCharati2022 | Some concerns | High risk | High risk | Some concerns | Some concerns | High risk |

**Supplementary 6: Global and Local Inconsistency Evaluated (Depression)**

Design-by-treatment interaction model

| Model/Test | Q | df | p-value |
| --- | --- | --- | --- |
| Between-designs Q (graph-theoretical NMA) | 20.87 | 21 | 0.4669 |
| Full design-by-treatment (random-effects) | 13.13 | 21 | 0.9041 |

Node-splitting results

| **Comparison** | **k** | **prop** | **NMA (smd)** | **Direct (smd)** | **Indirect (smd)** | **Diff** | **z** | **p-value** |
| --- | --- | --- | --- | --- | --- | --- | --- | --- |
| Aerobic: Aerobic + Resistance | 2 | 0.18 | -0.1121 | -0.0427 | -0.1271 | 0.0844 | 0.3600 | 0.7183 |
| Aerobic: Pilates | 1 | 0.35 | 0.0527 | -0.0469 | 0.1059 | -0.1528 | -0.4000 | 0.6883 |
| Aerobic: Resistance | 1 | 0.25 | -0.2872 | 0.0095 | -0.3887 | 0.3982 | 1.4600 | 0.1441 |
| Aerobic: Tai Chi and Qigong | 1 | 0.05 | 0.0184 | 0.2281 | 0.0076 | 0.2205 | 0.4100 | 0.6810 |
| Aerobic+ Resistance: Pilates | 1 | 0.32 | 0.1648 | 0.4605 | 0.0251 | 0.4355 | 1.1100 | 0.2652 |
| Aerobic+ Resistance: Resistance | 1 | 0.11 | -0.1751 | -0.4168 | -0.1467 | -0.2702 | -0.6700 | 0.5017 |
| Aerobic+ Resistance: Yoga | 3 | 0.19 | 0.0515 | 0.0744 | 0.0460 | 0.0285 | 0.1200 | 0.9055 |
| High-intensity interval: Control | 4 | 0.90 | -0.0433 | -0.0587 | 0.0963 | -0.1550 | -0.2600 | 0.7962 |
| Mixed: Control | 6 | 0.99 | -0.2712 | -0.2754 | 0.3975 | -0.6729 | -0.4500 | 0.6502 |
| Pilates: Control | 2 | 0.54 | -0.3619 | -0.0626 | -0.7133 | 0.6507 | 1.8500 | 0.0643 |
| Resistance: Control | 9 | 0.86 | -0.0220 | 0.0216 | -0.2940 | 0.3156 | 1.0300 | 0.3015 |
| High-intensity interval: Resistance | 2 | 0.42 | -0.0213 | 0.1100 | -0.1178 | 0.2278 | 0.5800 | 0.5647 |
| Mixed: Yoga | 1 | < 0.01 | -0.0226 | 0.6445 | -0.0284 | 0.6729 | 0.4500 | 0.6502 |
| Pilates: Yoga | 1 | 0.30 | -0.1133 | -0.6473 | 0.1205 | -0.7678 | -1.9200 | 0.0549 |
| Resistance: Tai Chi and Qigong | 1 | 0.18 | 0.3057 | 0.3573 | 0.2946 | 0.0627 | 0.1700 | 0.8635 |

**Supplementary 7: Funnel plot (Depression)**

**
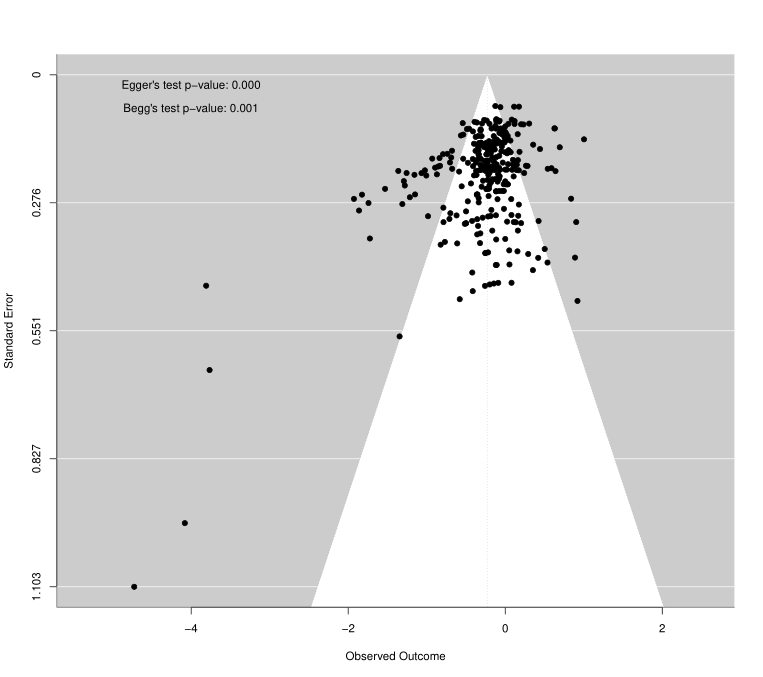
**

**Supplementary 8: Global and Local Inconsistency Evaluated (Anxiety)**

Design-by-treatment interaction model

| Model/Test | Q | df | p-value |
| --- | --- | --- | --- |
| Between-designs Q (graph-theoretical NMA) | 7.18 | 13 | 0.8926 |
| Full design-by-treatment random-effects | 6.56 | 13 | 0.9236 |

Node-splitting results

| **Comparison** | **k** | **prop** | **NMA (smd)** | **Direct (smd)** | **Indirect (smd)** | **Diff** | **z** | **p-value** |
| --- | --- | --- | --- | --- | --- | --- | --- | --- |
| Aerobic: Aerobic + Resistance | 2 | 0.25 | -0.1653 | -0.1701 | -0.1638 | -0.0064 | -0.0400 | 0.9696 |
| Aerobic: Resistance | 2 | 0.56 | -0.1272 | -0.1200 | -0.1365 | 0.0166 | 0.0700 | 0.9414 |
| Aerobic: Tai Chi and Qigong | 1 | 0.04 | 0.0318 | 0.3865 | 0.0178 | 0.3688 | 0.7600 | 0.4487 |
| Aerobic+ Resistance: Pilates | 1 | 0.85 | 0.4050 | 0.3447 | 0.7350 | -0.3903 | -0.5300 | 0.5951 |
| Aerobic+ Resistance: Yoga | 3 | 0.20 | 0.1445 | 0.0264 | 0.1743 | -0.1480 | -0.6800 | 0.4956 |
| Resistance: Control | 5 | 0.80 | -0.1367 | -0.1684 | -0.0107 | -0.1578 | -0.5900 | 0.5585 |
| Pilates: Yoga | 1 | 0.74 | -0.2606 | -0.3453 | -0.0213 | -0.3240 | -0.5300 | 0.5951 |
| Resistance: Tai Chi and Qigong | 1 | 0.21 | 0.1590 | 0.4404 | 0.0855 | 0.3550 | 1.1100 | 0.2652 |

**Supplementary 9: Funnel plot (****Anxiety)**


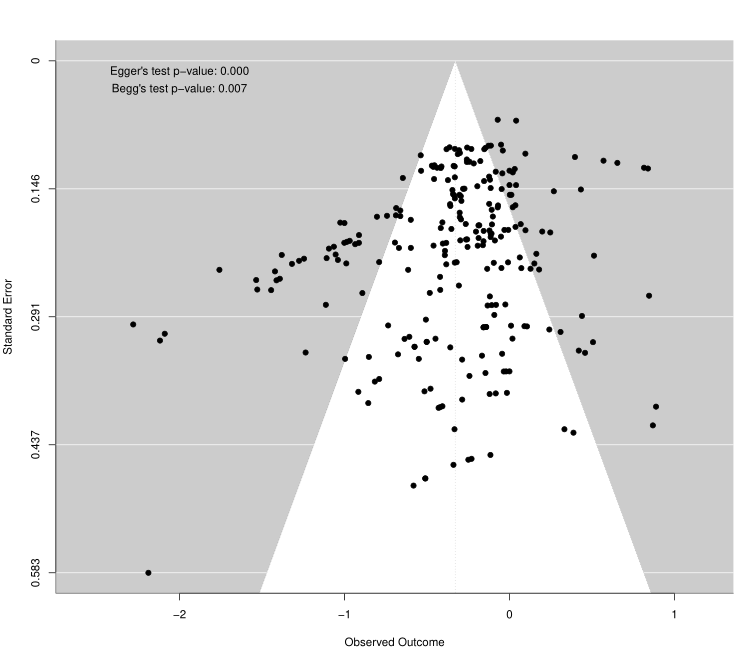


**Supplementary 10:Subgroup analysis**

**Depressive Model Coefficients (SMD)**

| Term | Estimate | SE | 95% CrI |
| --- | --- | --- | --- |
| Intercept | -0.128 | 0.128 | [-0.379, 0.128] |
| radio_chemoY | -0.270 | 0.134 | [-0.538, -0.010] |

## **Depressive** **Subgroup Net Effects (SMD)**

| Subgroup | Raw SMD | SMD vs Control | 95% CrI Lower | 95% CrI Upper |
| --- | --- | --- | --- | --- |
| No Radio/Chemo (N) | -0.128 | -0.032 | -0.283 | 0.224 |
| Radio/Chemo (Y) | -0.398 | -0.302 | -0.413 | -0.196 |

**
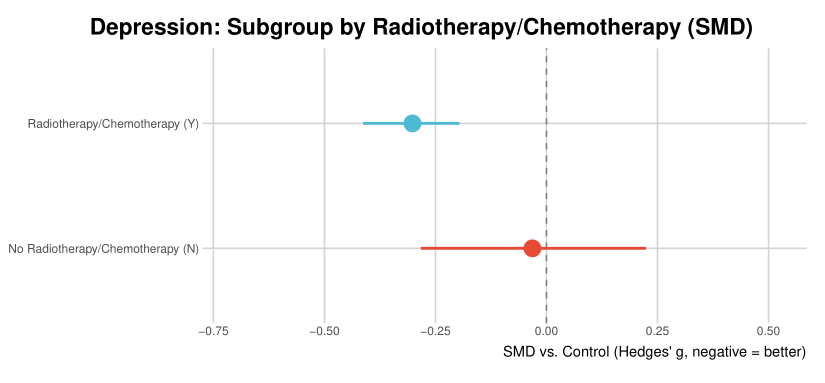
**

## **Anxiety Model Coefficients (SMD)**

| Term | Estimate | SE | 95% CrI |
| --- | --- | --- | --- |
| Intercept | -0.340 | 0.145 | [-0.627, -0.058] |
| radio_chemoY | -0.069 | 0.149 | [-0.359, 0.226] |

## **Anxiety Subgroup Net Effects (SMD)**

| Subgroup | Raw SMD | SMD vs Control | 95% CrI Lower | 95% CrI Upper |
| --- | --- | --- | --- | --- |
| No Radio/Chemo (N) | -0.339 | -0.219 | -0.507 | 0.062 |
| Radio/Chemo (Y) | -0.409 | -0.289 | -0.399 | -0.177 |

**
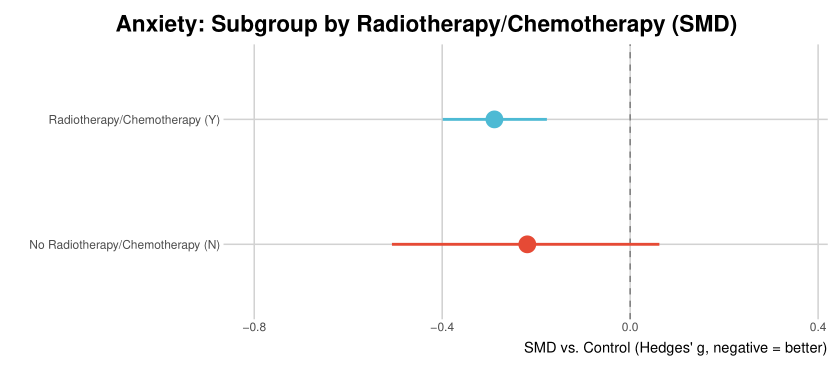
**

**Supplementary 11:Sensitivity analyses**

**
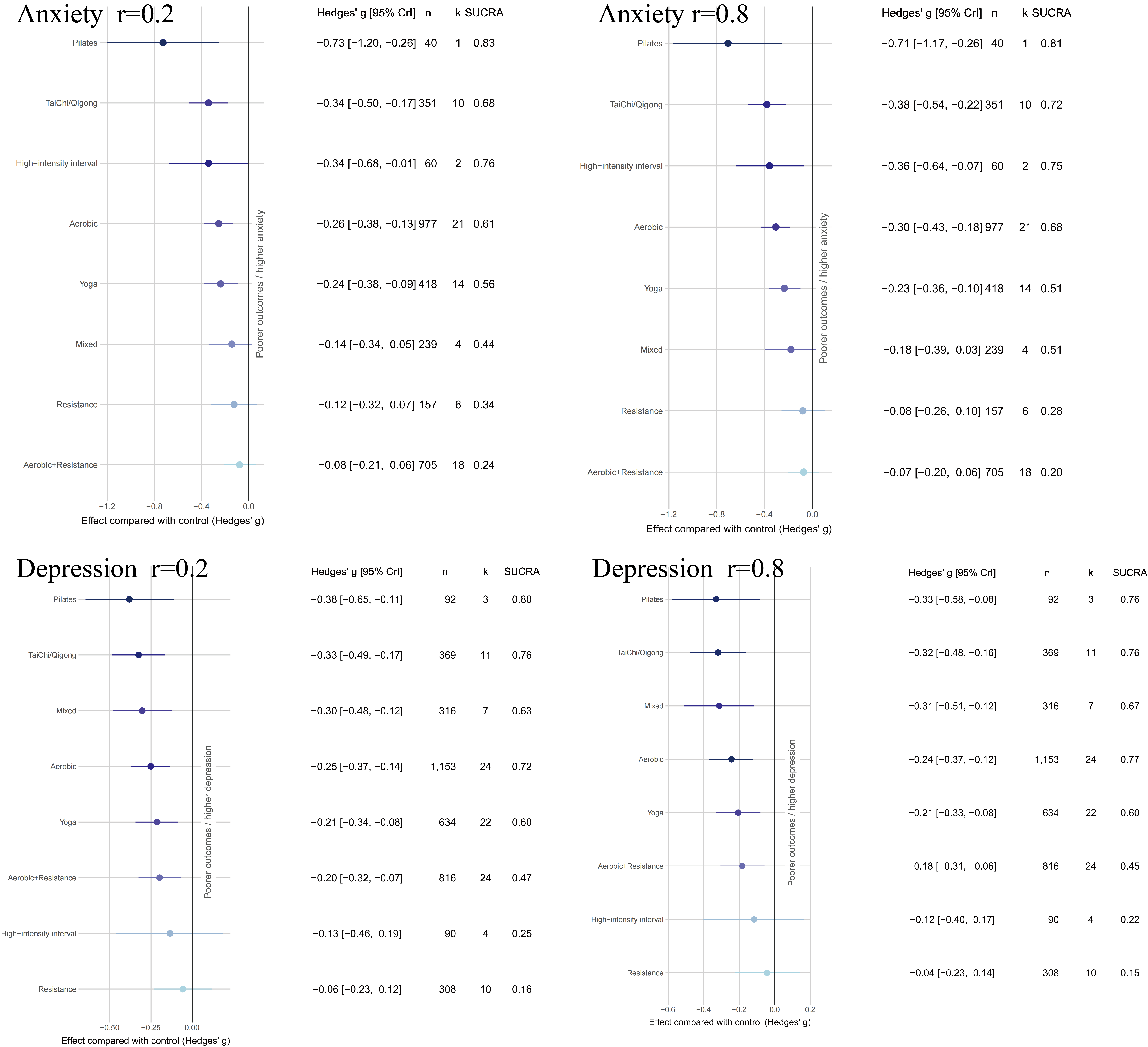
**

**Supplementary 12: Dose-response relationship (Depression)**

**Predicted intensity met (min) for exercise**

| intensity_met | pred | se | lower | upper |
| --- | --- | --- | --- | --- |
| 1.0 | -0.22615 | 0.1693 | -0.50462 | 0.05232 |
| 1.2 | -0.23716 | 0.14992 | -0.48375 | 0.00943 |
| 1.4 | -0.24816 | 0.13099 | -0.46362 | -0.03271 |
| 1.6 | -0.25917 | 0.11274 | -0.4446 | -0.07373 |
| 1.8 | -0.27013 | 0.09558 | -0.42735 | -0.11291 |
| 2.0 | -0.28073 | 0.08033 | -0.41286 | -0.1486 |
| 2.2 | -0.29034 | 0.06816 | -0.40246 | -0.17823 |
| 2.4 | -0.29817 | 0.06029 | -0.39734 | -0.19901 |
| 2.6 | -0.3034 | 0.05709 | -0.3973 | -0.2095 |
| 2.8 | -0.30526 | 0.05749 | -0.39981 | -0.2107 |
| 3.0 | -0.30318 | 0.05953 | -0.4011 | -0.20526 |
| 3.2 | -0.29693 | 0.06153 | -0.39815 | -0.19572 |
| 3.4 | -0.28654 | 0.06256 | -0.38944 | -0.18363 |
| 3.6 | -0.27225 | 0.06236 | -0.37482 | -0.16968 |
| 3.8 | -0.25455 | 0.06122 | -0.35524 | -0.15386 |
| 4.0 | -0.23417 | 0.05986 | -0.33263 | -0.13571 |
| 4.2 | -0.21199 | 0.0592 | -0.30936 | -0.11461 |
| 4.4 | -0.18897 | 0.06004 | -0.28771 | -0.09022 |
| 4.6 | -0.16605 | 0.0627 | -0.26918 | -0.06292 |
| 4.8 | -0.14408 | 0.06696 | -0.25421 | -0.03395 |
| 5.0 | -0.12374 | 0.07223 | -0.24254 | -0.00494 |
| 5.2 | -0.10555 | 0.07784 | -0.23359 | 0.02249 |
| 5.4 | -0.08984 | 0.08328 | -0.22682 | 0.04714 |
| 5.6 | -0.07681 | 0.08815 | -0.2218 | 0.06819 |
| 5.8 | -0.06665 | 0.09221 | -0.21832 | 0.08502 |
| 6.0 | -0.05954 | 0.09529 | -0.21628 | 0.09721 |
| 6.2 | -0.05551 | 0.09739 | -0.21571 | 0.10469 |
| 6.4 | -0.05448 | 0.09864 | -0.21673 | 0.10777 |
| 6.6 | -0.05626 | 0.09929 | -0.21958 | 0.10705 |
| 6.8 | -0.06056 | 0.09968 | -0.22451 | 0.10339 |
| 7.0 | -0.06699 | 0.10022 | -0.23185 | 0.09786 |
| 7.2 | -0.07519 | 0.10132 | -0.24184 | 0.09146 |
| 7.4 | -0.08475 | 0.1033 | -0.25466 | 0.08515 |
| 7.6 | -0.09527 | 0.1064 | -0.27028 | 0.07973 |
| 7.8 | -0.10639 | 0.1107 | -0.28848 | 0.0757 |
| 8.0 | -0.1178 | 0.11618 | -0.30891 | 0.0733 |

**Predicted session duration (min) for exercise**

| session duration | pred | se | lower | upper |
| --- | --- | --- | --- | --- |
| 10 | -0.08394 | 0.21958 | -0.44511 | 0.27723 |
| 15 | -0.13941 | 0.1729 | -0.4238 | 0.14498 |
| 20 | -0.19313 | 0.1302 | -0.4073 | 0.02103 |
| 25 | -0.24211 | 0.0959 | -0.39985 | -0.08437 |
| 30 | -0.28263 | 0.07547 | -0.40676 | -0.1585 |
| 35 | -0.31105 | 0.06981 | -0.42587 | -0.19623 |
| 40 | -0.32495 | 0.07102 | -0.44177 | -0.20813 |
| 45 | -0.32354 | 0.07099 | -0.4403 | -0.20678 |
| 50 | -0.30735 | 0.06701 | -0.41756 | -0.19713 |
| 55 | -0.27865 | 0.06137 | -0.3796 | -0.1777 |
| 60 | -0.24133 | 0.05955 | -0.33928 | -0.14338 |
| 65 | -0.19978 | 0.0653 | -0.30719 | -0.09238 |
| 70 | -0.15843 | 0.07651 | -0.28427 | -0.03258 |
| 75 | -0.12143 | 0.0883 | -0.26667 | 0.02381 |
| 80 | -0.09168 | 0.09766 | -0.25231 | 0.06895 |
| 85 | -0.07052 | 0.10454 | -0.24247 | 0.10144 |
| 90 | -0.05895 | 0.11105 | -0.24162 | 0.12371 |
| 95 | -0.05758 | 0.12128 | -0.25707 | 0.1419 |
| 100 | -0.0653 | 0.13996 | -0.29552 | 0.16492 |
| 105 | -0.08057 | 0.16922 | -0.35892 | 0.19777 |
| 110 | -0.10187 | 0.20816 | -0.44427 | 0.24052 |
| 115 | -0.12741 | 0.25443 | -0.54592 | 0.29109 |
| 120 | -0.15509 | 0.30534 | -0.65733 | 0.34716 |

**Predicted sessions (min) for exercise**

| sessions | pred | se | lower | upper |
| --- | --- | --- | --- | --- |
| 1.0 | -0.1149 | 0.11057 | -0.29676 | 0.06697 |
| 1.5 | -0.12183 | 0.07507 | -0.24531 | 0.00164 |
| 2.0 | -0.13555 | 0.06013 | -0.23445 | -0.03665 |
| 2.5 | -0.16173 | 0.06176 | -0.26332 | -0.06014 |
| 3.0 | -0.20172 | 0.06403 | -0.30704 | -0.0964 |
| 3.5 | -0.2544 | 0.06339 | -0.35866 | -0.15014 |
| 4.0 | -0.3132 | 0.06721 | -0.42376 | -0.20265 |
| 4.5 | -0.37022 | 0.07732 | -0.4974 | -0.24304 |
| 5.0 | -0.41773 | 0.08723 | -0.56121 | -0.27425 |
| 5.5 | -0.44949 | 0.09321 | -0.60281 | -0.29618 |
| 6.0 | -0.46504 | 0.10407 | -0.63621 | -0.29387 |
| 6.5 | -0.46644 | 0.13283 | -0.68493 | -0.24796 |
| 7.0 | -0.46022 | 0.18036 | -0.75688 | -0.16356 |

**Supplementary 13: Model fitting effect (Depression)**


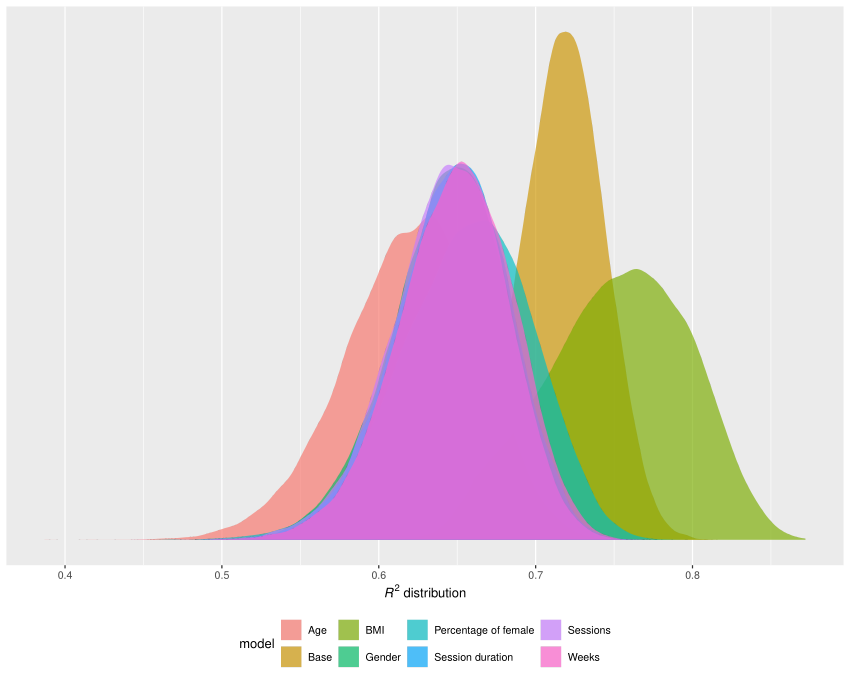


**Supplementary 14: Dose-response relationship (Anxiety)**

**Predicted intensity met (min) for exercise**

| intensity met | pred | se | lower | upper |
| --- | --- | --- | --- | --- |
| 1.0 | -0.47094 | 0.20795 | -0.81299 | -0.12889 |
| 1.2 | -0.44770 | 0.18733 | -0.75582 | -0.13958 |
| 1.4 | -0.42446 | 0.16679 | -0.69910 | -0.14982 |
| 1.6 | -0.40122 | 0.14701 | -0.64302 | -0.15942 |
| 1.8 | -0.37801 | 0.12764 | -0.58796 | -0.16807 |
| 2.0 | -0.35501 | 0.10932 | -0.53482 | -0.17520 |
| 2.2 | -0.33243 | 0.09266 | -0.48484 | -0.18003 |
| 2.4 | -0.31057 | 0.07844 | -0.43960 | -0.18154 |
| 2.6 | -0.28976 | 0.06754 | -0.40085 | -0.17866 |
| 2.8 | -0.27038 | 0.06056 | -0.37000 | -0.17076 |
| 3.0 | -0.25281 | 0.05738 | -0.34720 | -0.15843 |
| 3.2 | -0.23737 | 0.05699 | -0.33111 | -0.14362 |
| 3.4 | -0.22428 | 0.05808 | -0.31980 | -0.12875 |
| 3.6 | -0.21371 | 0.05956 | -0.31168 | -0.11574 |
| 3.8 | -0.20579 | 0.06087 | -0.30591 | -0.10567 |
| 4.0 | -0.20053 | 0.06191 | -0.30236 | -0.09870 |
| 4.2 | -0.19784 | 0.06293 | -0.30134 | -0.09433 |
| 4.4 | -0.19750 | 0.06435 | -0.30334 | -0.09166 |
| 4.6 | -0.19926 | 0.06649 | -0.30863 | -0.08989 |
| 4.8 | -0.20283 | 0.06949 | -0.31713 | -0.08852 |
| 5.0 | -0.20793 | 0.07323 | -0.32839 | -0.08747 |
| 5.2 | -0.21431 | 0.07745 | -0.34171 | -0.08691 |
| 5.4 | -0.22174 | 0.08186 | -0.35638 | -0.08710 |
| 5.6 | -0.23003 | 0.08619 | -0.37180 | -0.08826 |
| 5.8 | -0.23898 | 0.09024 | -0.38741 | -0.09054 |
| 6.0 | -0.24839 | 0.09384 | -0.40274 | -0.09404 |
| 6.2 | -0.25809 | 0.09688 | -0.41744 | -0.09874 |
| 6.4 | -0.26792 | 0.09936 | -0.43135 | -0.10449 |
| 6.6 | -0.27776 | 0.10139 | -0.44453 | -0.11099 |
| 6.8 | -0.28755 | 0.10319 | -0.45729 | -0.11782 |
| 7.0 | -0.29726 | 0.10504 | -0.47004 | -0.12448 |
| 7.2 | -0.30685 | 0.10722 | -0.48321 | -0.13049 |
| 7.4 | -0.31632 | 0.10997 | -0.49721 | -0.13544 |
| 7.6 | -0.32571 | 0.11348 | -0.51237 | -0.13904 |
| 7.8 | -0.33503 | 0.11785 | -0.52887 | -0.14118 |
| 8.0 | -0.34431 | 0.12307 | -0.54675 | -0.14188 |
| 8.2 | -0.35360 | 0.12907 | -0.56590 | -0.14130 |
| 8.4 | -0.36288 | 0.13575 | -0.58618 | -0.13959 |
| 8.6 | -0.37216 | 0.14302 | -0.60741 | -0.13692 |
| 8.8 | -0.38145 | 0.15079 | -0.62948 | -0.13342 |
| 9.0 | -0.39073 | 0.15899 | -0.65224 | -0.12922 |
| 9.2 | -0.40001 | 0.16755 | -0.67561 | -0.12442 |
| 9.4 | -0.40930 | 0.17643 | -0.69949 | -0.11910 |
| 9.6 | -0.41858 | 0.18577 | -0.72381 | -0.11335 |
| 9.8 | -0.42786 | 0.19494 | -0.74851 | -0.10722 |
| 10.0 | -0.43715 | 0.20451 | -0.77353 | -0.10077 |

**Predicted session duration (min) for exercise**

| duration min | pred | se | lower | upper |
| --- | --- | --- | --- | --- |
| 10.0 | -0.39076 | 0.16226 | -0.65766 | -0.12386 |
| 15.0 | -0.37728 | 0.13809 | -0.60442 | -0.15013 |
| 20.0 | -0.36369 | 0.11607 | -0.55461 | -0.17277 |
| 25.0 | -0.34983 | 0.09748 | -0.51017 | -0.18948 |
| 30.0 | -0.33548 | 0.0834 | -0.47266 | -0.19831 |
| 35.0 | -0.32046 | 0.0739 | -0.44201 | -0.19891 |
| 40.0 | -0.30465 | 0.06787 | -0.41628 | -0.19302 |
| 45.0 | -0.28803 | 0.0637 | -0.39280 | -0.18326 |
| 50.0 | -0.27068 | 0.06043 | -0.37008 | -0.17129 |
| 55.0 | -0.25282 | 0.05836 | -0.34881 | -0.15683 |
| 60.0 | -0.23479 | 0.05855 | -0.33109 | -0.13848 |
| 65.0 | -0.21694 | 0.06158 | -0.31824 | -0.11565 |
| 70.0 | -0.19965 | 0.06691 | -0.30970 | -0.08960 |
| 75.0 | -0.18324 | 0.07331 | -0.30381 | -0.06266 |
| 80.0 | -0.16792 | 0.08003 | -0.29956 | -0.03628 |
| 85.0 | -0.15376 | 0.08732 | -0.29738 | -0.01014 |
| 90.0 | -0.14081 | 0.09609 | -0.29886 | 0.01724 |
| 95.0 | -0.12906 | 0.10769 | -0.30620 | 0.04807 |
| 100.0 | -0.11837 | 0.12342 | -0.32137 | 0.08463 |
| 105.0 | -0.10853 | 0.14374 | -0.34496 | 0.12790 |
| 110.0 | -0.09936 | 0.16834 | -0.37626 | 0.17753 |
| 115.0 | -0.09066 | 0.19641 | -0.41373 | 0.23241 |
| 120.0 | -0.08218 | 0.22689 | -0.45539 | 0.29102 |

**Predicted sessions (min) for exercise**

| sessions | pred | se | lower | upper |
| --- | --- | --- | --- | --- |
| 1.0 | -0.22276 | 0.11613 | -0.41378 | -0.03174 |
| 1.5 | -0.19017 | 0.07477 | -0.31316 | -0.06719 |
| 2.0 | -0.17402 | 0.06322 | -0.27801 | -0.07003 |
| 2.5 | -0.18768 | 0.06984 | -0.30257 | -0.07280 |
| 3.0 | -0.23235 | 0.07203 | -0.35084 | -0.11387 |
| 3.5 | -0.30251 | 0.06994 | -0.41755 | -0.18747 |
| 4.0 | -0.37802 | 0.07767 | -0.50578 | -0.25027 |
| 4.5 | -0.43555 | 0.09488 | -0.59162 | -0.27948 |
| 5.0 | -0.45350 | 0.10786 | -0.63091 | -0.27608 |
| 5.5 | -0.41512 | 0.10872 | -0.59395 | -0.23629 |
| 6.0 | -0.32139 | 0.11025 | -0.50273 | -0.14004 |
| 6.5 | -0.18094 | 0.13879 | -0.40923 | 0.04736 |
| 7.0 | -0.01549 | 0.19938 | -0.34344 | 0.31246 |

**Supplementary 15: Model fitting effect (Anxiety)**


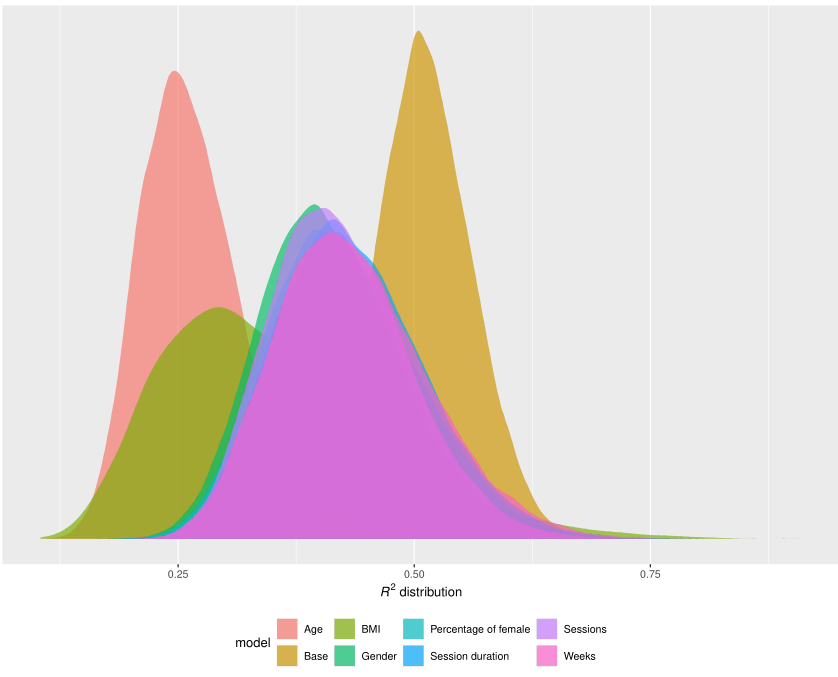


**Supplementary 16: GRADE Summary**

**Depression – GRADE Summary**

| **Intervention** | **Arms** | **Participants** | **Effect (g, 95% CrI)** | **Risk of bias** | **Inconsistency** | **Indirectness** | **Imprecision** | **Publication bias** | **Certainty** | **Reasons for downgrading** |
| --- | --- | --- | --- | --- | --- | --- | --- | --- | --- | --- |
| Aerobic | 24 | 1153 | -0.25 [-0.37, -0.13] | Serious | Not serious | Not serious | Not serious | Serious | Low | Risk of bias (randomization/ concealment & blinding limitations; fidelity/ adherence reporting) + suspected publication bias (asymmetric funnel; Egger positive). |
| Aerobic+Resistance | 24 | 816 | -0.19 [-0.32, -0.06] | Serious | Not serious | Not serious | Not serious | Serious | Low | Risk of bias (allocation concealment/ blinding/ measurement) + suspected publication bias across depression outcomes. |
| High-intensity interval | 4 | 90 | -0.13 [-0.43,+0.17] | Serious | Not serious | Not serious | Serious | Serious | Very low | Small evidence base and wide CrI crossing the null (information size unmet) + risk of bias + suspected publication bias. |
| Mixed | 7 | 316 | -0.31 [-0.50, -0.12] | Serious | Not serious | Serious | Not serious | Serious | Very low | Composite prescriptions with heterogeneous components/therapist contact (indirectness) + risk of bias + suspected publication bias. |
| Pilates | 3 | 92 | -0.35 [-0.61, -0.10] | Serious | Not serious | Not serious | Serious | Serious | Very low | Small total sample; wide CrI (OIS not met) + behavioral trial biases + suspected publication bias. |
| Resistance | 10 | 308 | -0.05 [-0.23,+0.13] | Serious | Not serious | Not serious | Serious | Serious | Very low | Effect close to null with CrI crossing 0 (imprecision) + risk of bias + suspected publication bias. |
| TaiChi/Qigong | 11 | 369 | -0.32 [-0.48, -0.17] | Serious | Not serious | Serious | Not serious | Serious | Very low | Regional/cultural adaptations limit generalizability (indirectness) + risk of bias + suspected publication bias. |
| Yoga | 22 | 634 | -0.21 [-0.34, -0.08] | Serious | Not serious | Not serious | Not serious | Serious | Low | Risk of bias allocation/blinding/ fidelity) + suspected publication bias (network-level asymmetry). |

**Anxiety– GRADE Summary**

| **Intervention** | **Arms** | **Participants** | **Effect (g, 95% CrI)** | **Risk of bias** | **Inconsistency** | **Indirectness** | **Imprecision** | **Publication bias** | **Certainty** | **Reasons for downgrading** |
| --- | --- | --- | --- | --- | --- | --- | --- | --- | --- | --- |
| Aerobic | 21 | 977 | -0.27 [-0.40, -0.15] | Serious | Not serious | Not serious | Not serious | Serious | Low | Risk of bias (randomization/allocation concealment often unclear; performance/measurement bias inherent to behavioral trials) + suspected publication bias (funnel asymmetry; Egger p<0.001). |
| Aerobic+Resistance | 18 | 705 | -0.07 [-0.20, +0.06] | Serious | Not serious | Not serious | Serious | Serious | Very low | Risk of bias + imprecision (CrI includes 0; overall information size likely insufficient for a small effect) + suspected publication bias (network-level asymmetry). |
| High-intensity interval | 2 | 60 | -0.36 [-0.66, -0.05] | Serious | Not serious | Not serious | Serious | Serious | Very low | Small evidence base (2 arms; n≈60) with wide CrI (information size unmet) + risk of bias + suspected publication bias. |
| Mixed | 4 | 239 | -0.16 [-0.35, +0.04] | Serious | Not serious | Not serious | Serious | Serious | Very low | Risk of bias + imprecision (CrI spans null) + suspected publication bias (small-study effects). |
| Pilates | 1 | 40 | -0.70 [-1.16, -0.26] | Serious | Not serious | Not serious | Serious | Serious | Very low | Single small trial (n≈40) with wide CrI (imprecision) + behavioral trial biases + suspected publication bias. |
| Resistance | 6 | 157 | -0.11 [-0.30, -0.07] | Serious | Not serious | Not serious | Serious | Serious | Very low | Effect close to null with CrI including 0 (imprecision) + risk of bias + suspected publication bias. |
| TaiChi/Qigong | 10 | 351 | -0.35 [-0.51, -0.19] | Serious | Not serious | Serious | Not serious | Serious | Very low | Risk of bias + indirectness (studies concentrated in specific regions/cultural adaptations) + suspected publication bias. |
| Yoga | 14 | 418 | -0.24 [-0.37, -0.10] | Serious | Not serious | Not serious | Not serious | Serious | Low | Risk of bias (allocation concealment/ blinding/ fidelity reporting) + suspected publication bias (Egger positive; network-level). |

**Reference**

Adams, Scott C, et al.

2018 Effects of high-intensity interval training on fatigue and quality of life in testicular cancer survivors. British Journal of Cancer 118(10):1313-1321.

Aydin, Mensure, et al.

2021 The effect of exercise on life quality and depression levels of breast cancer patients. Asian Pacific journal of cancer prevention: APJCP 22(3):725.

Boing, Leonessa, et al.

2023 Mat Pilates and belly dance: Effects on patient-reported outcomes among breast cancer survivors receiving hormone therapy and adherence to exercise. Complementary therapies in clinical practice 50:101683.

Bower, Julienne E, et al.

2012 Yoga for persistent fatigue in breast cancer survivors: a randomized controlled trial. Cancer 118(15):3766-3775.

Cadmus, Lisa A, et al.

2009 Exercise and quality of life during and after treatment for breast cancer: results of two randomized controlled trials. Psycho‐Oncology: Journal of the Psychological, Social and Behavioral Dimensions of Cancer 18(4):343-352.

Cantarero-Villanueva, Irene, et al.

2013 The effectiveness of a deep water aquatic exercise program in cancer-related fatigue in breast cancer survivors: a randomized controlled trial. Archives of physical medicine and rehabilitation 94(2):221-230.

Cartmel, Brenda, et al.

2021 Randomized trial of exercise on depressive symptomatology and brain derived neurotrophic factor (BDNF) in ovarian cancer survivors: The Women's Activity and Lifestyle Study in Connecticut (WALC). Gynecologic oncology 161(2):587-594.

Cavalheri, Vinicius, et al.

2017 Exercise training for people following curative intent treatment for non-small cell lung cancer: a randomized controlled trial. Brazilian journal of physical therapy 21(1):58-68.

Chang, Pi-Hua, et al.

2008 Effects of a walking intervention on fatigue-related experiences of hospitalized acute myelogenous leukemia patients undergoing chemotherapy: a randomized controlled trial. Journal of pain and symptom management 35(5):524-534.

Charati, Fahimeh Ghasemi, et al.

2022 Motor exercises effect on improving shoulders functioning, functional ability, quality of life, depression and anxiety for women with breast cancer. Clinical Breast Cancer 22(7):666-673.

Chen, HM, et al.

2015 Randomised controlled trial on the effectiveness of home-based walking exercise on anxiety, depression and cancer-related symptoms in patients with lung cancer. British journal of cancer 112(3):438-445.

Chen, Zhen, et al.

2013 Qigong improves quality of life in women undergoing radiotherapy for breast cancer: results of a randomized controlled trial. Cancer 119(9):1690-1698.

Cheng, Duan, et al.

2021 Effect of Tai Chi and resistance training on cancer-related fatigue and quality of life in middle-aged and elderly cancer patients. Chinese journal of integrative medicine 27(4):265-272.

Cheung, Denise Shuk Ting, et al.

2021 Feasibility of aerobic exercise and Tai-Chi interventions in advanced lung cancer patients: a randomized controlled trial. Integrative cancer therapies 20:15347354211033352.

Choi, Jin Yi, and Hyun Sook Kang

2012 Effects of a home-based exercise program for patients with stomach cancer receiving oral chemotherapy after surgery. Journal of Korean Academy of Nursing 42(1):95-104.

Cordier, Dominik, Markus Gerber, and Serge Brand

2019 Effects of two types of exercise training on psychological well-being, sleep, quality of life and physical fitness in patients with high-grade glioma (WHO III and IV): study protocol for a randomized controlled trial. Cancer Communications 39(1):46.

Courneya, Kerry S, et al.

2014 A multicenter randomized trial of the effects of exercise dose and type on psychosocial distress in breast cancer patients undergoing chemotherapy. Cancer epidemiology, biomarkers & prevention 23(5):857-864.

Courneya, Kerry S, et al.

2007 Effects of aerobic and resistance exercise in breast cancer patients receiving adjuvant chemotherapy: a multicenter randomized controlled trial. Journal of clinical oncology 25(28):4396-4404.

Cramer, Holger, et al.

2016 A randomized controlled bicenter trial of yoga for patients with colorectal cancer. Psycho‐oncology 25(4):412-420.

Culos-Reed, S Nicole, et al.

2010 Physical activity for men receiving androgen deprivation therapy for prostate cancer: benefits from a 16-week intervention. Supportive Care in Cancer 18(5):591-599.

da Silveira, Juliana, et al.

2025 Free dance proves to be effective in depressive symptoms, anxiety and stress in women undergoing breast cancer surgery: a randomized clinical trial. Journal of Dance Medicine & Science:1089313X251319570.

Danhauer, Suzanne C, et al.

2009 Restorative yoga for women with breast cancer: findings from a randomized pilot study. Psycho‐Oncology: Journal of the Psychological, Social and Behavioral Dimensions of Cancer 18(4):360-368.

de Souza, Júlia Cristina Barcelos, et al.

2025 What are the effects of free dance and dance therapy on self-esteem, anxiety, body image and depressive symptoms of women undergoing breast cancer surgery? A randomized clinical trial. Journal of Bodywork and Movement Therapies 42:1159-1167.

Díaz-Balboa, Estíbaliz, et al.

2024 Exercise-based cardio-oncology rehabilitation for cardiotoxicity prevention during breast cancer chemotherapy: The ONCORE randomized controlled trial. Progress in Cardiovascular Diseases 85:74-81.

Donnelly, C, et al.

2011a A randomised controlled trial testing the feasibility of a physical activity intervention in managing fatigue with gynaecological cancer survivors. Physiotherapy (United Kingdom) 97:eS294-eS295.

Donnelly, CM, et al.

2011b A randomised controlled trial testing the feasibility and efficacy of a physical activity behavioural change intervention in managing fatigue with gynaecological cancer survivors. Gynecologic oncology 122(3):618-624.

Dreyling, Esther, et al.

2025 A Randomized Controlled ‘REAL‐FITNESS’Trial to Evaluate Physical Activity in Patients With Newly Diagnosed Multiple Myeloma. Journal of cachexia, sarcopenia and muscle 16(2):e13793.

Dülger, Esra, et al.

2022 Effects of combined aerobic-strength training and yoga on quality of life and related parameters in women with pituitary adenoma after surgery: a randomized crossover study. European Journal of Endocrinology 186(6):667-675.

Eckert, Ryan, et al.

2022 A randomized pilot study of online hatha yoga for physical and psychological symptoms among survivors of allogenic bone marrow transplant. International journal of yoga therapy 32(2022):Article 12.

Egegaard, Trine, et al.

2019 Pre-radiotherapy daily exercise training in non-small cell lung cancer: A feasibility study. Reports of Practical Oncology and Radiotherapy 24(4):375-382.

Ergun, M, et al.

2013 Effects of exercise on angiogenesis and apoptosis‐related molecules, quality of life, fatigue and depression in breast cancer patients. European journal of cancer care 22(5):626-637.

Eyigor, Sibel, et al.

2010 Effects of pilates exercises on functional capacity, flexibility, fatigue, depression and quality of life in female breast cancer patients: a randomized controlled study. Eur J Phys Rehabil Med 46(4):481-7.

Eyigor, Sibel, et al.

2018 Can yoga have any effect on shoulder and arm pain and quality of life in patients with breast cancer? A randomized, controlled, single-blind trial. Complementary therapies in clinical practice 32:40-45.

Galvão, Daniel A, et al.

2021 Psychological distress in men with prostate cancer undertaking androgen deprivation therapy: modifying effects of exercise from a year-long randomized controlled trial. Prostate Cancer and Prostatic Diseases 24(3):758-766.

Gokal, Kajal, et al.

2016 Effects of a self-managed home-based walking intervention on psychosocial health outcomes for breast cancer patients receiving chemotherapy: a randomised controlled trial. Supportive Care in Cancer 24(3):1139-1166.

Golsteijn, Rianne Henrica Johanna, et al.

2018 Short-term efficacy of a computer-tailored physical activity intervention for prostate and colorectal cancer patients and survivors: a randomized controlled trial. International Journal of Behavioral Nutrition and Physical Activity 15(1):106.

Han, Jeehee, et al.

2023 Long term effects of a social capital-based exercise adherence intervention for breast cancer survivors with moderate fatigue: A randomized controlled trial. Integrative cancer therapies 22:15347354231209440.

Hardoerfer, Katrin, and Elisabeth Jentschke

2018 Effect of yoga therapy on symptoms of anxiety in cancer patients. Oncology research and treatment 41(9):526-532.

He, Xiaole, et al.

2022 Effects of a 16-week dance intervention on the symptom cluster of fatigue-sleep disturbance-depression and quality of life among patients with breast cancer undergoing adjuvant chemotherapy: a randomized controlled trial. International Journal of Nursing Studies 133:104317.

Ho, Rainbow TH, et al.

2016 Effects of a short-term dance movement therapy program on symptoms and stress in patients with breast cancer undergoing radiotherapy: a randomized, controlled, single-blind trial. Journal of pain and symptom management 51(5):824-831.

Jong, Miek C, et al.

2018 A randomized study of yoga for fatigue and quality of life in women with breast cancer undergoing (neo) adjuvant chemotherapy. The Journal of Alternative and Complementary Medicine 24(9-10):942-953.

Kamen, Charles, et al.

2016 A dyadic exercise intervention to reduce psychological distress among lesbian, gay, and heterosexual cancer survivors. LGBT health 3(1):57-64.

Kang, Dong-Woo, et al.

2022 A randomized trial of the effects of exercise on anxiety, fear of cancer progression and quality of life in prostate cancer patients on active surveillance. Journal of Urology 207(4):814-822.

Kim, Ji Young, et al.

2019 Effects of a 12-week home-based exercise program on quality of life, psychological health, and the level of physical activity in colorectal cancer survivors: a randomized controlled trial. Supportive Care in Cancer 27(8):2933-2940.

Knoerl, Robert, et al.

2022a Yoga for chronic chemotherapy-induced peripheral neuropathy pain: a pilot, randomized controlled trial. Journal of Cancer Survivorship 16(4):882-891.

Knoerl, Robert, et al.

2022b Exploring the impact of exercise and mind–body prehabilitation interventions on physical and psychological outcomes in women undergoing breast cancer surgery. Supportive Care in Cancer 30(3):2027-2036.

Lanctôt, Dominique, et al.

2016 The effects of the Bali Yoga Program (BYP-BC) on reducing psychological symptoms in breast cancer patients receiving chemotherapy: results of a randomized, partially blinded, controlled trial. Journal of Complementary and Integrative Medicine 13(4):405-412.

Larkey, Linda K, et al.

2025 Randomized controlled trial testing Tai Chi Easy/Qigong and Sham Qigong on breast cancer survivors’ fatigue and associated symptoms. Complementary Therapies in Clinical Practice 61:102014.

Levin, Gregory T, et al.

2018 Modality of exercise influences rate of decrease in depression for cancer survivors with elevated depressive symptomatology. Supportive Care in Cancer 26(5):1597-1606.

Li, Hongmei, et al.

2024 Improving physical and mental health in women with breast cancer undergoing anthracycline-based chemotherapy through wearable device-based aerobic exercise: a randomized controlled trial. Frontiers in Public Health 12:1451101.

Li, Lijun, et al.

2023 Intelligent physical activity versus modified behavioral activation in adolescent and young adult cancer patients with psychological distress: A randomized, controlled pilot trial. Cancer Medicine 12(2):1935-1948.

Liu, Weimin, et al.

2022 Effect of mindfulness yoga on anxiety and depression in early breast cancer patients received adjuvant chemotherapy: a randomized clinical trial. Journal of cancer research and clinical oncology 148(9):2549-2560.

Livingston, Patricia M, et al.

2015 Effects of a clinician referral and exercise program for men who have completed active treatment for prostate cancer: a multicenter cluster randomized controlled trial (ENGAGE). Cancer 121(15):2646-2654.

Loh, Kah Poh, et al.

2019 Effects of a home‐based exercise program on anxiety and mood disturbances in older adults with cancer receiving chemotherapy. Journal of the American Geriatrics Society 67(5):1005-1011.

Lu, Han-Bing, et al.

2024 Clinical indicators of effects of yoga breathing exercises on patients with lung cancer after surgical resection: a randomized controlled trial. Cancer Nursing 47(3):E151-E158.

Ma, Xing, et al.

2025 A pilot randomized controlled trial of a yoga program for alleviating cancer-related fatigue and psychological distress in women with gynecological cancer. European Journal of Oncology Nursing 74:102731.

Mariano, KOP, M Diniz, and ATS Santos

2015 Effect of exercises with Swiss ball previously applied to radiation therapy for breast cancer. Revista Neurociencias 23(1):55-61.

Mehnert, Anja, et al.

2011 Effects of a physical exercise rehabilitation group program on anxiety, depression, body image, and health-related quality of life among breast cancer patients. Oncology Research and Treatment 34(5):248-253.

Midtgaard, Julie, et al.

2011 Exercise may reduce depression but not anxiety in self-referred cancer patients undergoing chemotherapy. Post-hoc analysis of data from the ‘Body & Cancer’trial. Acta oncologica 50(5):660-669.

Milbury, Kathrin, et al.

2019 Pilot randomized, controlled trial of a dyadic yoga program for glioma patients undergoing radiotherapy and their family caregivers. Neuro-Oncology Practice 6(4):311-320.

Molassiotis, Alex, Dau Van Vu, and Shirley Siu Yin Ching

2021 The effectiveness of Qigong in managing a cluster of symptoms (Breathlessness-Fatigue-Anxiety) in patients with lung cancer: a randomized controlled trial. Integrative cancer therapies 20:15347354211008253.

Monga, Uma, et al.

2007 Exercise prevents fatigue and improves quality of life in prostate cancer patients undergoing radiotherapy. Archives of physical medicine and rehabilitation 88(11):1416-1422.

Moraes, Rafael F, et al.

2021 Resistance training, fatigue, quality of life, anxiety in breast cancer survivors. The Journal of Strength & Conditioning Research 35(5):1350-1356.

Mostafaei, Fatemeh, et al.

2021 Effect of exercise on depression and fatigue in breast cancer women undergoing chemotherapy: A randomized controlled trial. Heliyon 7(7).

Munsie, Claire, et al.

2022 A randomised controlled trial investigating the ability for supervised exercise to reduce treatment-related decline in adolescent and young adult cancer patients. Supportive Care in Cancer 30(10):8159-8171.

Mutrie, Nanette, et al.

2007 Benefits of supervised group exercise programme for women being treated for early stage breast cancer: pragmatic randomised controlled trial. Bmj 334(7592):517.

Nicole Culos‐Reed, S, et al.

2006 A pilot study of yoga for breast cancer survivors: physical and psychological benefits. Psycho‐Oncology: Journal of the Psychological, Social and Behavioral Dimensions of Cancer 15(10):891-897.

Niels, Timo, et al.

2025 Resistance training in cachectic pancreatic and lung cancer patients: randomised controlled trial. BMJ Supportive & Palliative Care.

Odynets, Tetiana, et al.

2019 Impact of different exercise interventions on anxiety and depression in breast cancer patients. Physiotherapy Quarterly 27(4):31-36.

Özkan, Emine Elif, Feray Soyupek, and Zeynep Türen

2022 THE PSYCHOLOGICAL AND PHYSICAL BENEFITS OF THE SUPERVISED INDIVIDUAL EXERCISE PROGRAM DURING RADIOTHERAPY IN BREAST CANCER PATIENTS: A RANDOMIZED STUDY. Medical Journal of Süleyman Demirel University 29(4):541-552.

Pieczyńska, Anna, et al.

2023 Rehabilitation exercises supported by monitor-augmented reality for patients with high-grade glioma undergoing radiotherapy: results of a randomized clinical trial. Journal of Clinical Medicine 12(21):6838.

Piraux, Elise, et al.

2021 Effects of high-intensity interval training compared with resistance training in prostate cancer patients undergoing radiotherapy: a randomized controlled trial. Prostate cancer and prostatic diseases 24(1):156-165.

Piraux, Elise, et al.

2022 High-intensity aerobic interval training and resistance training are feasible in rectal cancer patients undergoing chemoradiotherapy: a feasibility randomized controlled study. reports of practical Oncology and radiotherapy 27(2):198-208.

Quist, Morten, et al.

2020 Effects of an exercise intervention for patients with advanced inoperable lung cancer undergoing chemotherapy: a randomized clinical trial. Lung Cancer 145:76-82.

Raghavendra, Rao M, et al.

2009 Effects of a yoga program on cortisol rhythm and mood states in early breast cancer patients undergoing adjuvant radiotherapy: a randomized controlled trial. Integrative cancer therapies 8(1):37-46.

Rao, Raghavendra Mohan, et al.

2017 Effects of a yoga program on mood states, quality of life, and toxicity in breast cancer patients receiving conventional treatment: A randomized controlled trial. Indian journal of palliative care 23(3):237.

Ratcliff, Chelsea G, et al.

2016 Examining mediators and moderators of yoga for women with breast cancer undergoing radiotherapy. Integrative cancer therapies 15(3):250-262.

Rehman, Muheebur, et al.

2023 Effects of exercise training in patients with lung cancer during chemotherapy treatment. The Malaysian Journal of Medical Sciences: MJMS 30(2):141.

Rogers, Laura Q, et al.

2023 Physical activity intervention benefits persist months post-intervention: randomized trial in breast cancer survivors. Journal of Cancer Survivorship 17(6):1834-1846.

Salchow, Jannike L, et al.

2021 A randomized controlled pilot trial about the influence of Kyusho Jitsu exercise on self-efficacy, fear, depression, and distress of breast cancer patients within follow-up care. Integrative Cancer Therapies 20:15347354211037955.

Schmidt, Martina E, et al.

2015 Effects of resistance exercise on fatigue and quality of life in breast cancer patients undergoing adjuvant chemotherapy: a randomized controlled trial. International journal of cancer 137(2):471-480.

Steindorf, Karen, et al.

2014 Randomized, controlled trial of resistance training in breast cancer patients receiving adjuvant radiotherapy: results on cancer-related fatigue and quality of life. Annals of oncology 25(11):2237-2243.

Tock, Wing Lam, et al.

2024 Pilot Randomized Controlled Trial of Lymfit: A Theory-Guided Exercise Intervention for Young Adults with Lymphoma. Healthcare, 2024. Vol. 12, pp. 1101. MDPI.

Vargas-Román, Keyla, et al.

2022 Effect of a 16-session qigong program in Non-Hodgkin lymphoma survivors: A randomized clinical trial. Journal of Clinical Medicine 11(12):3421.

Wei, Xiaolin, et al.

2022 Effects of Baduanjin exercise on cognitive function and cancer-related symptoms in women with breast cancer receiving chemotherapy: a randomized controlled trial. Supportive Care in Cancer 30(7):6079-6091.

Wen, Liying, et al.

2023 Effects of Baduanjin exercise in nasopharyngeal carcinoma patients after chemoradiotherapy: a randomized controlled trial. Supportive Care in Cancer 31(1):79.

Wong, Sarah Suet Shan, Tai Wa Liu, and Shamay Sheung Mei Ng

2024 Effects of a tailor-made yoga program on upper limb function and sleep quality in women with breast cancer: A pilot randomized controlled trial. Heliyon 10(16).

Yagli, Naciye Vardar, and Ozlem Ulger

2015 The effects of yoga on the quality of life and depression in elderly breast cancer patients. Complementary therapies in clinical practice 21(1):7-10.

Yang, Li-Hua, et al.

2021 Qigong exercise for patients with gastrointestinal cancer undergoing chemotherapy and at high risk for depression: a randomized clinical trial. The Journal of Alternative and Complementary Medicine 27(9):750-759.

Yao, Li-Qun, et al.

2022 The effect of an evidence-based Tai chi intervention on the fatigue-sleep disturbance-depression symptom cluster in breast cancer patients: A preliminary randomised controlled trial. European Journal of Oncology Nursing 61:102202.

Ying, Wang, et al.

2019 The health effects of Baduanjin exercise (a type of Qigong exercise) in breast cancer survivors: a randomized, controlled, single-blinded trial. European Journal of Oncology Nursing 39:90-97.

Zetzl, Teresa, et al.

2021 Yoga effectively reduces fatigue and symptoms of depression in patients with different types of cancer. Supportive Care in Cancer 29(6):2973-2982.

Zhang, Huan, et al.

2023 Effect of Multimodal exercise on cancer-related fatigue in patients undergoing simultaneous radiotherapy and chemotherapy: a randomized trial in patients with breast cancer. Altern Ther Health Med 29(5):233-237.

Zhang, Jia-Yuan, et al.

2022 Effectiveness of a nurse-led Mindfulness-based Tai Chi Chuan (MTCC) program on Posttraumatic Growth and perceived stress and anxiety of breast cancer survivors. European journal of psychotraumatology 13(1):2023314.
